# Telomere length is not a main factor for the development of islet autoimmunity and type 1 diabetes in the TEDDY study

**DOI:** 10.1038/s41598-022-08058-7

**Published:** 2022-03-16

**Authors:** Carina Törn, Xiang Liu, Suna Onengut-Gumuscu, Kevin M. Counts, Jose Leonardo Moreno, Cassandra L. Remedios, Wei-Min Chen, Jonathon LeFaive, Martha D. Butterworth, Beena Akolkar, Jeffrey P. Krischer, Åke Lernmark, Marian Rewers, Jin-Xiong She, Jorma Toppari, Anette-Gabriele Ziegler, Aakrosh Ratan, Albert V. Smith, William A. Hagopian, Stephen S. Rich, Hemang M. Parikh, The TEDDY Study Group, Aaron Barbour, Kimberly Bautista, Judith Baxter, Daniel Felipe-Morales, Brigitte I. Frohnert, Marisa Stahl, Patricia Gesualdo, Rachel Haley, Michelle Hoffman, Rachel Karban, Edwin Liu, Alondra Munoz, Jill Norris, Stesha Peacock, Hanan Shorrosh, Andrea Steck, Megan Stern, Kathleen Waugh, Olli G. Simell, Annika Adamsson, Sanna-Mari Aaltonen, Suvi Ahonen, Mari Åkerlund, Leena Hakola, Anne Hekkala, Henna Holappa, Heikki Hyöty, Anni Ikonen, Jorma Ilonen, Sanna Jokipuu, Leena Karlsson, Jukka Kero, Miia Kähönen, Mikael Knip, Minna-Liisa Koivikko, Katja Kokkonen, Merja Koskinen, Mirva Koreasalo, Kalle Kurppa, Salla Kuusela, Jarita Kytölä, Sinikka Lahtinen, Jutta Laiho, Tiina Latva-aho, Laura Leppänen, Katri Lindfors, Maria Lönnrot, Elina Mäntymäki, Markus Mattila, Maija Miettinen, Katja Multasuo, Teija Mykkänen, Tiina Niininen, Sari Niinistö, Mia Nyblom, Sami Oikarinen, Paula Ollikainen, Zhian Othmani, Sirpa Pohjola, Jenna Rautanen, Anne Riikonen, Minna Romo, Satu Simell, Aino Stenius, Päivi Tossavainen, Mari Vähä-Mäkilä, Eeva Varjonen, Riitta Veijola, Irene Viinikangas, Suvi M. Virtanen, Desmond Schatz, Diane Hopkins, Leigh Steed, Jennifer Bryant, Katherine Silvis, Michael Haller, Melissa Gardiner, Richard McIndoe, Ashok Sharma, Stephen W. Anderson, Laura Jacobsen, John Marks, Paula D. Towe, Ezio Bonifacio, Cigdem Gezginci, Anja Heublein, Eva Hohoff, Sandra Hummel, Annette Knopff, Charlotte Koch, Sibylle Koletzko, Claudia Ramminger, Roswith Roth, Jennifer Schmidt, Marlon Scholz, Joanna Stock, Katharina Warncke, Lorena Wendel, Christiane Winkler, Daniel Agardh, Carin Andrén Aronsson, Maria Ask, Rasmus Bennet, Corrado Cilio, Susanne Dahlberg, Malin Goldman Tsubarah, Emelie Ericson-Hallström, Annika Björne Fors, Lina Fransson, Thomas Gard, Monika Hansen, Susanne Hyberg, Berglind Jonsdottir, Helena Elding Larsson, Marielle Lindström, Markus Lundgren, Marlena Maziarz, Maria Månsson Martinez, Jessica Melin, Zeliha Mestan, Caroline Nilsson, Yohanna Nordh, Kobra Rahmati, Anita Ramelius, Falastin Salami, Anette Sjöberg, Ulrika Ulvenhag, Terese Wiktorsson, Åsa Wimar, Michael Killian, Claire Cowen Crouch, Jennifer Skidmore, Christian Chamberlain, Brelon Fairman, Arlene Meyer, Jocelyn Meyer, Denise Mulenga, Nole Powell, Jared Radtke, Shreya Roy, Davey Schmitt, Sarah Zink, Dorothy Becker, Margaret Franciscus, MaryEllen Dalmagro-Elias Smith, Ashi Daftary, Mary Beth Klein, Chrystal Yates, Rajesh Adusumali, Sarah Austin-Gonzalez, Maryouri Avendano, Sandra Baethke, Brant Burkhardt, Nicholas Cadigan, Joanna Clasen, Laura Gandolfo, Jennifer Garmeson, Veena Gowda, Belinda Hsiao, Christina Karges, Shu Liu, Kristian F. Lynch, Jamie Malloy, Cristina McCarthy, Michael Shaffer, Susan Smith, Noah Sulman, Roy Tamura, Dena Tewey, Michael Toth, Ulla M. Uusitalo, Kendra Vehik, Ponni Vijayakandipan, Melissa Wroble, Jimin Yang, Kenneth Young, Michael Abbondondolo, Lori Ballard, Rasheedah Brown, David Cuthbertson, Stephen Dankyi, Christopher Eberhard, Steven Fiske, David Hadley, Kathleen Heyman, Francisco Perez Laras, Hye-Seung Lee, Qian Li, Colleen Maguire, Wendy McLeod, Aubrie Merrell, Steven Meulemans, Ryan Quigley, Laura Smith, Liping Yu, Dongmei Miao, Kathleen Gillespie, Alistair Williams, Kyla Chandler, Ilana Kelland, Yassin Ben Khoud, Matthew Randell, Emily Farber, Rebecca Roche Pickin, Jonathan Davis, Jordan Davis, Dan Gallo, Jessica Bonnie, Paul Campolieto, Christian Chamberlain, Jared Radtke, Sarah Zink, Previously Henry Erlich, Steven J. Mack, Anna Lisa Fear, Sandra Ke, Niveen Mulholland, Thomas Briese, Todd Brusko, Suzanne Bennett Johnson, Eoin F. McKinney, Tomi Pastinen, Eric W. Triplett

**Affiliations:** 1 Unit for Diabetes and Celiac Disease, Wallenberg/CRC, Department of Clinical Sciences, Lund University/CRC, Skåne University Hospital SUS, 21428 Malmö, Sweden; 2 Health Informatics Institute, Morsani College of Medicine, University of South Florida, 3650 Spectrum Blvd #100, Tampa, FL 33612 USA; 3 Center for Public Health Genomics, University of Virginia, Charlottesville, VA USA; 4 Department of Biostatistics, University of Michigan, Ann Arbor, MI USA; 5 National Institutes of Diabetes and Digestive and Kidney Diseases, Bethesda, MD USA; 6 Barbara Davis Center for Childhood Diabetes, University of Colorado, Aurora, CO USA; 7 Center for Biotechnology and Genomic Medicine, Medical College of Georgia, Augusta University, Augusta, GA USA; 8 Department of Pediatrics, Turku University Hospital, Turku, Finland; 9 Institute of Biomedicine, Research Centre for Integrative Physiology and Pharmacology and Centre for Population Health Research, University of Turku, Turku, Finland; 10 Institute of Diabetes Research, Helmholtz Zentrum München, Munich, Germany; 11 Forschergruppe Diabetes, Technical University of Munich, Klinikum Rechts der Isar, Munich, Germany; 12 Forschergruppe Diabetes e.V. at Helmholtz Zentrum München, Munich, Germany; 13 Pacific Northwest Diabetes Research Institute, Seattle, WA USA; 14 Tampere University, Tampere, Finland; 15 University of Oulu, Oulu, Finland; 16 Tampere University Hospital, Tampere, Finland; 17 Oulu University Hospital, Oulu, Finland; 18 Finnish Institute for Health and Welfare, Helsinki, Finland; 19 University of Kuopio, Kuopio, Finland; 20 Pediatric Endocrinology, University of Florida, Gainesville, FL USA; 21 Pediatric Endocrine Associates, Atlanta, GA USA; 22 Center for Regenerative Therapies, TU Dresden, Dresden, Germany; 23 Department of Nutritional Epidemiology, University of Bonn, Bonn, Germany; 24 Department of Gastroenterology, Dr. Von Hauner Children’s Hospital, Ludwig Maximillians University Munich, Munich, Germany; 25 Children’s Hospital of Pittsburgh of UPMC, Pittsburgh, PA USA; 26 Barbara Davis Center for Childhood Diabetes, University of Colorado Denver, Denver, CO USA; 27 Bristol Medical School, University of Bristol, Bristol, UK; 28 Center for Genetics, Children’s Hospital Oakland Research Institute, Oakland, CA USA; 29 NIDDK Biosample Repository at Fisher BioServices, Germantown, MD USA; 30 Columbia University Medical Center, Columbia University, New York, NY USA; 31 Florida State University College of Medicine, Tallahassee, FL USA; 32 Department of Medicine, University of Cambridge School of Clinical Medicine, Cambridge, UK; 33 Cambridge Institute of Therapeutic Immunology and Infectious Disease, Jeffrey Cheah Biomedical Centre, Cambridge, UK; 34 Cambridge Centre for Artificial Intelligence in Medicine, University of Cambridge, Cambridge, UK; 35 The Children’s Mercy Hospital, Kansas City, MO USA; 36 Microbiology and Cell Science Department, Institute for Food and Agricultural Sciences, University of Florida, Gainesville, FL USA

**Keywords:** Computational biology and bioinformatics, Genetics, Endocrinology

## Abstract

The Environmental Determinants of Diabetes in the Young (TEDDY) study enrolled 8676 children, 3–4 months of age, born with HLA-susceptibility genotypes for islet autoimmunity (IA) and type 1 diabetes (T1D). Whole-genome sequencing (WGS) was performed in 1119 children in a nested case–control study design. Telomere length was estimated from WGS data using five tools: Computel, Telseq, Telomerecat, qMotif and Motif_counter. The estimated median telomere length was 5.10 kb (IQR 4.52–5.68 kb) using Computel. The age when the blood sample was drawn had a significant negative correlation with telomere length (*P* = 0.003). European children, particularly those from Finland (*P* = 0.041) and from Sweden (*P* = 0.001), had shorter telomeres than children from the U.S.A. Paternal age (*P* = 0.019) was positively associated with telomere length. First-degree relative status, presence of gestational diabetes in the mother, and maternal age did not have a significant impact on estimated telomere length. HLA-DR4/4 or HLA-DR4/X children had significantly longer telomeres compared to children with HLA-DR3/3 or HLA-DR3/9 haplogenotypes (*P* = 0.008). Estimated telomere length was not significantly different with respect to any IA (*P* = 0.377), IAA-first (*P* = 0.248), GADA-first (*P* = 0.248) or T1D (*P* = 0.861). These results suggest that telomere length has no major impact on the risk for IA, the first step to develop T1D. Nevertheless, telomere length was shorter in the T1D high prevalence populations, Finland and Sweden.

## Introduction

Telomeres consist of repetitive nucleoprotein tracts of sequence (TTAGGG)n that cap the ends of linear chromosomes. Telomeres prevent the loss of coding sequences and hinder major chromosomal rearrangements^1^. Each cell division causes progressive attrition (around 50–200 bp of the telomeres) and is part of determining the lifespan of cells^2^. Rapidly dividing cell types are those within the immune system that proliferate not only to self-renew but also to perform their biological function.

Telomere length is, in part, inherited with a stronger influence on variation in telomere length from the father, defined by the stronger correlation in telomere length between father-son and father-daughter^3,4^. In addition, paternal age is positively correlated with longer telomeres^5^. At the population level, there is no difference in telomere length at birth between boys and girls^6^; however, in young children^7^ and young adults (around 30 years), telomeres are longer in women than in men, although the attrition rate is higher in women^8^. The preservation of telomeres in women may, therefore, occur during the first two decades of life^9^.

In healthy subjects, HLA-DRB1*04 alleles have been shown to be associated with shorter telomeres in CD4^+^ T-cells, although the difference in telomere length between HLA-DR4^+^ and HLA-DR4^-^ subjects was not identified at birth. Thus, during the first 20 years of life, telomere attrition may be accelerated in subjects with HLA-DR4^+^^10^.

Several mechanisms may contribute to the attrition of telomeres, but there is only one known mechanism in healthy individuals, beyond embryogenesis, that counteracts the shortening of telomeres. Some cell types, such as lymphocytes, are capable of activating telomerase, an enzyme that can elongate telomere sequences and thereby modulate cellular lifespan. Certain hormones can also stimulate telomerases. For example, estrogens have been shown to activate telomerase activity^11^ while cortisol can decrease telomerase activity. In vitro experiments have shown that high concentrations of hydrocortisone (comparable to cortisol levels that may occur in vivo due to stress) can reduce telomerase activity by up to 50%^12^. Women with gestational diabetes (GDM), a condition of altered glucose metabolism and hormonal patterns during pregnancy, is associated with shorter telomeres in girls born to GDM mothers, but not in boys^13^.

Telomere length exhibits disease-specific patterns in different autoimmune diseases^2,14,15^. Shorter telomere length in leukocytes has been reported in subjects with type 1 diabetes (T1D) compared to controls, although shorter telomere length was not associated with the duration of T1D^16^. In T1D, all-cause mortality is also associated with a shorter telomere length^17^.

Here, we utilize the TEDDY nested case–control (NCC) cohort to estimate telomere length from whole-genome sequencing (WGS) data. Analyses of telomere length were performed to find out if there was a relation to the development of islet autoimmunity (IA), type 1 diabetes (T1D), or both, as well as its association with HLA-DR-DQ haplogenotypes. Primary end-points in TEDDY included any IA, micro insulin autoantibody (mIAA)-first, the 65 kDa glutamate decarboxylase autoantibody (GADA)-first, or T1D.

## Methods

### Study design

Screening of 421,047 newborn children for high-risk genotypes was performed at six clinical sites, three in Europe (Finland, Germany and Sweden) and three in the U.S.A. (Colorado (CO), Georgia (GA) and Washington (WA)) starting on 1 September 2004 and ending on 28 February 2010 as previously reported^18^. The high-risk human leukocyte antigen (HLA)-haplogenotypes were as follows: HLA-DR3/4, HLA-DR4/4, HLA-DR4/8 or HLA-DR3/3 (Supplementary Table S1). HLA-DRB1*04 subtyping was performed to exclude those from the general population with HLA-DRB1*04:03. HLA-DQB1*03:04 also qualified for inclusion into the TEDDY study. HLA subtyping was not performed, so there was an inability to distinguish between HLA-DQB1*02 and HLA-DQA1*03 subtypes. In the HLA-DQB1*05:01 subtype, only HLA-DRB1*01 was included, while HLA-DRB1*10 was excluded.

The first blood sample for HLA-screening was obtained either as cord blood in the maternity clinic or as a dry blood spot (DBS) on day three or four. If the child was eligible for TEDDY, the family was contacted by a study nurse and invited to participate in the 15-year follow-up study with blood sampling for determining islet autoantibody (GADA, islet antigen-2 antibody (IA-2A) and mIAA) status every three months between 3 and 48 months of child’s age and every six months thereafter (while a child with islet autoantibody positivity remained on the three months visit schedule). HLA-haplogenotypes were confirmed in a second blood sample at the 9-month visit. For the present study, blood samples were taken at the median age of 0.82 years (interquartile range (IQR) 0.75–5.43 years) for extraction of DNA and subsequent sequencing.

All procedures performed in studies involving human participants were in accordance with the ethical standards of the institutional and/or national research committees and with the 1964 Helsinki declaration and its later amendments or comparable ethical standards. All procedures were approved by the ethics committees/institutional review boards including Colorado Multiple Institutional Review Board (04-0361); Medical College of Georgia Human Assurance Committee (2004–2010)/Georgia Health Sciences University Human Assurance Committee (2011–2012)/Georgia Regents University Institutional Review Board (2013–2017)/Augusta University Institutional Review Board (2017-present) (HAC 0405380); University of Florida Health Center Institutional Review Board (IRB201600277); Washington State Institutional Review Board (2004–2012)/Western Institutional Review Board (2013–present) (20130211); Ethics Committee of the Hospital District of Southwest Finland (Dnro168/2004); Bayerischen Landesärztekammer (Bavarian Medical Association) Ethics Committee (04089); and Regional Ethics Board in Lund, Section 2 (2004–2012)/Lund University Committee for Continuing Ethical Review (2013-present) (217/2004). In addition, TEDDY is monitored by an external evaluation committee formed by the National Institutes of Health, Bethesda, MD, U.S.A. Informed consent was obtained for all individual participants included in the study from a parent or primary caretaker, separately for genetic screening and participation in the prospective follow-up and WGS.

### Study outcomes: IA and T1D

The primary outcome of the TEDDY study is the development of persistent confirmed IA assessed every three months. In the U.S.A., all sera were assayed at the Barbara Davis Center for Childhood Diabetes at the University of Colorado, Denver, CO; in Europe all sera were assayed at the University of Bristol, Bristol, U.K. GADA, IA-2A and mIAA were all measured using radio-binding assays^19–21^. IA was confirmed if an autoantibody was identified in a sample at both Reference Laboratories. All samples positive for IA and 5% of negative samples were re-tested for confirmation in both Reference Laboratories.

Persistent autoimmunity was defined by the presence of confirmed IA (GADA, IA-2A or mIAA) on two or more consecutive visits. The date of persistent confirmed IA was defined as the first blood draw date at which the child was found positive for at least one islet autoantibody. As children may be born with maternal IA, positive results due to maternal transmission via placenta were excluded when defining the child’s IA status. If the child was IA-positive at 3 or 6 months of age, the IA status of the mother was assessed at the 9-month visit in order to distinguish maternal autoantibody from IA in the child. If maternal autoantibody was present, the child was not considered persistently IA-positive unless the child had a negative sample prior to the first positive sample.

The threshold for positivity differed by the laboratory. Positivity was defined by Bristol (GADA ≥ 33 DK-U/mL, IA-2A ≥ 5 DK-U/mL and mIAA > 0.95 Local Units) and Denver (GADA > 20 DK-U/mL, IA-2A > 5 DK-U/mL and mIAA > 0.010 index). Both Reference Laboratories have shown high sensitivity and specificity as well as concordance for defining the presence of confirmed IA^10,21^.

### HLA typing

HLA genotype screening was performed using either a dried blood spot (DBS) punch or a small volume blood specimen format^18,22^. After polymerase chain reaction (PCR) amplification of exon 2 of the HLA class II genes (*DRB1*, *DQA1* or *DQB1*), alleles were identified either by direct sequencing, oligonucleotide probe hybridization or other genotyping techniques. Typing to certify specific HLA-DR-DQ haplogenotypes was determined for each clinical center. Confirmation of the HLA haplogenotypes was performed by the central HLA Reference Laboratory at Roche Molecular Systems, Oakland, CA on the eligible subjects using the 9-month sample.

### Estimation of telomere length from the WGS data

WGS was performed on the subjects in two nested case–control (NCC) studies: one for IA and the other for T1D (Table 1). In the NCC study, the “cases” were those subjects that had developed any IA or T1D as of 30 June 2018 and “controls” were randomly selected from those autoantibody-negative or non-T1D children in the index case’s risk set, matched for sex, clinical site and first-degree relative (FDR) status^23^. Matching was performed in a 1:1 format (one control was matched to each case).

**Table 1:** Length of telomeres (kb) in study subjects by the status of islet autoimmunity (IA) and type 1 diabetes (T1D) in the Environmental Determinants of Diabetes in the Young (TEDDY) study.

Parameter	Islet autoimmunity (IA)			Type 1 diabetes (T1D)		
	Matched pairs n (%)	IA cases (in kb)	IA controls (in kb)	Matched pairs n (%)	T1D cases (in kb)	T1D controls (in kb)
**Country**						
Finland	63 (16.20%)	5.07 ± 0.76	5.22 ± 0.77	23 (19.49%)	5.09 ± 0.78	5.02 ± 0.93
Germany	13 (3.34%)	5.13 ± 0.65	5.52 ± 1.08	5 (4.24%)	5.39 ± 0.96	5.40 ± 0.27
Sweden	182 (46.78%)	5.13 ± 0.76	4.94 ± 0.84	49 (41.53%)	5.22 ± 0.87	5.05 ± 1.05
U.S.A.	131 (33.68%)	5.24 ± 0.78	5.35 ± 0.84	41 (34.74%)	5.16 ± 0.73	5.09 ± 0.84
**Family history of T1D**						
First-degree relative	79 (20.31%)	5.19 ± 0.64	5.19 ± 0.81	25 (21.19%)	5.27 ± 0.84	5.07 ± 0.81
General population	310 (79.69%)	5.15 ± 0.79	5.13 ± 0.87	93 (78.81%)	5.16 ± 0.80	5.07 ± 0.96
**Sex**						
Female	169 (43.44%)	5.23 ± 0.73	5.21 ± 0.88	52 (44.07%)	5.28 ± 0.75	5.09 ± 0.96
Male	220 (56.56%)	5.10 ± 0.78	5.10 ± 0.84	66 (55.93%)	5.10 ± 0.84	5.06 ± 0.91

Telomere length data were available on both case and matched control subjects in 389 IA pairs and 118 T1D pairs. Computel was used to estimate the telomere length from the whole-genome sequence (WGS) data.

Telomere length is presented as mean ± standard deviation (S.D.) in kb.

Extraction of DNA was performed by the Center for Public Health Genomics at the University of Virginia, VA, U.S.A. The WGS data were generated on the Illumina HiSeq X Series platform with paired-end 2 × 150 bp reads with targeted 30× coverage by Macrogen, Inc, Rockville, MD, U.S.A. FastQC, MultiQC^24^ and FastQ Screen^25^ were used to identify low-quality sequences and contaminants in the raw sequence data. The raw sequences were aligned to the Genome Reference Consortium Build 37 (GRCh37d5) for qMotif and to the Genome Reference Consortium Build 38 (GRCh38DH) for Computel, Telseq, Telomerecat and Motif_counter. Post-alignment processing was performed by utilizing the University of Michigan’s (UM) Docker-alignment pipeline (https://github.com/statgen/docker-alignment) based on Burrows-Wheeler Aligner (BWA)^26^. Variant concordance analysis was performed by comparing the variants identified from the WGS data to the T1DExomeChip array (Illumina, CA) data to determine mislabeled, swapped or contaminated samples.

Estimation of the telomere length from the WGS data was performed using five different tools: Computel, Telseq, Telomerecat, qMotif and Motif_counter (Supplementary methods). Pairwise Spearman correlation analysis was used to compare performance of these tools against each other. All subsequent statistical analysis on telomere length was conducted using estimates from Computel which is based on aligning raw sequence data in FASTQ format to a telomeric reference sequence.

### SNPs associated with the telomere length

**Figure 1:**
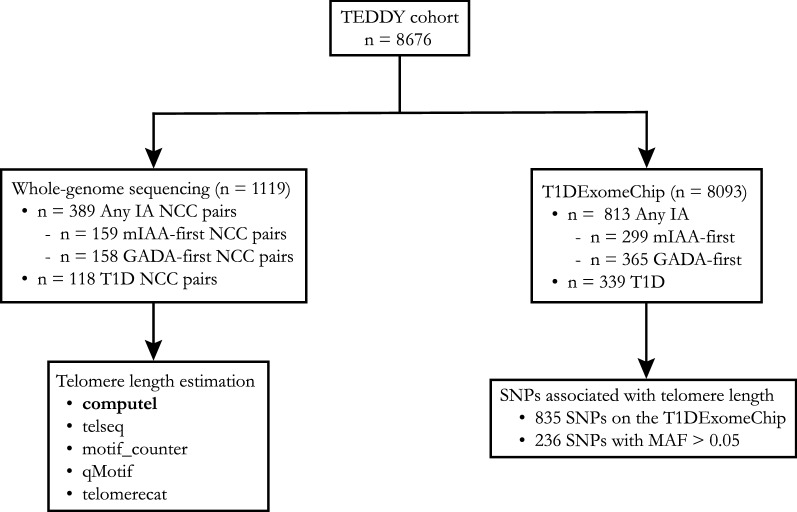
Whole-genome sequencing (WGS) was performed on the subjects in the nested case–control (NCC) study for islet autoimmunity (IA) and type 1 diabetes (T1D). In the NCC study, the “cases” were those subjects that had developed any IA (n = 389) or T1D (n = 118) and “controls” were randomly selected from those autoantibody-negative or non-T1D children in the index case’s risk set, matched for sex, clinical site and first-degree relative (FDR) status. Telomere length estimation was performed using five different tools. Single nucleotide polymorphisms (SNPs) were genotyped using the T1DExomeChip array. A total of 835 SNPs associated with telomere length were available on the T1DExomeChip and 236 of those had a minor allele frequency (MAF) > 0.05. SNP analysis was performed on 8093 TEDDY children.

Single nucleotide polymorphisms (SNPs) were genotyped by the Center for Public Health Genomics at the University of Virginia, VA, U.S.A., using the T1DExomeChip array on the full TEDDY cohort (n = 8093) (Fig. 1). The T1DExomeChip is a custom genotyping array with more than 90,000 custom content SNPs added to the Infinium® CoreExome-24 v.1.1 BeadChip (Illumina, CA). Genotype calling and the following quality control steps were applied to the dataset: (1) individuals with low call rate (< 95%), or discordance with reported sex and prior genotyping were not considered in the analysis, (2) SNPs with low call rates (< 95%) were excluded, (3) SNPs from autosomal chromosomes with allele distributions strongly deviating from Hardy–Weinberg equilibrium in controls with the European ancestry (*P* < 10^–6^) were discarded (except for chromosome 6 with the Hardy–Weinberg equilibrium in controls with the European ancestry (*P* < 10^–20^) due to HLA eligibility requirements), (4) SNPs on chromosome X were excluded with heterozygosity > 1% in men, (5) SNPs on chromosome Y were excluded with genotypes > 2% in women, (6) Monomorphic SNPs were excluded, (7) SNPs were discarded for which the concordance rate among duplicates was < 99% and (8) SNPs with missingness difference between IA or T1D cases and controls subjects assessed by the Fisher's exact test (*P* < 10^–4^). From literature, there were 835 SNPs associated with telomere length on the T1DExomeChip^27–38^.

### Statistical analyses

Multiple linear regression analysis was performed to examine the association between the telomere length and other factors including HLA-categories (HLA-DR3/3 or HLA-DR3/9 (as reference), HLA-DR3/4 and HLA-DR4/4, HLA-DR4/X), sex, parental age, mother´s gestational diabetes status, FDR status and the country of residence. The age at sample collection and the indicator of the long-distance protocol sample collection (yes vs. no) were also included in the model. Potential variation introduced by batches was taken account by a random intercept. Telomere length data were available on a total of 1119 TEDDY children.

Primary analyses were focused on examining the association between telomere length with the risk of any IA and/or T1D. A conditional logistic regression model was used to consider the matching criteria in the NCC study for any IA or T1D, adjusting for the HLA-categories (HLA-DR3/3 or HLA-DR3/9 (as reference), HLA-DR3/4 and HLA-DR4/4, HLA-DR4/X). In the analysis, telomere length was age-adjusted by using the residuals from the linear regression of telomere length on the age at sample collection. Subgroup analyses were conducted with respect to the type of first appearing autoantibody (mIAA-first and GADA-first). The magnitude of the association was estimated by odds ratio (OR), with 95% confidence internal (CI).

Secondary analyses were performed to examine the effect of SNPs associated with telomere length on the risk of any IA, mIAA-first, GADA-first and T1D (Supplementary Table S2). A total of 236 SNPs with minor allele frequency (MAF) > 0.05 were included (Fig. 1). Cox proportional hazard models were used to study the association between each SNP and the risk of any IA, mIAA-first, GADA-first and T1D, respectively, adjusting for sex, the country of residence, FDR, HLA-haplogenotype and accounting for population stratification (ancestral heterogeneity) (using the top three principal components estimated from the T1DExomeChip). Of the 8676 enrolled children, 583 were excluded: indeterminate autoantibody statuses (n = 14), HLA ineligible (n = 112), and no SNP data available (n = 457). A total of 7093 families had one child, 476 families had 2 children, and 16 families had 3 children who were included in the analysis. A robust variance estimate was used to account for the dependence within families in the Cox proportional hazard model^39^. The magnitude of each estimated association was defined by the Hazard ratio (HR), with 95% confidence intervals (CIs). To correct for multiple hypothesis testing, the false discovery rate adjusted *P*-values were calculated^40^. SNPs with nominal significance can be found in Supplementary Table S4 through S7.

Statistical analyses were performed using Statistical Analysis Software (Version 9.4, SAS Institute, Cary, North Carolina, U.S.A.) and R (Version 4.0.2). For the multiple linear regression analysis, estimates with *P* < 0.05 were considered as statistically significant.

## Results

Telomere lengths from the WGS data were calculated from 1119 TEDDY children (Fig. 1). Descriptive data on the estimation of the telomere lengths is shown by the status of IA and T1D in Table 1. The estimated median telomere length was 5.10 kb (IQR 4.52–5.68 kb) using Computel. The estimations of the telomere lengths were strongly correlated between Computel and Telseq (r = 0.97, *P* < 0.001) as well as between Computel and Motif_counter (r = 0.93, *P* < 0.001) (Supplemental Fig. S1).

### Association between telomere length and other phenotypes

The age of the child when the sample was drawn for WGS had a significant impact on the length of telomeres. There was a significant negative association between age of the children and telomere length (*P* = 0.003) (Table 2). Children that carried HLA-DR4/4 or HLA-DR4/X had significantly longer telomeres compared to children with HLA-DR3/3 or HLA-DR3/9 haplogenotypes (*P* = 0.008). European children, particularly those from Finland (*P* = 0.041) and from Sweden (*P* = 0.001), had shorter telomeres than children from the U.S.A. Though the paternal age (in years) did not differ between the countries (Analysis of variance (ANOVA) *P* = 0.102) as shown in Supplementary Table S3. Paternal age (*P* = 0.019) and the female sex of the child (*P* = 0.069; although not significant) were positively associated with telomere length. FDR status, presence of GDM in the mother, and maternal age did not have a significant impact on estimated telomere length (Table 2).

**Table 2:** Association between telomere length and characteristics of the subjects (sex, high risk HLA-DR-DQ haplogenotypes, country, first-degree relative (FDR) with T1D, gestational diabetes in the mother and parental age).

Parameter	Regression coefficient	Standard error	*P*-value
Child’s age (years)	− 0.029	0.0097	**0.003**
Sex			
Female	0.083	0.0458	0.069
High risk HLA-DR-DQ haplogenotypes			
HLA-DR3/3 or HLA-DR3/9	Reference	Reference	Reference
HLA-DR3/4	0.105	0.0647	0.106
HLA-DR4/4 or HLA-DR4/X	0.179	0.0675	**0.008**
Country			
U.S.A.	Reference	Reference	Reference
Finland	− 0.137	0.0671	**0.041**
Germany	− 0.100	0.1279	0.432
Sweden	− 0.191	0.0548	**0.001**
FDR with T1D			
General population	Reference	Reference	Reference
Father	0.109	0.0771	0.157
Mother	− 0.044	0.0985	0.653
Sibling	− 0.054	0.1203	0.653
Gestational diabetes in the mother	0.094	0.0943	0.317
Parental age			
Maternal age (years)	0.002	0.0064	0.792
Paternal age (years)	0.013	0.0055	**0.019**

Telomere length data were available on a total of 1119 TEDDY children. The age at sample collection and the indicator of the long-distance protocol sample collection (yes vs. no) were also included in the model.

### Association between telomere length and the risk of any IA, mIAA-first, GADA-first and T1D

Estimated telomere length was not significantly different with respect to any IA (OR = 0.91, 95% CI 0.75–1.12, *P* = 0.377), mIAA-first (OR = 1.23, 95% CI 0.87–1.73, *P* = 0.248), GADA-first (OR = 0.83, 95% CI 0.60–1.14, *P* = 0.248) or T1D (OR = 1.03, 95% CI 0.75–1.42, *P* = 0.861) (Table 3). There was a significantly increased risk for children with HLA-DR3/4 (OR = 1.85, 95% CI 1.23–2.78, *P* = 0.003) to develop IA regardless of type of islet autoantibody (any IA) as compared with those with HLA-DR3/3 or HLA-DR3/9 haplogenotypes. The risk to develop mIAA as the first autoantibody was significantly increased for children with HLA-DR3/4 (OR = 3.73, 95% CI 1.70–8.17, *P* = 0.001) and HLA-DR4/4 or HLA-DR4/X (OR = 2.59, 95% CI 1.16–5.78, *P* = 0.020) as compared to those with HLA-DR3/3 or HLA-DR3/9. The risk to develop GADA as the first autoantibody was not associated with any of the HLA-DR-DQ haplogenotypes (Table 3).

**Table 3:** Association between age-adjusted telomere length in subjects by the status of islet autoimmunity (IA, GADA-first or mIAA-first) and T1D in relation to HLA haplogenotypes.

HLA status	Odds ratio (95% CI)	*P*-value
**Any IA (n = 389 NCC pairs)**		
Age-adjusted telomere length	0.91 (0.75–1.12)	0.377
HLA-DR3/3 or HLA-DR3/9	Reference	Reference
HLA-DR3/4	**1.85 (1.23**–**2.78)**	**0.003**
HLA-DR4/4 or HLA-DR4/X	1.30 (0.84–2.02)	0.236
**mIAA-first (n = 159 NCC pairs)**		
Age-adjusted telomere length	1.23 (0.87–1.73)	0.248
HLA-DR3/3 or HLA-DR3/9	Reference	Reference
HLA-DR3/4	**3.73 (1.70**–**8.17)**	**0.001**
HLA-DR4/4 or HLA-DR4/X	**2.59 (1.16**–**5.78)**	**0.020**
**GADA-first (n = 158 NCC pairs)**		
Age-adjusted telomere length	0.83 (0.60–1.14)	0.248
HLA-DR3/3 or HLA-DR3/9	Reference	Reference
HLA-DR3/4	0.91 (0.51–1.63)	0.762
HLA-DR4/4 or HLA-DR4/X	0.69 (0.36–1.33)	0.266
**T1D (n = 118 NCC pairs)**		
Age-adjusted telomere length	1.03 (0.75–1.42)	0.861
HLA-DR3/3 or HLA-DR3/9 (Reference)	Reference	Reference
HLA-DR3/4	2.22 (0.97–5.10)	0.060
HLA-DR4/4 or HLA-DR4/X	1.63 (0.71–3.73)	0.249

Complete data were available on 389 nested case–control (NCC) pairs for IA and on 118 matched pairs for T1D.

Telomere length was adjusted for age when the sample was drawn.

Significant values are in [bold].

### SNPs associated with telomere length and the risk of any IA, mIAA-first, GADA-first and T1D

We selected 236 SNPs previously reported to be associated with telomere length (see Material and methods). A total of 31 SNPs in 13 regions were nominally associated with risk of developing any IA, mIAA-first, GADA-first or T1D; however, none of these associations withstood correction for multiple hypothesis testing (Supplementary Table S4 through S7). Six SNPs had nominally significant associations to more than one endpoint. rs11082257, an intronic SNP in *PIK3C3* on chromosome 18q12.3, was associated with decreased risk for any IA (HR = 0.88, 95% CI 0.78–0.99, *P* = 0.031), mIAA-first (HR = 0.76, 95% CI 0.62–0.93, *P* = 0.008) and T1D (HR = 0.79, 95% CI 0.65–0.97, *P* = 0.022). rs3017077 in *MRE11* on chromosome 11q21 was associated with decreased risk for any IA (HR = 0.90, 95% CI 0.81–1.00, *P* = 0.044) and mIAA-first (HR = 0.83, 95% CI 0.69–0.99, *P* = 0.038). rs75245322, an intronic SNP in *RAD50* on chromosome 5q31.1, was associated with increased risk for any IA (HR = 1.21, 95% CI 1.02–1.44, *P* = 0.030) and T1D (HR = 1.36, 95% CI 1.05–1.76, *P* = 0.018). rs7167216, a missense SNP in *BLM* on chromosome 15q26.1, was associated with increased risk for GADA-first (HR = 1.32, 95% CI 1.04–1.67, *P* = 0.022) and T1D (HR = 1.30, 95% CI 1.02–1.66, *P* = 0.038). rs1801516, a missense SNP in *ATM* on chromosome 11q22.3, was associated with increased risk for any IA (HR = 1.14, 95% CI 1.00–1.30, *P* = 0.045) and mIAA-first (HR = 1.24, 95% CI 1.01–1.53, *P* = 0.041). rs4353905 on chromosome 4q25 was associated with increased risk for any IA (HR = 1.15, 95% CI 1.01–1.31, *P* = 0.032) and mIAA-first (HR = 1.32, 95% CI 1.08–1.62, *P* = 0.006). All of the SNPs were located on the q-arms (long arms).

## Discussion

The key finding in this study was that children from high incidence countries for T1D i.e., Finland and especially from Sweden had shorter telomeres in white blood cells as compared to children from other TEDDY sites (Germany and three sites in the U.S.A.). The age when the blood sample was drawn had a significant negative correlation with telomere length, consistent with previous reports^41^. Interestingly, children that carried HLA-DR4/4 or HLA-DR4/X had significantly longer telomeres compared to children with HLA-DR3/3 or HLA-DR3/9 haplogenotypes. Also, paternal age was significantly correlated with telomere length. Children with HLA-DR3/4 had a significantly increased risk to develop any IA as compared to children with HLA-DR3/3 or HLA-DR3/9 haplogenotypes. Moreover, there were significantly increased risks for children with HLA-DR3/4 and HLA-DR4/4 or HLA-DR4/X to develop mIAA as the first autoantibody as compared to those with HLA-DR3/3 or HLA-DR3/9 haplogenotypes. In contrast, GADA as the first autoantibody was not associated with any of the HLA-DR-DQ haplogenotypes.

To our knowledge, there has been no reports on the risk for IA, mIAA-first or GADA-first in relation to telomere length. The estimations of the telomeres were highly correlated between Computel, Telseq and Motif_counter with R^2^ values ranging from 0.85 to 0.94 (Supplemental Fig. 1). The finding of similar telomere lengths in young boys and girls confirms a previous study of telomere length taken at birth^6^. There has been a previous report on shorter telomere length in children with T1D^16^, a finding that was not reproduced here. This discrepancy could be explained by the older age of the participants in the previous report (17–48 years), compared with the TEDDY study in which the oldest children with T1D were 14.16 years. We could not reproduce the findings of shorter telomeres in children born to mothers with GDM, although this could be due to the older age (9–16 years) in the earlier study^13^. In addition, we detected a significant effect of paternal age but not maternal age on the length of telomeres^5^. The SNPs that had nominally significant association to our primary and secondary outcomes—any IA, mIAA-first, GADA-first or T1D—were mainly from genes coding for proteins involved in double DNA strand repair and maintenance of telomeres (*MRE11* and *RAD50*)^42^, DNA replication and repair (*BLM*)^43^, genome stability (*ATM*)^44^ or degradative endocytic trafficking (*PIK3C3*)^45^. The *BLM* gene has gene ontology including nucleic acid binding and ATPase activity also involved in DNA replication and repair.

The major strength of the present study is the use of both IA and T1D as endpoints. Additional strengths of this study are the inclusion of unrelated subjects from both the general population and FDRs without islet autoantibodies or T1D from the same countries in the same age ranges as the subjects with outcome events. One limitation of the present study is the relatively low number of subjects with IA, particularly those with GADA-first or with T1D, raising concerns for statistical power of the association analysis. Nevertheless, the screening of more than 400,000 newborns in four different countries to identify those with an increased genetic risk for T1D, defined by high-risk HLA-DR-DQ haplogenotypes, followed with blood draws every three months for the first four years and every six months thereafter is a unique effort. The proportional hazard ratio identifies risk for time-to-event in this longitudinal study, thereby increasing the power compared with cross-sectional case–control study design.

We concluded that genes in the HLA-region particularly HLA-DR3/4 and HLA-DR4/4, HLA-DR4/X remain the most important genetic risk factors for IA, the first step towards T1D. Children with the high-risk genotypes for T1D from Finland and Sweden had shorter telomeres and telomere length is associated with HLA-DR4/4 or HLA-DR4/X haplogenotype as compared to HLA-DR3/3 or HLA-DR3/9. Moreover, paternal age was positively associated with telomere length.

## Supplementary Information

Supplementary Information 1.

Supplementary Information 2.

## Data Availability

The datasets generated and analyzed during the current study will be made available in the NIDDK Central Repository at https://repository.niddk.nih.gov/studies/teddy/. The TEDDY Whole Genome Sequencing (WGS) data that support the findings of this study have been deposited in NCBI’s database of Genotypes and Phenotypes (dbGaP) with the primary accession code phs001442.v3.p2.

## Code availability

The bioinformatics analysis code is available on the GitHub page: https://github.com/USF-HII/pub-teddy-human-wgs-telomere.
