# Supplementary material for: Telomere length is not a main factor for the development of islet autoimmunity and type 1 diabetes in the TEDDY study
Source: Sci Rep. 2022 Mar 16;12:4516. doi: 10.1038/s41598-022-08058-7 (PMC8927592; doi:10.1038/s41598-022-08058-7)
Supplement: Supplementary file 1 — Supplementary Information 1. [file 41598_2022_8058_MOESM1_ESM.pdf]

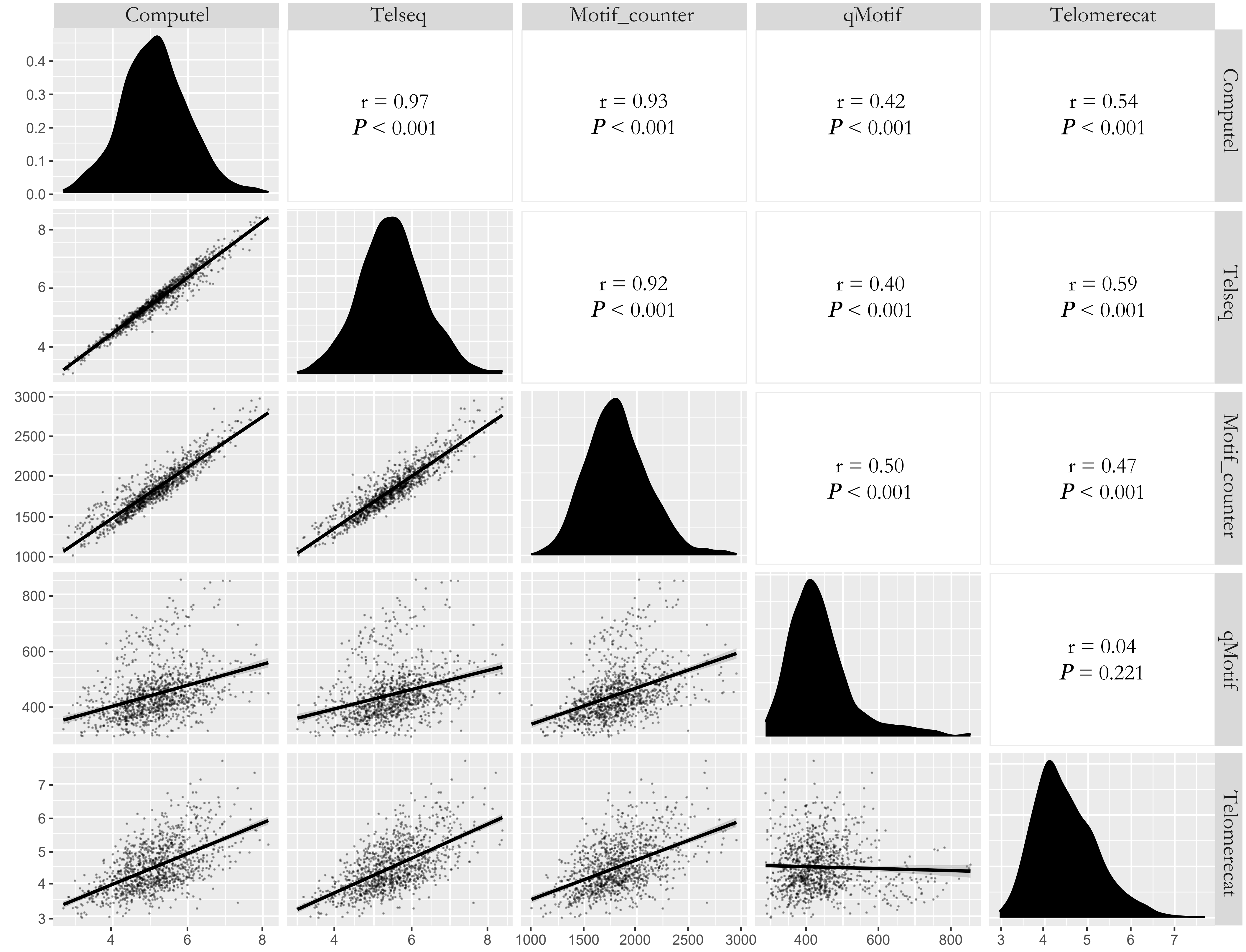

Supplementary Figure 1: Illustration of the correlation between five different tools to estimate telomere length from the whole-genome sequence data. The tools were as follows: (1) Computel (in kb) (2) Telseq (in kb) (2) Motif_counter (telomere reads per 1x genome coverage) (3) qMotif (telomere reads per 1x genome coverage) (4) Telomerecat (in kb).
