# Supplementary material for: Telomere length is not a main factor for the development of islet autoimmunity and type 1 diabetes in the TEDDY study
Source: Sci Rep. 2022 Mar 16;12:4516. doi: 10.1038/s41598-022-08058-7 (PMC8927592; doi:10.1038/s41598-022-08058-7)
Supplement: Supplementary file 2 — Supplementary Information 2. [file 41598_2022_8058_MOESM2_ESM.docx]

**Supplementary methods**

Estimation of the telomere content from the whole-genome sequencing (WGS) data was performed using five different tools detailed below: Computel ^1^, Telseq ^2^, Telomere Computational Analysis Tool (Telomerecat) ^3^, qMotif (<https://sourceforge.net/p/adamajava/wiki/qMotif>) and Motif_counter (<https://sourceforge.net/projects/motifcounter/>). Computel, Telseq and Telomerecat provide estimation of the average telomere length while qMotif and Motif_counter provide estimation of the number telomere motifs based on the WGS data. The optimal criteria for each tool was selected based on the recommendation from ^4^.

Estimation of the telomeres from the WGS data was implemented using Snakemake workflow management system. The code is available on GitHub page: <https://github.com/USF-HII/pub-teddy-human-wgs-telomere>.

**(1) Computel**

Version #: 1.2

Motif: min.seed 54

Command line:

computel.sh -1 *input.fastq_1* -2 *input.fastq_2* -pattern TTAGGG -nchr 23 -minseed 54 -o *output_path*

**(2) Telseq**

Version #: 0.0.1

Motif: 12 non-consecutive TTAGGG

Reference Genome: Homo sapiens (human) genome assembly GRCh38 (GRCh38DH)

Command line:

telseq -u -r 150 -k 12 *input_file.bam* > *output_file.txt*

**(3) Telomere Computational Analysis Tool (Telomerecat)**

Version #: 3.4.0

Motif: 12 non-consecutive TTAGGG

Reference Genome: Homo sapiens (human) genome assembly GRCh38 (GRCh38DH)

Command line:

telomerecat bam2telbam -v 2 *input_file.bam* > *output_file.bam*

telomerecat telbam2length -v 2 -e *input_file.bam* > *output_file.txt*

**(4) qMotif**

Version #: 1.2

Motif: 9 consecutive TTAGGG

Reference Genome: Homo sapiens (human) genome assembly GRCh37 (GRCh37d5)

Command line:

java -jar qmotif-1.2.jar -n 4 --bam *input.bam* --bai *input.bai* --log *log_file.log* --loglevel INFO -ini config_file.ini -o *output.xml* -o *output.bam*

Additional files:

*config_file.ini*

| [PARAMS]  stage1_motif_string=TTAGGGTTAGGGTTAGGGTTAGGGTTAGGGTTAGGGTTAGGGTTAGGGTTAGGG  stage2_motif_regex=(TTAGGG\|ATAGGG\|CTAGGG\|GTAGGG\|TAAGGG\|TCAGGG\|TGAGGG\|TTCGGG\|TTGGGG\|TTTGGG\|TTAAGG\|TTACGG\|TTATGG\|TTAGAG\|TTAGCG\|TTAGTG\|TTAGGA\|TTAGGC\|TTAGGT\|CCCTAA\|ACCTAA\|GCCTAA\|TCCTAA\|CACTAA\|CGCTAA\|CTCTAA\|CCATAA\|CCGTAA\|CCTTAA\|CCCAAA\|CCCCAA\|CCCGAA\|CCCTCA\|CCCTGA\|CCCTTA\|CCCTAC\|CCCTAG\|CCCTAT)  revcomp=true  window_size=10000  cutoff_size=5  includes_only=true  [INCLUDES]  ; name, regions (sequence:start-stop)  1p 1:10001-12464  1q 1:249237907-249240620  2p 2:10001-12592  2q 2:243187373-243189372  2xA 2:243150480-243154648  3p 3:60001-62000  3q 3:197960430-197962429  3xB 3:197897576-197903397  4p 4:10001-12193  4q 4:191041613-191044275  5p 5:10001-13806  5q 5:180903260-180905259  6p 6:60001-62000  6q 6:171053067-171055066  7p 7:10001-12238  7q 7:159126558-159128662  8p 8:10001-12000  8q 8:146302022-146304021  9p 9:10001-12359  9q 9:141151431-141153430  10p 10:60001-62000  10q 10:135522469-135524746  11p 11:60001-62000  11q 11:134944458-134946515  12p 12:60001-62000  12q 12:133839458-133841894  12xC 12:93158-97735  13p 13:19020001-19022000  13q 13:115107878-115109877  14p 14:19020001-19022000  14q 14:107287540-107289539  15p 15:20000001-20002000  15q 15:102518969-102521391  16p 16:60001-62033  16q 16:90292753-90294752  17p 17:1-2000  17q 17:81193211-81195210  18p 18:10001-12621  18q 18:78014226-78017247  19p 19:60001-62000  19q 19:59116822-59118982  20p 20:60001-62000  20q 20:62963520-62965519  21p 21:9411194-9413193  21q 21:48117788-48119894  22p 22:16050001-16052000  22q 22:51242566-51244565  Xp X:60001-62033  Xq X:155257733-155260559  Yp Y:10001-12033  Yq Y:59360739-59363565 |
| --- |

**(5) Motif_counter**

Version #: 1.0

Motif: 9 consecutive TTAGGG

Reference Genome: Homo sapiens (human) genome assembly GRCh38 (GRCh38DH)

Command line:

echo -e "TTAGGG\n9" | bash motif_counter.sh -p -v -i *input.bam* -s -q 0 -Q 0 -o *output_file.txt*

**Supplementary Table S1**: High-risk HLA haplogenotypes constituting the criteria for eligibility for children from the general population (GP) and first-degree relative (FDR). Haplopenotypes A, B, C and D confer eligibility for children from GP, but exclude DRB1*04:03. Haplogenotypes E, F, G, H, I and J confer eligibility for children of an FDR of a T1D patient.

| **Code in TEDDY** | **Haplotype genotypes** | **Abbreviation** | **General population** |
| --- | --- | --- | --- |
| A | DRB1*03-DQA1*05:01-DQB1*02:01 / DRB1*04-DQA1*03-DQB1*03:02^§^ | DR3/4 | Yes |
| B | DRB1*04-DQA1*03-DQB1*03:02^§^ / DRB1*04-DQA1*03-DQB1*03:02^§^ | DR4/4 | Yes |
| C | DRB1*04-DQA1*03-DQB1*03:02^§^ / DRB1*08-DQA1*04:01-DQB1*04:02 | DR4/8 | Yes |
| D | DRB1*03-DQA1*05:01-DQB1*02:01 / DRB1*03-DQA1*05:01-DQB1*02:01 | DR3/3 | Yes |
| E | DRB1*04-DQA1*03-DQB1*03:02^§^ / DRB1*04-DQA1*03-DQB1*02:02 | DR4/4b | No |
| F | DRB1*04-DQA1*03-DQB1*03:02^§^ / DRB1*01-DQA1*01:01-DQB1*05:01 | DR4/1 | No |
| G | DRB1*04-DQA1*03-DQB1*03:02^§^ / DRB1*13-DQA1*01:02-DQB1*06:04 | DR4/13 | No |
| H | DRB1*04-DQA1*03-DQB1*03:02^§^ / DRB1*04-DQA1*03-DQB1*03:04 | DR4/4c | No |
| I | DRB1*04-DQA1*03-DQB1*03:02^§^ / DRB1*09-DQA1*03-DQB1*03:03 | DR4/9 | No |
| J | DRB1*03-DQA1*05:01-DQB1*02:01 / DRB1*09-DQA1*03-DQB1*03:03 | DR3/9 | No |

^§^Where DQB1*03:02 is noted, either allele or DQB1*03:04 is allowed.

**Supplementary Table S2**: Characteristics of subjects by the status of islet autoimmunity (any IA, GADA-first or mIAA-first) and T1D included in the SNP association analysis.

|  | **Any IA** | **Controls** | **mIAA-first** | **GADA-first** | **T1D** |
| --- | --- | --- | --- | --- | --- |
| ***Number of subjects (n)*** | 813 | 7,280 | 299 | 365 | 339 |
| ***Age at the first endpoint (months)*** |  |  |  |  |  |
| Median | 40 | 125 | 25 | 58 | 83 |
| Interquartile range | 19.5 - 86.1 | 41.4 - 148.0 | 12.5 - 55.2 | 28.3 - 98.3 | 47.1 - 117.0 |
| ***Sex (%)*** |  |  |  |  |  |
| Female | 366 (45.0) | 3,600 (49.5) | 132 (44.1) | 170 (46.6) | 155 (45.7) |
| Male | 447 (55.0) | 3,680 (50.5) | 167 (55.9) | 195 (53.4) | 184 (54.3) |
| ***High risk HLA-DR-DQ haplogenotypes (%)*** |  |  |  |  |  |
| HLA-DR3/3 or HLA-DR3/9 | 119 (14.6) | 1,599 (22.0) | 30 (10.0) | 81 (22.2) | 30 (8.8) |
| HLA-DR3/4 | 393 (48.3) | 2,755 (37.8) | 138 (46.2) | 180 (49.3) | 192 (56.6) |
| HLA-DR4/4 or HLA-DR4/X | 301 (37.0) | 2,926 (40.2) | 131 (43.8) | 104 (28.5) | 117 (34.5) |
| ***Country (%)*** |  |  |  |  |  |
| Finland | 195 (24.0) | 1,578 (21.7) | 92 (30.8) | 67 (18.4) | 92 (27.1) |
| Germany | 53 (6.5) | 490 (6.7) | 19 (6.4) | 16 (4.4) | 31 (9.1) |
| Sweden | 281 (34.6) | 2,121 (29.1) | 92 (30.8) | 141 (38.6) | 92 (27.1) |
| U.S.A. | 284 (34.9) | 3,091 (42.5) | 96 (32.1) | 141 (38.6) | 124 (36.6) |

**Supplementary Table S3**: The paternal age descriptive statistics across the four countries.

| **Country** | **Mean**  **(years)** | **SD**  **(years)** | **Median**  **(years)** | **Lower Quartile**  **(years)** | **Upper Quartile**  **(years)** |
| --- | --- | --- | --- | --- | --- |
| **Finland (n = 231)** | 32.9 | 5.6 | 33.0 | 29.0 | 36.0 |
| **Germany (n = 53)** | 34.9 | 4.7 | 35.0 | 32.0 | 38.0 |
| **Sweden (n = 462)** | 33.6 | 5.6 | 33.0 | 30.0 | 37.0 |
| **U.S.A. (n = 373)** | 33.5 | 5.9 | 33.0 | 29.0 | 37.0 |

**Supplementary Table S4**: Assessment of 236 SNPs associated with the telomere length with risk for any islet autoimmunity (IA). Risk was estimated as Hazard ratios and 95% confidence intervals.

| **SNP IDs** | **Chromosome** | **Position** | **Gene** | **Minor allele** | **Major allele** | **MAF in cases** | **MAF in controls** | **HR (95% CI)** | ***P*-value** | **FDR adjusted *P*-value** |
| --- | --- | --- | --- | --- | --- | --- | --- | --- | --- | --- |
| rs628728 | 11 | 94250379 | *C11orf97* | A | G | 0.39 | 0.42 | 0.88 (0.80-0.98) | 0.016 | 0.699 |
| rs75245322 | 5 | 131903026 | *RAD50* | C | A | 0.09 | 0.08 | 1.21 (1.02-1.44) | 0.030 | 0.699 |
| rs11082257 | 18 | 39570683 | *PIK3C3* | G | A | 0.22 | 0.25 | 0.88 (0.78-0.99) | 0.031 | 0.699 |
| rs4353905 | 4 | 110745938 | - | G | A | 0.17 | 0.16 | 1.15 (1.01-1.31) | 0.032 | 0.699 |
| rs6601530 | 8 | 10671272 | *PINX1* | G | A | 0.45 | 0.47 | 0.90 (0.81-0.99) | 0.038 | 0.699 |
| rs654718 | 11 | 94190115 | *MRE11* | G | A | 0.32 | 0.35 | 0.90 (0.81-1.00) | 0.043 | 0.699 |
| rs3017077 | 11 | 94175249 | *MRE11* | A | G | 0.32 | 0.34 | 0.90 (0.81-1.00) | 0.044 | 0.699 |
| rs1801516 | 11 | 108175462 | *ATM* | A | G | 0.17 | 0.15 | 1.14 (1.00-1.30) | 0.045 | 0.699 |
| rs733396 | 8 | 10621981 | *LOC102723313* | A | G | 0.47 | 0.44 | 1.11 (1.00-1.22) | 0.048 | 0.699 |
| rs1941526 | 18 | 39652939 | *PIK3C3* | A | G | 0.22 | 0.25 | 0.89 (0.79-1.00) | 0.053 | 0.699 |
| rs4463363 | 7 | 124513770 | *POT1* | A | G | 0.40 | 0.43 | 0.91 (0.82-1.00) | 0.059 | 0.699 |
| rs7167216 | 15 | 91354521 | *BLM* | A | G | 0.09 | 0.08 | 1.18 (0.99-1.40) | 0.059 | 0.699 |
| rs13265363 | 8 | 9422926 | *TNKS* | C | A | 0.12 | 0.14 | 0.87 (0.75-1.01) | 0.065 | 0.699 |
| rs622961 | 11 | 94154902 | *MRE11* | A | G | 0.42 | 0.44 | 0.91 (0.82-1.01) | 0.065 | 0.699 |
| rs16944894 | 15 | 91363303 | - | G | A | 0.21 | 0.19 | 1.12 (0.99-1.27) | 0.065 | 0.699 |
| rs2228041 | 14 | 20852267 | *TEP1* | A | G | 0.07 | 0.06 | 1.20 (0.99-1.46) | 0.067 | 0.699 |
| rs962863 | 7 | 124558339 | *POT1* | A | G | 0.40 | 0.43 | 0.92 (0.83-1.01) | 0.076 | 0.699 |
| rs609261 | 11 | 108158134 | *ATM* | A | G | 0.42 | 0.44 | 0.91 (0.83-1.01) | 0.078 | 0.699 |
| rs599558 | 11 | 108177538 | *ATM* | G | A | 0.42 | 0.44 | 0.91 (0.83-1.01) | 0.078 | 0.699 |
| rs4585 | 11 | 108239628 | *ATM*, *C11orf65* | C | A | 0.42 | 0.44 | 0.91 (0.83-1.01) | 0.080 | 0.699 |
| rs9282580 | 1 | 226594372 | *PARP1* | A | G | 0.04 | 0.05 | 0.80 (0.63-1.03) | 0.081 | 0.699 |
| rs619972 | 11 | 108169619 | *ATM* | G | A | 0.43 | 0.44 | 0.91 (0.83-1.01) | 0.082 | 0.699 |
| rs645485 | 11 | 108168863 | *ATM* | A | G | 0.42 | 0.44 | 0.92 (0.83-1.01) | 0.085 | 0.699 |
| rs600931 | 11 | 108117335 | *ATM* | G | A | 0.42 | 0.44 | 0.92 (0.83-1.01) | 0.086 | 0.699 |
| rs3755132 | 2 | 15729820 | - | C | A | 0.18 | 0.16 | 1.12 (0.98-1.28) | 0.092 | 0.699 |
| rs7015700 | 8 | 9527707 | *TNKS* | A | G | 0.18 | 0.20 | 0.90 (0.79-1.02) | 0.093 | 0.699 |
| rs663530 | 11 | 94157114 | *MRE11* | A | G | 0.24 | 0.26 | 0.91 (0.81-1.02) | 0.093 | 0.699 |
| rs228591 | 11 | 108097333 | *ATM* | A | G | 0.42 | 0.44 | 0.92 (0.83-1.02) | 0.095 | 0.699 |
| rs2853672 | 5 | 1292983 | *TERT* | A | C | 0.48 | 0.50 | 1.09 (0.99-1.20) | 0.095 | 0.699 |
| rs11160462 | 14 | 20868751 | *TEP1* | A | G | 0.35 | 0.37 | 0.92 (0.83-1.02) | 0.099 | 0.699 |
| rs3755133 | 2 | 15731583 | - | A | G | 0.18 | 0.17 | 1.12 (0.98-1.28) | 0.100 | 0.699 |
| rs11998382 | 8 | 10659517 | *PINX1* | G | A | 0.24 | 0.22 | 1.10 (0.98-1.24) | 0.104 | 0.699 |
| rs2508678 | 11 | 94149349 | *MRE11* | A | G | 0.35 | 0.37 | 0.92 (0.83-1.02) | 0.109 | 0.699 |
| rs11930711 | 4 | 110734498 | - | A | G | 0.10 | 0.09 | 1.14 (0.97-1.34) | 0.114 | 0.699 |
| rs12567614 | 1 | 226544420 | - | A | G | 0.47 | 0.46 | 1.09 (0.98-1.20) | 0.115 | 0.699 |
| rs976016 | 2 | 15772865 | - | G | A | 0.19 | 0.17 | 1.11 (0.97-1.26) | 0.121 | 0.699 |
| rs1944967 | 18 | 39531139 | - | G | A | 0.36 | 0.39 | 0.92 (0.83-1.02) | 0.123 | 0.699 |
| rs11250076 | 8 | 10647823 | *PINX1* | A | G | 0.45 | 0.47 | 0.93 (0.84-1.02) | 0.123 | 0.699 |
| rs12706627 | 7 | 124550133 | *POT1* | G | A | 0.33 | 0.31 | 1.09 (0.98-1.21) | 0.124 | 0.699 |
| rs476137 | 11 | 94213905 | *MRE11* | A | C | 0.41 | 0.43 | 0.92 (0.84-1.02) | 0.125 | 0.699 |
| rs9939870 | 16 | 69396585 | *TERF2* | A | G | 0.26 | 0.25 | 1.09 (0.98-1.21) | 0.129 | 0.699 |
| rs2104978 | 14 | 20837033 | *TEP1* | G | A | 0.07 | 0.06 | 1.16 (0.96-1.40) | 0.133 | 0.699 |
| rs623860 | 11 | 108106782 | *ATM* | G | A | 0.41 | 0.43 | 0.93 (0.84-1.02) | 0.134 | 0.699 |
| rs4244612 | 8 | 145741702 | *RECQL4* | C | G | 0.38 | 0.41 | 0.93 (0.84-1.02) | 0.135 | 0.699 |
| rs17772583 | 5 | 131953510 | *RAD50* | G | A | 0.25 | 0.23 | 1.09 (0.97-1.23) | 0.135 | 0.699 |
| rs664143 | 11 | 108225661 | *ATM*, *C11orf65* | A | G | 0.41 | 0.43 | 0.93 (0.84-1.03) | 0.144 | 0.699 |
| rs4796033 | 17 | 33433487 | *RAD51D*, *RAD51L3-RFFL* | A | G | 0.14 | 0.15 | 0.90 (0.78-1.04) | 0.149 | 0.699 |
| rs12679892 | 8 | 9445429 | *TNKS* | G | A | 0.33 | 0.31 | 1.08 (0.97-1.20) | 0.150 | 0.699 |
| rs17772565 | 5 | 131952405 | *RAD50* | A | G | 0.05 | 0.06 | 0.84 (0.67-1.06) | 0.150 | 0.699 |
| rs740059 | 12 | 4671490 | *LOC113939935*, *DYRK4* | G | A | 0.50 | 0.48 | 1.07 (0.98-1.18) | 0.151 | 0.699 |
| rs12142240 | 1 | 46747301 | *LRRC41* | G | A | 0.33 | 0.30 | 1.08 (0.97-1.20) | 0.151 | 0.699 |
| rs11250077 | 8 | 10656437 | *PINX1* | A | G | 0.41 | 0.43 | 0.93 (0.84-1.03) | 0.162 | 0.712 |
| rs2736100 | 5 | 1286516 | *TERT* | C | A | 0.48 | 0.50 | 1.07 (0.97-1.18) | 0.164 | 0.712 |
| rs7159947 | 14 | 20809491 | - | G | A | 0.38 | 0.36 | 1.07 (0.97-1.18) | 0.165 | 0.712 |
| rs1871892 | 17 | 33448818 | *FNDC8* | A | G | 0.31 | 0.32 | 0.93 (0.83-1.03) | 0.168 | 0.712 |
| rs425538 | 11 | 108219339 | *ATM*, *C11orf65* | C | A | 0.41 | 0.43 | 0.93 (0.84-1.03) | 0.172 | 0.712 |
| rs7299185 | 12 | 4672394 | *DYRK4* | C | A | 0.24 | 0.25 | 0.93 (0.83-1.03) | 0.172 | 0.712 |
| rs13250838 | 8 | 9526345 | *TNKS* | A | G | 0.11 | 0.13 | 0.90 (0.77-1.05) | 0.176 | 0.716 |
| rs7175811 | 15 | 91331546 | *BLM* | A | G | 0.38 | 0.36 | 1.07 (0.97-1.19) | 0.188 | 0.739 |
| rs414634 | 15 | 91356253 | *BLM* | A | C | 0.27 | 0.28 | 0.93 (0.83-1.04) | 0.191 | 0.739 |
| rs13447720 | 11 | 94165326 | *MRE11* | G | A | 0.24 | 0.22 | 1.08 (0.96-1.21) | 0.191 | 0.739 |
| rs6470522 | 8 | 90954481 | *NBN* | A | G | 0.16 | 0.17 | 0.92 (0.80-1.05) | 0.199 | 0.745 |
| rs17774023 | 8 | 10626333 | *PINX1*, *LOC102723313* | G | A | 0.31 | 0.29 | 1.07 (0.96-1.19) | 0.199 | 0.745 |
| rs8022805 | 14 | 20846950 | *TEP1* | A | G | 0.06 | 0.05 | 1.14 (0.93-1.39) | 0.204 | 0.752 |
| rs7184015 | 15 | 91274575 | *BLM* | A | C | 0.27 | 0.28 | 0.93 (0.84-1.04) | 0.228 | 0.819 |
| rs2291207 | 7 | 124569382 | *POT1* | G | A | 0.33 | 0.31 | 1.07 (0.96-1.18) | 0.229 | 0.819 |
| rs1805812 | 8 | 90965053 | *NBN* | G | A | 0.09 | 0.09 | 0.90 (0.76-1.07) | 0.235 | 0.821 |
| rs17183344 | 15 | 91357488 | *BLM* | A | G | 0.19 | 0.18 | 1.08 (0.95-1.22) | 0.238 | 0.821 |
| rs3219125 | 1 | 226554951 | *PARP1* | G | A | 0.07 | 0.06 | 1.12 (0.93-1.37) | 0.240 | 0.821 |
| rs2227935 | 15 | 91326099 | *BLM* | A | G | 0.08 | 0.07 | 1.11 (0.92-1.33) | 0.266 | 0.847 |
| rs936656 | 17 | 33433820 | *RAD51D*, *RAD51L3-RFFL* | G | A | 0.45 | 0.47 | 0.94 (0.85-1.05) | 0.271 | 0.847 |
| rs1805414 | 1 | 226573364 | *PARP1* | G | A | 0.38 | 0.37 | 1.06 (0.96-1.18) | 0.271 | 0.847 |
| rs7170919 | 15 | 91314959 | *BLM* | A | G | 0.30 | 0.29 | 1.06 (0.95-1.18) | 0.278 | 0.847 |
| rs2736122 | 5 | 1257621 | *TERT* | A | G | 0.23 | 0.24 | 0.94 (0.84-1.05) | 0.284 | 0.847 |
| rs10090277 | 8 | 9450886 | *TNKS* | G | A | 0.07 | 0.08 | 0.90 (0.74-1.10) | 0.297 | 0.847 |
| rs3102095 | 8 | 73916745 | - | C | A | 0.30 | 0.32 | 0.95 (0.85-1.05) | 0.297 | 0.847 |
| rs1760897 | 14 | 20876253 | *TEP1* | G | A | 0.29 | 0.31 | 0.95 (0.85-1.05) | 0.297 | 0.847 |
| rs61835377 | 1 | 226545854 | - | A | G | 0.19 | 0.20 | 0.93 (0.82-1.06) | 0.298 | 0.847 |
| rs4660918 | 1 | 46731694 | *RAD54L* | G | A | 0.25 | 0.26 | 0.94 (0.84-1.06) | 0.299 | 0.847 |
| rs10099824 | 8 | 73940350 | *TERF1* | A | G | 0.47 | 0.45 | 1.05 (0.95-1.16) | 0.304 | 0.847 |
| rs4982038 | 14 | 20862542 | *TEP1* | A | G | 0.31 | 0.32 | 0.95 (0.85-1.05) | 0.306 | 0.847 |
| rs2227933 | 15 | 91337479 | *BLM* | A | G | 0.19 | 0.17 | 1.07 (0.94-1.21) | 0.319 | 0.847 |
| rs2242652 | 5 | 1280028 | *TERT* | A | G | 0.20 | 0.21 | 0.94 (0.84-1.06) | 0.323 | 0.847 |
| rs2072352 | 15 | 91334179 | *BLM* | A | G | 0.30 | 0.29 | 1.06 (0.95-1.17) | 0.329 | 0.847 |
| rs6984094 | 8 | 10645738 | *PINX1* | G | A | 0.11 | 0.12 | 0.93 (0.80-1.08) | 0.329 | 0.847 |
| rs6601524 | 8 | 10635222 | *PINX1* | A | C | 0.23 | 0.22 | 1.06 (0.94-1.20) | 0.331 | 0.847 |
| rs78733221 | 8 | 9476448 | *TNKS* | G | A | 0.07 | 0.08 | 0.91 (0.75-1.10) | 0.331 | 0.847 |
| rs13276086 | 8 | 9578982 | *TNKS* | A | C | 0.38 | 0.39 | 0.95 (0.86-1.05) | 0.333 | 0.847 |
| rs6668851 | 1 | 226545866 | - | C | A | 0.31 | 0.30 | 1.06 (0.95-1.18) | 0.333 | 0.847 |
| rs6011011 | 20 | 62299578 | *RTEL1*, *RTEL1-TNFRSF6B* | A | G | 0.07 | 0.08 | 0.91 (0.75-1.10) | 0.336 | 0.847 |
| rs61909598 | 12 | 4671930 | *DYRK4* | A | C | 0.13 | 0.12 | 1.07 (0.93-1.24) | 0.337 | 0.847 |
| rs12334407 | 8 | 73953688 | *TERF1* | A | C | 0.30 | 0.32 | 0.95 (0.85-1.06) | 0.341 | 0.847 |
| rs6668722 | 1 | 226545776 | - | G | A | 0.11 | 0.10 | 1.08 (0.92-1.27) | 0.343 | 0.847 |
| rs11249930 | 8 | 9472445 | *TNKS* | G | A | 0.21 | 0.19 | 1.06 (0.94-1.20) | 0.348 | 0.847 |
| rs12125573 | 1 | 46734792 | *RAD54L* | G | A | 0.29 | 0.30 | 0.95 (0.85-1.06) | 0.348 | 0.847 |
| rs12638862 | 3 | 169477506 | - | G | A | 0.26 | 0.28 | 0.95 (0.84-1.06) | 0.348 | 0.847 |
| rs10101132 | 8 | 9616553 | *TNKS* | A | C | 0.31 | 0.32 | 0.95 (0.86-1.05) | 0.350 | 0.847 |
| rs7302664 | 12 | 4664821 | *RAD51AP1* | A | G | 0.11 | 0.10 | 1.08 (0.92-1.25) | 0.360 | 0.847 |
| rs2293464 | 1 | 226576296 | *PARP1* | A | G | 0.17 | 0.16 | 1.06 (0.93-1.21) | 0.368 | 0.847 |
| rs59672299 | 1 | 226597593 | - | G | A | 0.17 | 0.16 | 1.06 (0.93-1.21) | 0.369 | 0.847 |
| rs2228035 | 14 | 20871973 | *TEP1* | G | A | 0.04 | 0.05 | 0.90 (0.71-1.14) | 0.376 | 0.847 |
| rs12680047 | 8 | 128758861 | - | G | A | 0.39 | 0.39 | 0.96 (0.86-1.06) | 0.376 | 0.847 |
| rs1000033 | 1 | 226580387 | *PARP1* | C | A | 0.17 | 0.16 | 1.06 (0.93-1.21) | 0.377 | 0.847 |
| rs2853676 | 5 | 1288547 | *TERT* | A | G | 0.24 | 0.25 | 0.95 (0.85-1.07) | 0.377 | 0.847 |
| rs11211262 | 1 | 46721155 | *RAD54L* | A | G | 0.39 | 0.40 | 0.96 (0.86-1.06) | 0.377 | 0.847 |
| rs7838341 | 8 | 10661734 | *PINX1* | G | A | 0.23 | 0.23 | 1.05 (0.94-1.19) | 0.381 | 0.848 |
| rs16943176 | 17 | 56769887 | *RAD51C* | A | G | 0.23 | 0.22 | 1.05 (0.94-1.18) | 0.393 | 0.859 |
| rs3219090 | 1 | 226564691 | *PARP1* | A | G | 0.37 | 0.37 | 1.05 (0.94-1.16) | 0.393 | 0.859 |
| rs604845 | 11 | 94182689 | *MRE11* | A | G | 0.34 | 0.33 | 1.05 (0.94-1.17) | 0.397 | 0.859 |
| rs1800392 | 8 | 30973957 | *WRN* | A | C | 0.43 | 0.44 | 0.96 (0.87-1.06) | 0.404 | 0.859 |
| rs4427176 | 8 | 9533399 | *TNKS* | A | C | 0.20 | 0.19 | 1.05 (0.93-1.19) | 0.414 | 0.859 |
| rs7826180 | 8 | 10627844 | *PINX1*, *LOC102723313* | A | G | 0.23 | 0.22 | 1.05 (0.93-1.18) | 0.417 | 0.859 |
| rs1800391 | 8 | 30938704 | *WRN* | A | G | 0.07 | 0.08 | 0.92 (0.76-1.12) | 0.421 | 0.859 |
| rs3848668 | 20 | 62293272 | *RTEL1*, *RTEL1-TNFRSF6B* | G | A | 0.07 | 0.07 | 0.92 (0.76-1.12) | 0.427 | 0.859 |
| rs2110159 | 12 | 21649160 | *RECQL* | G | A | 0.49 | 0.50 | 0.96 (0.87-1.06) | 0.433 | 0.859 |
| rs114939615 | 1 | 226591587 | *PARP1* | A | G | 0.07 | 0.07 | 1.08 (0.89-1.31) | 0.434 | 0.859 |
| rs9822885 | 3 | 169486144 | *ACTRT3* | G | A | 0.28 | 0.29 | 0.96 (0.85-1.07) | 0.439 | 0.859 |
| rs1713456 | 14 | 20850093 | *TEP1* | A | G | 0.20 | 0.19 | 1.05 (0.93-1.18) | 0.444 | 0.859 |
| rs3755130 | 2 | 15729583 | - | A | G | 0.43 | 0.42 | 1.04 (0.94-1.14) | 0.445 | 0.859 |
| rs538800 | 11 | 94239567 | *MRE11* | A | G | 0.34 | 0.33 | 1.04 (0.94-1.16) | 0.452 | 0.859 |
| rs6601333 | 8 | 9427048 | *TNKS* | G | A | 0.26 | 0.25 | 1.04 (0.93-1.16) | 0.456 | 0.859 |
| rs709816 | 8 | 90967711 | *NBN* | G | A | 0.40 | 0.39 | 1.04 (0.94-1.15) | 0.460 | 0.859 |
| rs9956832 | 18 | 39564876 | *PIK3C3* | A | C | 0.14 | 0.13 | 1.06 (0.91-1.22) | 0.461 | 0.859 |
| rs1805793 | 8 | 90990091 | *NBN* | G | A | 0.40 | 0.39 | 1.04 (0.94-1.15) | 0.462 | 0.859 |
| rs12630450 | 3 | 169480204 | - | G | A | 0.28 | 0.30 | 0.96 (0.86-1.07) | 0.468 | 0.859 |
| rs10102170 | 8 | 9611470 | *TNKS* | A | G | 0.38 | 0.39 | 0.96 (0.87-1.07) | 0.472 | 0.859 |
| rs2945461 | 14 | 20863070 | *TEP1* | G | A | 0.09 | 0.10 | 0.94 (0.79-1.12) | 0.478 | 0.859 |
| rs33994795 | 8 | 9473429 | *TNKS* | G | A | 0.39 | 0.40 | 0.97 (0.88-1.07) | 0.479 | 0.859 |
| rs756627 | 8 | 145737286 | *RECQL4* | A | G | 0.45 | 0.46 | 0.97 (0.88-1.06) | 0.483 | 0.859 |
| rs7834823 | 8 | 9437352 | *TNKS* | A | C | 0.27 | 0.26 | 1.04 (0.93-1.16) | 0.485 | 0.859 |
| rs1805796 | 8 | 90993395 | *NBN* | A | G | 0.40 | 0.39 | 1.04 (0.94-1.14) | 0.485 | 0.859 |
| rs2898250 | 8 | 10621061 | *LOC102723313* | A | G | 0.11 | 0.12 | 0.95 (0.82-1.10) | 0.488 | 0.859 |
| rs2975852 | 8 | 73932430 | *TERF1* | A | G | 0.32 | 0.34 | 0.96 (0.87-1.07) | 0.488 | 0.859 |
| rs2297434 | 20 | 62294015 | *RTEL1*, *RTEL1-TNFRSF6B* | G | A | 0.47 | 0.48 | 0.97 (0.88-1.07) | 0.488 | 0.859 |
| rs3219095 | 1 | 226563882 | *PARP1* | G | A | 0.15 | 0.15 | 0.96 (0.84-1.09) | 0.510 | 0.892 |
| rs7162960 | 15 | 91287412 | *BLM* | G | A | 0.23 | 0.22 | 1.04 (0.92-1.17) | 0.516 | 0.895 |
| rs1078543 | 8 | 10623138 | *PINX1*, *LOC102723313* | T | A | 0.12 | 0.12 | 0.95 (0.82-1.11) | 0.522 | 0.899 |
| rs1801195 | 8 | 30999280 | *WRN* | A | C | 0.42 | 0.43 | 0.97 (0.88-1.07) | 0.531 | 0.902 |
| rs405684 | 17 | 56777148 | *RAD51C* | A | G | 0.38 | 0.39 | 0.97 (0.88-1.07) | 0.531 | 0.902 |
| rs909341 | 20 | 62328742 | *TNFRSF6B*, *RTEL1-TNFRSF6B* | A | G | 0.22 | 0.22 | 0.96 (0.86-1.09) | 0.535 | 0.902 |
| rs7307064 | 12 | 21634394 | *RECQL* | C | A | 0.12 | 0.12 | 1.05 (0.90-1.22) | 0.540 | 0.904 |
| rs7083959 | 10 | 93585997 | *TNKS2* | C | A | 0.18 | 0.17 | 1.04 (0.91-1.18) | 0.569 | 0.930 |
| rs2297437 | 20 | 62305274 | *RTEL1*, *RTEL1-TNFRSF6B* | A | G | 0.18 | 0.20 | 0.96 (0.85-1.09) | 0.569 | 0.930 |
| rs2192170 | 12 | 21633036 | *RECQL* | A | G | 0.10 | 0.09 | 1.05 (0.89-1.24) | 0.570 | 0.930 |
| rs2158177 | 5 | 131984058 | *TH2LCRR* | G | A | 0.21 | 0.21 | 1.03 (0.92-1.16) | 0.573 | 0.930 |
| rs444325 | 15 | 91364015 | - | A | C | 0.34 | 0.35 | 0.97 (0.87-1.08) | 0.581 | 0.930 |
| rs2292370 | 8 | 10690319 | *PINX1* | A | G | 0.30 | 0.30 | 0.97 (0.87-1.08) | 0.581 | 0.930 |
| rs10069690 | 5 | 1279790 | *TERT* | A | G | 0.28 | 0.27 | 1.03 (0.93-1.15) | 0.585 | 0.930 |
| rs6994361 | 8 | 31018685 | *WRN* | G | A | 0.35 | 0.36 | 0.97 (0.88-1.08) | 0.591 | 0.930 |
| rs4809324 | 20 | 62318220 | *RTEL1*, *RTEL1-TNFRSF6B* | G | A | 0.10 | 0.11 | 0.96 (0.82-1.12) | 0.591 | 0.930 |
| rs3093933 | 14 | 20824415 | *PARP2* | A | C | 0.26 | 0.25 | 1.03 (0.93-1.14) | 0.604 | 0.943 |
| rs4246977 | 14 | 20882591 | - | G | A | 0.38 | 0.38 | 0.98 (0.88-1.08) | 0.618 | 0.943 |
| rs7072165 | 10 | 93565641 | *TNKS2* | G | A | 0.10 | 0.11 | 0.96 (0.82-1.12) | 0.618 | 0.943 |
| rs12653750 | 5 | 131971902 | *TH2-LCR*, *RAD50*, *TH2LCRR* | A | G | 0.22 | 0.22 | 1.03 (0.92-1.16) | 0.621 | 0.943 |
| rs227060 | 11 | 108204881 | *ATM*, *C11orf65* | A | G | 0.32 | 0.32 | 1.03 (0.93-1.14) | 0.624 | 0.943 |
| rs11785739 | 8 | 9640400 | - | A | C | 0.07 | 0.08 | 0.95 (0.79-1.15) | 0.625 | 0.943 |
| rs6871536 | 5 | 131969874 | *TH2-LCR*, *RAD50*, *TH2LCRR* | G | A | 0.23 | 0.22 | 1.03 (0.92-1.15) | 0.638 | 0.943 |
| rs1551655 | 8 | 48873261 | *LOC106903146* | C | A | 0.08 | 0.08 | 1.05 (0.87-1.26) | 0.639 | 0.943 |
| rs6601338 | 8 | 9438928 | *TNKS* | A | G | 0.38 | 0.39 | 0.98 (0.89-1.08) | 0.645 | 0.943 |
| rs2240032 | 5 | 131977127 | *TH2-LCR*, *RAD50*, *TH2LCRR* | A | G | 0.22 | 0.22 | 1.03 (0.92-1.15) | 0.660 | 0.943 |
| rs1805794 | 8 | 90990479 | *NBN* | C | G | 0.34 | 0.33 | 1.02 (0.92-1.13) | 0.660 | 0.943 |
| rs78087823 | 1 | 226583026 | *PARP1* | A | G | 0.06 | 0.05 | 1.05 (0.85-1.30) | 0.662 | 0.943 |
| rs3784780 | 15 | 91309475 | *BLM* | A | G | 0.17 | 0.16 | 1.03 (0.90-1.18) | 0.665 | 0.943 |
| rs2301713 | 5 | 131951996 | *RAD50* | G | A | 0.23 | 0.22 | 1.03 (0.91-1.15) | 0.669 | 0.943 |
| rs2234744 | 8 | 90970935 | *NBN* | A | G | 0.34 | 0.33 | 1.02 (0.92-1.13) | 0.674 | 0.943 |
| rs1713425 | 14 | 20858174 | *TEP1* | A | G | 0.44 | 0.44 | 1.02 (0.93-1.13) | 0.675 | 0.943 |
| rs2897443 | 5 | 131929594 | *RAD50* | A | C | 0.22 | 0.21 | 1.03 (0.91-1.15) | 0.676 | 0.943 |
| rs2237060 | 5 | 131970885 | *TH2-LCR*, *RAD50*, *TH2LCRR* | C | A | 0.37 | 0.37 | 0.98 (0.88-1.09) | 0.678 | 0.943 |
| rs7737470 | 5 | 131974063 | *TH2-LCR*, *RAD50*, *TH2LCRR* | T | A | 0.23 | 0.22 | 1.02 (0.91-1.15) | 0.686 | 0.943 |
| rs3213212 | 12 | 21635232 | *RECQL* | A | G | 0.44 | 0.45 | 0.98 (0.89-1.08) | 0.689 | 0.943 |
| rs10464529 | 7 | 124520308 | *POT1* | A | G | 0.26 | 0.26 | 1.02 (0.92-1.14) | 0.689 | 0.943 |
| rs917855 | 12 | 21630146 | *RECQL* | A | G | 0.50 | 0.49 | 1.02 (0.92-1.13) | 0.693 | 0.943 |
| rs3798134 | 5 | 131965179 | *TH2-LCR*, *RAD50* | A | G | 0.23 | 0.22 | 1.02 (0.91-1.15) | 0.698 | 0.943 |
| rs12334811 | 8 | 48832977 | *PRKDC* | A | G | 0.07 | 0.07 | 1.04 (0.86-1.26) | 0.700 | 0.943 |
| rs389480 | 15 | 91351930 | *BLM* | A | G | 0.46 | 0.46 | 0.98 (0.89-1.09) | 0.711 | 0.943 |
| rs4733220 | 8 | 30900890 | *WRN* | A | G | 0.42 | 0.42 | 1.02 (0.92-1.13) | 0.713 | 0.943 |
| rs1805818 | 8 | 90970862 | *NBN* | A | C | 0.34 | 0.33 | 1.02 (0.92-1.13) | 0.714 | 0.943 |
| rs12545912 | 8 | 9601699 | *TNKS* | C | A | 0.27 | 0.27 | 1.02 (0.92-1.14) | 0.714 | 0.943 |
| rs2975842 | 8 | 73925591 | *TERF1* | A | G | 0.44 | 0.45 | 0.98 (0.89-1.08) | 0.717 | 0.943 |
| rs10954778 | 8 | 31008747 | *WRN* | G | A | 0.32 | 0.31 | 1.02 (0.92-1.13) | 0.720 | 0.943 |
| rs9649886 | 8 | 30962693 | *WRN* | C | A | 0.39 | 0.38 | 1.02 (0.92-1.13) | 0.727 | 0.943 |
| rs1063045 | 8 | 90995019 | *NBN* | A | G | 0.34 | 0.33 | 1.02 (0.92-1.13) | 0.734 | 0.943 |
| rs820206 | 17 | 73641714 | *RECQL5* | A | G | 0.36 | 0.35 | 1.02 (0.92-1.13) | 0.734 | 0.943 |
| rs2073635 | 8 | 90995366 | *NBN* | A | G | 0.34 | 0.33 | 1.02 (0.92-1.13) | 0.735 | 0.943 |
| rs2230009 | 8 | 30921935 | *WRN* | A | G | 0.06 | 0.06 | 1.03 (0.84-1.26) | 0.753 | 0.949 |
| rs1063053 | 8 | 90947537 | *NBN*, *OSGIN2* | A | G | 0.33 | 0.32 | 0.98 (0.89-1.09) | 0.759 | 0.949 |
| rs2299014 | 5 | 131903399 | *RAD50* | C | A | 0.41 | 0.41 | 0.98 (0.89-1.09) | 0.759 | 0.949 |
| rs6982126 | 8 | 73939623 | *TERF1* | A | G | 0.23 | 0.23 | 0.98 (0.87-1.11) | 0.770 | 0.949 |
| rs1346044 | 8 | 31024654 | *WRN* | G | A | 0.26 | 0.26 | 0.98 (0.88-1.10) | 0.770 | 0.949 |
| rs3219123 | 1 | 226555348 | *PARP1* | A | G | 0.05 | 0.05 | 1.03 (0.83-1.28) | 0.773 | 0.949 |
| rs820210 | 17 | 73650495 | *LOC107985013*, *RECQL5* | G | A | 0.35 | 0.35 | 1.02 (0.92-1.13) | 0.773 | 0.949 |
| rs3761124 | 20 | 62288752 | - | A | G | 0.22 | 0.21 | 1.02 (0.90-1.15) | 0.774 | 0.949 |
| rs3087409 | 8 | 30946403 | *WRN* | A | C | 0.06 | 0.06 | 1.03 (0.84-1.26) | 0.776 | 0.949 |
| rs6596086 | 5 | 131952222 | *RAD50* | G | A | 0.23 | 0.23 | 1.02 (0.91-1.14) | 0.796 | 0.957 |
| rs938886 | 14 | 20837701 | *TEP1* | G | C | 0.24 | 0.24 | 1.02 (0.91-1.14) | 0.797 | 0.957 |
| rs10843881 | 12 | 31241345 | *DDX11* | G | A | 0.45 | 0.46 | 0.99 (0.89-1.09) | 0.798 | 0.957 |
| rs4549545 | 5 | 177584923 | - | G | A | 0.19 | 0.19 | 0.98 (0.87-1.12) | 0.809 | 0.957 |
| rs2853677 | 5 | 1287194 | *LOC110806264*, *TERT* | G | A | 0.41 | 0.41 | 1.01 (0.92-1.11) | 0.822 | 0.957 |
| rs1760898 | 14 | 20872881 | *TEP1* | A | C | 0.22 | 0.23 | 0.99 (0.87-1.12) | 0.825 | 0.957 |
| rs12187537 | 5 | 131939904 | *RAD50* | C | A | 0.21 | 0.21 | 1.01 (0.90-1.14) | 0.825 | 0.957 |
| rs13273033 | 8 | 9540693 | *TNKS* | G | A | 0.27 | 0.27 | 1.01 (0.91-1.13) | 0.826 | 0.957 |
| rs1341263 | 10 | 93556855 | *TNKS2-AS1* | G | A | 0.38 | 0.38 | 0.99 (0.89-1.09) | 0.827 | 0.957 |
| rs2238337 | 15 | 91355732 | *BLM* | A | G | 0.36 | 0.36 | 0.99 (0.89-1.10) | 0.829 | 0.957 |
| rs11776767 | 8 | 10683929 | *PINX1* | C | G | 0.34 | 0.34 | 0.99 (0.89-1.10) | 0.829 | 0.957 |
| rs11249943 | 8 | 9607863 | *TNKS* | C | A | 0.21 | 0.21 | 1.01 (0.90-1.14) | 0.837 | 0.957 |
| rs2244012 | 5 | 131901225 | *RAD50* | G | A | 0.23 | 0.23 | 1.01 (0.90-1.14) | 0.839 | 0.957 |
| rs748883 | 1 | 226582325 | *PARP1* | G | A | 0.27 | 0.28 | 0.99 (0.88-1.11) | 0.847 | 0.957 |
| rs2107465 | 8 | 91001024 | - | A | G | 0.30 | 0.30 | 0.99 (0.89-1.10) | 0.848 | 0.957 |
| rs2725344 | 8 | 30918098 | *WRN* | G | A | 0.08 | 0.08 | 0.98 (0.82-1.18) | 0.850 | 0.957 |
| rs8031341 | 15 | 91313224 | *BLM* | G | A | 0.21 | 0.21 | 1.01 (0.89-1.15) | 0.852 | 0.957 |
| rs3219142 | 1 | 226552068 | *PARP1* | A | G | 0.19 | 0.19 | 0.99 (0.87-1.12) | 0.863 | 0.963 |
| rs304268 | 17 | 56799907 | *RAD51C* | G | A | 0.31 | 0.31 | 0.99 (0.89-1.10) | 0.865 | 0.963 |
| rs4987034 | 8 | 30977696 | *WRN* | G | A | 0.19 | 0.18 | 1.01 (0.89-1.15) | 0.874 | 0.964 |
| rs1136410 | 1 | 226555302 | *PARP1* | G | A | 0.20 | 0.20 | 1.01 (0.89-1.15) | 0.880 | 0.964 |
| rs7150689 | 14 | 20842964 | *TEP1* | G | A | 0.25 | 0.25 | 1.01 (0.90-1.13) | 0.880 | 0.964 |
| rs4668936 | 2 | 15731605 | - | G | A | 0.40 | 0.40 | 1.01 (0.91-1.11) | 0.882 | 0.964 |
| rs1713449 | 14 | 20841707 | *TEP1* | A | G | 0.23 | 0.24 | 0.99 (0.88-1.12) | 0.904 | 0.977 |
| rs2074369 | 5 | 131973663 | *TH2-LCR*, *RAD50*, *TH2LCRR* | G | A | 0.23 | 0.23 | 1.01 (0.90-1.13) | 0.904 | 0.977 |
| rs6010620 | 20 | 62309839 | *RTEL1*, *RTEL1-TNFRSF6B* | A | G | 0.22 | 0.22 | 1.01 (0.90-1.13) | 0.908 | 0.977 |
| rs1760903 | 14 | 20852817 | *TEP1* | A | G | 0.46 | 0.46 | 1.01 (0.91-1.11) | 0.911 | 0.977 |
| rs2040704 | 5 | 131973177 | *TH2-LCR*, *RAD50*, *TH2LCRR* | G | A | 0.23 | 0.23 | 1.01 (0.90-1.13) | 0.915 | 0.977 |
| rs1772188 | 10 | 93616628 | *TNKS2* | A | C | 0.15 | 0.14 | 1.01 (0.87-1.16) | 0.931 | 0.984 |
| rs1882928 | 8 | 31023822 | *WRN* | G | A | 0.42 | 0.42 | 1.00 (0.90-1.10) | 0.938 | 0.984 |
| rs3219038 | 1 | 226578809 | *PARP1* | A | G | 0.11 | 0.11 | 1.01 (0.87-1.17) | 0.938 | 0.984 |
| rs10492117 | 12 | 21659368 | *GOLT1B* | G | A | 0.50 | 0.50 | 1.00 (0.91-1.11) | 0.938 | 0.984 |
| rs2706347 | 5 | 131905117 | *RAD50* | A | C | 0.22 | 0.22 | 1.00 (0.89-1.12) | 0.947 | 0.989 |
| rs1760904 | 14 | 20852029 | *TEP1* | G | A | 0.48 | 0.48 | 1.00 (0.91-1.10) | 0.961 | 0.992 |
| rs1048771 | 1 | 46743900 | *LRRC41*, *RAD54L* | A | G | 0.11 | 0.11 | 1.00 (0.85-1.16) | 0.963 | 0.992 |
| rs907190 | 1 | 226566726 | *PARP1* | A | C | 0.20 | 0.20 | 1.00 (0.88-1.14) | 0.965 | 0.992 |
| rs11542623 | 1 | 46746164 | *LRRC41* | A | G | 0.11 | 0.11 | 1.00 (0.85-1.16) | 0.970 | 0.992 |
| rs17711777 | 8 | 10623240 | *PINX1*, *LOC102723313* | G | A | 0.08 | 0.08 | 1.00 (0.83-1.20) | 0.974 | 0.992 |
| rs10849087 | 12 | 4650065 | *RAD51AP1* | A | G | 0.21 | 0.21 | 1.00 (0.89-1.12) | 0.977 | 0.992 |
| rs2725385 | 8 | 30928146 | *WRN* | A | G | 0.28 | 0.28 | 1.00 (0.90-1.12) | 0.989 | 0.992 |
| rs820196 | 17 | 73627539 | *RECQL5* | G | A | 0.23 | 0.23 | 1.00 (0.89-1.12) | 0.990 | 0.992 |
| rs2270132 | 15 | 91351868 | *BLM* | C | A | 0.41 | 0.42 | 1.00 (0.90-1.11) | 0.991 | 0.992 |
| rs2706348 | 5 | 131905810 | *RAD50* | A | G | 0.22 | 0.22 | 1.00 (0.89-1.12) | 0.992 | 0.992 |

Chromosomal positions are based on the Genome Reference Consortium Human Build 37 (GRCh37) of the single nucleotide polymorphism database (dbSNP) build 151, MAF; Minor allele frequency, FDR; False discovery rate.

**Supplementary Table S5**: Assessment of 236 SNPs associated with the telomere length with risk for mIAA-first. Risk was estimated as Hazard ratios and 95% confidence intervals.

| **SNP IDs** | **Chromosome** | **Position** | **Gene** | **Minor allele** | **Major allele** | **MAF in cases** | **MAF in controls** | **HR (95% CI)** | ***P*-value** | **FDR adjusted *P*-value** |
| --- | --- | --- | --- | --- | --- | --- | --- | --- | --- | --- |
| rs4353905 | 4 | 110745938 | - | G | A | 0.19 | 0.16 | 1.32 (1.08-1.62) | 0.006 | 0.629 |
| rs1941526 | 18 | 39652939 | *PIK3C3* | A | G | 0.20 | 0.25 | 0.76 (0.62-0.93) | 0.006 | 0.629 |
| rs11082257 | 18 | 39570683 | *PIK3C3* | G | A | 0.19 | 0.24 | 0.76 (0.62-0.93) | 0.008 | 0.629 |
| rs12706627 | 7 | 124550133 | *POT1* | G | A | 0.35 | 0.31 | 1.24 (1.05-1.46) | 0.012 | 0.708 |
| rs2291207 | 7 | 124569382 | *POT1* | G | A | 0.35 | 0.31 | 1.19 (1.01-1.40) | 0.034 | 0.733 |
| rs3017077 | 11 | 94175249 | *MRE11* | A | G | 0.31 | 0.34 | 0.83 (0.69-0.99) | 0.038 | 0.733 |
| rs444325 | 15 | 91364015 | - | A | C | 0.31 | 0.35 | 0.83 (0.69-0.99) | 0.040 | 0.733 |
| rs2270132 | 15 | 91351868 | *BLM* | C | A | 0.37 | 0.42 | 0.84 (0.70-0.99) | 0.041 | 0.733 |
| rs1801516 | 11 | 108175462 | *ATM* | A | G | 0.19 | 0.15 | 1.24 (1.01-1.53) | 0.041 | 0.733 |
| rs11249930 | 8 | 9472445 | *TNKS* | G | A | 0.23 | 0.19 | 1.22 (1.01-1.49) | 0.041 | 0.733 |
| rs4463363 | 7 | 124513770 | *POT1* | A | G | 0.39 | 0.43 | 0.85 (0.72-1.00) | 0.045 | 0.733 |
| rs1944967 | 18 | 39531139 | - | G | A | 0.34 | 0.39 | 0.84 (0.71-1.00) | 0.048 | 0.733 |
| rs7170919 | 15 | 91314959 | *BLM* | A | G | 0.33 | 0.29 | 1.20 (1.00-1.43) | 0.052 | 0.733 |
| rs75245322 | 5 | 131903026 | *RAD50* | C | A | 0.10 | 0.08 | 1.32 (0.99-1.76) | 0.056 | 0.733 |
| rs654718 | 11 | 94190115 | *MRE11* | G | A | 0.31 | 0.34 | 0.84 (0.70-1.01) | 0.056 | 0.733 |
| rs962863 | 7 | 124558339 | *POT1* | A | G | 0.39 | 0.42 | 0.86 (0.73-1.01) | 0.061 | 0.733 |
| rs11930711 | 4 | 110734498 | - | A | G | 0.11 | 0.09 | 1.27 (0.99-1.65) | 0.064 | 0.733 |
| rs17772583 | 5 | 131953510 | *RAD50* | G | A | 0.27 | 0.23 | 1.19 (0.99-1.44) | 0.065 | 0.733 |
| rs4427176 | 8 | 9533399 | *TNKS* | A | C | 0.22 | 0.19 | 1.20 (0.99-1.45) | 0.068 | 0.733 |
| rs3093933 | 14 | 20824415 | *PARP2* | A | C | 0.29 | 0.25 | 1.17 (0.99-1.37) | 0.068 | 0.733 |
| rs16944894 | 15 | 91363303 | - | G | A | 0.22 | 0.19 | 1.20 (0.99-1.45) | 0.071 | 0.733 |
| rs2072352 | 15 | 91334179 | *BLM* | A | G | 0.32 | 0.29 | 1.17 (0.99-1.38) | 0.073 | 0.733 |
| rs17711777 | 8 | 10623240 | *PINX1*, *LOC102723313* | G | A | 0.10 | 0.08 | 1.29 (0.97-1.70) | 0.076 | 0.733 |
| rs909341 | 20 | 62328742 | *TNFRSF6B*, *RTEL1-TNFRSF6B* | A | G | 0.19 | 0.22 | 0.84 (0.68-1.02) | 0.078 | 0.733 |
| rs628728 | 11 | 94250379 | *C11orf97* | A | G | 0.38 | 0.42 | 0.86 (0.73-1.02) | 0.078 | 0.733 |
| rs12679892 | 8 | 9445429 | *TNKS* | G | A | 0.34 | 0.31 | 1.16 (0.98-1.38) | 0.087 | 0.733 |
| rs11211262 | 1 | 46721155 | *RAD54L* | A | G | 0.37 | 0.40 | 0.86 (0.73-1.02) | 0.087 | 0.733 |
| rs7159947 | 14 | 20809491 | - | G | A | 0.39 | 0.36 | 1.14 (0.98-1.34) | 0.087 | 0.733 |
| rs7175811 | 15 | 91331546 | *BLM* | A | G | 0.40 | 0.36 | 1.15 (0.98-1.35) | 0.092 | 0.743 |
| rs7167216 | 15 | 91354521 | *BLM* | A | G | 0.10 | 0.08 | 1.27 (0.96-1.67) | 0.096 | 0.743 |
| rs1760897 | 14 | 20876253 | *TEP1* | G | A | 0.28 | 0.31 | 0.87 (0.73-1.03) | 0.098 | 0.743 |
| rs1048771 | 1 | 46743900 | *LRRC41*, *RAD54L* | A | G | 0.09 | 0.11 | 0.79 (0.59-1.05) | 0.106 | 0.743 |
| rs11542623 | 1 | 46746164 | *LRRC41* | A | G | 0.09 | 0.11 | 0.79 (0.59-1.05) | 0.106 | 0.743 |
| rs7015700 | 8 | 9527707 | *TNKS* | A | G | 0.17 | 0.20 | 0.83 (0.67-1.04) | 0.107 | 0.743 |
| rs604845 | 11 | 94182689 | *MRE11* | A | G | 0.36 | 0.33 | 1.15 (0.96-1.37) | 0.121 | 0.813 |
| rs389480 | 15 | 91351930 | *BLM* | A | G | 0.43 | 0.47 | 0.88 (0.74-1.04) | 0.124 | 0.813 |
| rs11249943 | 8 | 9607863 | *TNKS* | C | A | 0.24 | 0.21 | 1.15 (0.96-1.39) | 0.128 | 0.816 |
| rs6601333 | 8 | 9427048 | *TNKS* | G | A | 0.29 | 0.25 | 1.15 (0.96-1.38) | 0.135 | 0.817 |
| rs12545912 | 8 | 9601699 | *TNKS* | C | A | 0.30 | 0.27 | 1.15 (0.96-1.37) | 0.135 | 0.817 |
| rs2736100 | 5 | 1286516 | *TERT* | C | A | 0.47 | 0.50 | 1.13 (0.96-1.33) | 0.139 | 0.820 |
| rs2228041 | 14 | 20852267 | *TEP1* | A | G | 0.07 | 0.06 | 1.25 (0.92-1.71) | 0.151 | 0.854 |
| rs13250838 | 8 | 9526345 | *TNKS* | A | G | 0.10 | 0.13 | 0.82 (0.63-1.08) | 0.152 | 0.854 |
| rs2104978 | 14 | 20837033 | *TEP1* | G | A | 0.08 | 0.06 | 1.24 (0.91-1.68) | 0.172 | 0.896 |
| rs12142240 | 1 | 46747301 | *LRRC41* | G | A | 0.33 | 0.30 | 1.13 (0.95-1.35) | 0.174 | 0.896 |
| rs733396 | 8 | 10621981 | *LOC102723313* | A | G | 0.48 | 0.44 | 1.12 (0.95-1.33) | 0.180 | 0.896 |
| rs6470522 | 8 | 90954481 | *NBN* | A | G | 0.15 | 0.17 | 0.86 (0.69-1.07) | 0.181 | 0.896 |
| rs13265363 | 8 | 9422926 | *TNKS* | C | A | 0.11 | 0.14 | 0.84 (0.65-1.09) | 0.182 | 0.896 |
| rs7184015 | 15 | 91274575 | *BLM* | A | C | 0.31 | 0.28 | 1.12 (0.95-1.34) | 0.185 | 0.896 |
| rs6601530 | 8 | 10671272 | *PINX1* | G | A | 0.44 | 0.47 | 0.89 (0.75-1.06) | 0.186 | 0.896 |
| rs7162960 | 15 | 91287412 | *BLM* | G | A | 0.25 | 0.22 | 1.14 (0.94-1.37) | 0.191 | 0.898 |
| rs13273033 | 8 | 9540693 | *TNKS* | G | A | 0.29 | 0.27 | 1.13 (0.94-1.34) | 0.194 | 0.898 |
| rs11250076 | 8 | 10647823 | *PINX1* | A | G | 0.44 | 0.47 | 0.90 (0.76-1.06) | 0.201 | 0.910 |
| rs3761124 | 20 | 62288752 | - | A | G | 0.20 | 0.21 | 0.88 (0.72-1.07) | 0.208 | 0.910 |
| rs7302664 | 12 | 4664821 | *RAD51AP1* | A | G | 0.11 | 0.10 | 1.17 (0.92-1.49) | 0.211 | 0.910 |
| rs6010620 | 20 | 62309839 | *RTEL1*, *RTEL1-TNFRSF6B* | A | G | 0.20 | 0.22 | 0.88 (0.72-1.08) | 0.212 | 0.910 |
| rs4809324 | 20 | 62318220 | *RTEL1*, *RTEL1-TNFRSF6B* | G | A | 0.12 | 0.11 | 1.16 (0.92-1.47) | 0.218 | 0.919 |
| rs8022805 | 14 | 20846950 | *TEP1* | A | G | 0.07 | 0.05 | 1.22 (0.89-1.69) | 0.223 | 0.923 |
| rs7834823 | 8 | 9437352 | *TNKS* | A | C | 0.28 | 0.26 | 1.12 (0.93-1.34) | 0.237 | 0.948 |
| rs9282580 | 1 | 226594372 | *PARP1* | A | G | 0.04 | 0.05 | 0.77 (0.50-1.19) | 0.238 | 0.948 |
| rs2238337 | 15 | 91355732 | *BLM* | A | G | 0.39 | 0.36 | 1.11 (0.93-1.31) | 0.242 | 0.948 |
| rs3755132 | 2 | 15729820 | - | C | A | 0.18 | 0.16 | 1.15 (0.91-1.44) | 0.245 | 0.948 |
| rs17772565 | 5 | 131952405 | *RAD50* | A | G | 0.05 | 0.06 | 0.80 (0.55-1.18) | 0.263 | 0.974 |
| rs3755133 | 2 | 15731583 | - | A | G | 0.18 | 0.17 | 1.14 (0.91-1.43) | 0.265 | 0.974 |
| rs3219038 | 1 | 226578809 | *PARP1* | A | G | 0.13 | 0.11 | 1.15 (0.90-1.47) | 0.273 | 0.974 |
| rs8031341 | 15 | 91313224 | *BLM* | G | A | 0.22 | 0.21 | 1.12 (0.91-1.38) | 0.274 | 0.974 |
| rs10069690 | 5 | 1279790 | *TERT* | A | G | 0.30 | 0.27 | 1.10 (0.93-1.31) | 0.282 | 0.974 |
| rs7826180 | 8 | 10627844 | *PINX1*, *LOC102723313* | A | G | 0.24 | 0.22 | 1.11 (0.91-1.35) | 0.290 | 0.974 |
| rs6601524 | 8 | 10635222 | *PINX1* | A | C | 0.23 | 0.22 | 1.12 (0.91-1.36) | 0.292 | 0.974 |
| rs4982038 | 14 | 20862542 | *TEP1* | A | G | 0.30 | 0.32 | 0.91 (0.77-1.08) | 0.293 | 0.974 |
| rs7838341 | 8 | 10661734 | *PINX1* | G | A | 0.24 | 0.23 | 1.11 (0.91-1.35) | 0.298 | 0.974 |
| rs2297434 | 20 | 62294015 | *RTEL1*, *RTEL1-TNFRSF6B* | G | A | 0.45 | 0.48 | 0.92 (0.79-1.08) | 0.299 | 0.974 |
| rs2975842 | 8 | 73925591 | *TERF1* | A | G | 0.46 | 0.45 | 1.09 (0.93-1.27) | 0.301 | 0.974 |
| rs1805796 | 8 | 90993395 | *NBN* | A | G | 0.41 | 0.39 | 1.09 (0.92-1.28) | 0.310 | 0.974 |
| rs663530 | 11 | 94157114 | *MRE11* | A | G | 0.25 | 0.26 | 0.90 (0.74-1.10) | 0.312 | 0.974 |
| rs976016 | 2 | 15772865 | - | G | A | 0.19 | 0.17 | 1.12 (0.90-1.40) | 0.312 | 0.974 |
| rs2237060 | 5 | 131970885 | *TH2-LCR*, *RAD50*, *TH2LCRR* | C | A | 0.36 | 0.37 | 0.91 (0.76-1.10) | 0.318 | 0.974 |
| rs11160462 | 14 | 20868751 | *TEP1* | A | G | 0.35 | 0.37 | 0.92 (0.77-1.09) | 0.329 | 0.974 |
| rs538800 | 11 | 94239567 | *MRE11* | A | G | 0.35 | 0.33 | 1.09 (0.91-1.30) | 0.338 | 0.974 |
| rs4549545 | 5 | 177584923 | - | G | A | 0.17 | 0.19 | 0.90 (0.72-1.12) | 0.344 | 0.974 |
| rs78733221 | 8 | 9476448 | *TNKS* | G | A | 0.07 | 0.08 | 0.86 (0.62-1.19) | 0.363 | 0.974 |
| rs6982126 | 8 | 73939623 | *TERF1* | A | G | 0.21 | 0.23 | 0.91 (0.75-1.11) | 0.363 | 0.974 |
| rs13447720 | 11 | 94165326 | *MRE11* | G | A | 0.21 | 0.23 | 0.91 (0.74-1.12) | 0.368 | 0.974 |
| rs1805793 | 8 | 90990091 | *NBN* | G | A | 0.41 | 0.39 | 1.08 (0.92-1.27) | 0.368 | 0.974 |
| rs2945461 | 14 | 20863070 | *TEP1* | G | A | 0.08 | 0.10 | 0.87 (0.65-1.17) | 0.368 | 0.974 |
| rs709816 | 8 | 90967711 | *NBN* | G | A | 0.41 | 0.39 | 1.08 (0.92-1.27) | 0.372 | 0.974 |
| rs6011011 | 20 | 62299578 | *RTEL1*, *RTEL1-TNFRSF6B* | A | G | 0.06 | 0.08 | 0.86 (0.63-1.19) | 0.376 | 0.974 |
| rs619972 | 11 | 108169619 | *ATM* | G | A | 0.42 | 0.44 | 0.93 (0.79-1.09) | 0.376 | 0.974 |
| rs7307064 | 12 | 21634394 | *RECQL* | C | A | 0.12 | 0.12 | 1.12 (0.87-1.45) | 0.376 | 0.974 |
| rs11776767 | 8 | 10683929 | *PINX1* | C | G | 0.35 | 0.34 | 1.09 (0.91-1.30) | 0.378 | 0.974 |
| rs11250077 | 8 | 10656437 | *PINX1* | A | G | 0.40 | 0.42 | 0.93 (0.78-1.10) | 0.390 | 0.974 |
| rs10090277 | 8 | 9450886 | *TNKS* | G | A | 0.07 | 0.08 | 0.87 (0.62-1.20) | 0.390 | 0.974 |
| rs600931 | 11 | 108117335 | *ATM* | G | A | 0.42 | 0.44 | 0.93 (0.79-1.10) | 0.395 | 0.974 |
| rs61909598 | 12 | 4671930 | *DYRK4* | A | C | 0.13 | 0.12 | 1.10 (0.88-1.39) | 0.408 | 0.974 |
| rs820206 | 17 | 73641714 | *RECQL5* | A | G | 0.37 | 0.35 | 1.08 (0.90-1.28) | 0.417 | 0.974 |
| rs4660918 | 1 | 46731694 | *RAD54L* | G | A | 0.25 | 0.26 | 0.93 (0.77-1.11) | 0.419 | 0.974 |
| rs1800391 | 8 | 30938704 | *WRN* | A | G | 0.07 | 0.08 | 0.87 (0.63-1.22) | 0.421 | 0.974 |
| rs4796033 | 17 | 33433487 | *RAD51D*, *RAD51L3-RFFL* | A | G | 0.14 | 0.15 | 0.91 (0.72-1.15) | 0.423 | 0.974 |
| rs114939615 | 1 | 226591587 | *PARP1* | A | G | 0.07 | 0.07 | 1.13 (0.84-1.53) | 0.426 | 0.974 |
| rs609261 | 11 | 108158134 | *ATM* | A | G | 0.42 | 0.44 | 0.94 (0.79-1.10) | 0.428 | 0.974 |
| rs3848668 | 20 | 62293272 | *RTEL1*, *RTEL1-TNFRSF6B* | G | A | 0.06 | 0.07 | 0.88 (0.63-1.22) | 0.428 | 0.974 |
| rs12125573 | 1 | 46734792 | *RAD54L* | G | A | 0.28 | 0.30 | 0.93 (0.78-1.11) | 0.428 | 0.974 |
| rs2227933 | 15 | 91337479 | *BLM* | A | G | 0.19 | 0.18 | 1.09 (0.88-1.34) | 0.432 | 0.974 |
| rs2234744 | 8 | 90970935 | *NBN* | A | G | 0.35 | 0.33 | 1.07 (0.90-1.27) | 0.435 | 0.974 |
| rs599558 | 11 | 108177538 | *ATM* | G | A | 0.42 | 0.44 | 0.94 (0.80-1.11) | 0.441 | 0.974 |
| rs1871892 | 17 | 33448818 | *FNDC8* | A | G | 0.31 | 0.32 | 0.93 (0.78-1.12) | 0.443 | 0.974 |
| rs1805794 | 8 | 90990479 | *NBN* | C | G | 0.35 | 0.33 | 1.07 (0.90-1.27) | 0.448 | 0.974 |
| rs820210 | 17 | 73650495 | *LOC107985013*, *RECQL5* | G | A | 0.37 | 0.35 | 1.07 (0.90-1.27) | 0.459 | 0.974 |
| rs4585 | 11 | 108239628 | *ATM*, *C11orf65* | C | A | 0.42 | 0.44 | 0.94 (0.80-1.11) | 0.460 | 0.974 |
| rs645485 | 11 | 108168863 | *ATM* | A | G | 0.42 | 0.44 | 0.94 (0.80-1.11) | 0.463 | 0.974 |
| rs61835377 | 1 | 226545854 | - | A | G | 0.18 | 0.20 | 0.92 (0.74-1.15) | 0.475 | 0.974 |
| rs2107465 | 8 | 91001024 | - | A | G | 0.32 | 0.30 | 1.06 (0.90-1.26) | 0.477 | 0.974 |
| rs2975852 | 8 | 73932430 | *TERF1* | A | G | 0.34 | 0.34 | 1.07 (0.90-1.27) | 0.478 | 0.974 |
| rs3755130 | 2 | 15729583 | - | A | G | 0.43 | 0.42 | 1.06 (0.90-1.25) | 0.481 | 0.974 |
| rs623860 | 11 | 108106782 | *ATM* | G | A | 0.41 | 0.42 | 0.94 (0.80-1.11) | 0.488 | 0.974 |
| rs1805818 | 8 | 90970862 | *NBN* | A | C | 0.35 | 0.33 | 1.06 (0.90-1.26) | 0.490 | 0.974 |
| rs12680047 | 8 | 128758861 | - | G | A | 0.38 | 0.39 | 0.94 (0.80-1.11) | 0.495 | 0.974 |
| rs10102170 | 8 | 9611470 | *TNKS* | A | G | 0.40 | 0.39 | 1.06 (0.90-1.25) | 0.496 | 0.974 |
| rs1063045 | 8 | 90995019 | *NBN* | A | G | 0.35 | 0.33 | 1.06 (0.90-1.26) | 0.496 | 0.974 |
| rs2073635 | 8 | 90995366 | *NBN* | A | G | 0.35 | 0.33 | 1.06 (0.90-1.26) | 0.497 | 0.974 |
| rs938886 | 14 | 20837701 | *TEP1* | G | C | 0.23 | 0.24 | 0.94 (0.78-1.13) | 0.500 | 0.974 |
| rs2110159 | 12 | 21649160 | *RECQL* | G | A | 0.49 | 0.50 | 0.95 (0.80-1.11) | 0.501 | 0.974 |
| rs7299185 | 12 | 4672394 | *DYRK4* | C | A | 0.24 | 0.25 | 0.94 (0.78-1.13) | 0.509 | 0.974 |
| rs1713449 | 14 | 20841707 | *TEP1* | A | G | 0.22 | 0.24 | 0.94 (0.78-1.14) | 0.509 | 0.974 |
| rs622961 | 11 | 94154902 | *MRE11* | A | G | 0.43 | 0.44 | 0.95 (0.80-1.12) | 0.512 | 0.974 |
| rs3784780 | 15 | 91309475 | *BLM* | A | G | 0.17 | 0.16 | 1.08 (0.86-1.35) | 0.531 | 0.978 |
| rs748883 | 1 | 226582325 | *PARP1* | G | A | 0.26 | 0.28 | 0.94 (0.77-1.15) | 0.537 | 0.978 |
| rs228591 | 11 | 108097333 | *ATM* | A | G | 0.42 | 0.44 | 0.95 (0.81-1.12) | 0.544 | 0.978 |
| rs3213212 | 12 | 21635232 | *RECQL* | A | G | 0.43 | 0.45 | 0.95 (0.81-1.12) | 0.553 | 0.978 |
| rs10101132 | 8 | 9616553 | *TNKS* | A | C | 0.33 | 0.32 | 1.05 (0.89-1.23) | 0.556 | 0.978 |
| rs17183344 | 15 | 91357488 | *BLM* | A | G | 0.19 | 0.18 | 1.06 (0.86-1.31) | 0.571 | 0.978 |
| rs33994795 | 8 | 9473429 | *TNKS* | G | A | 0.41 | 0.40 | 1.05 (0.89-1.23) | 0.586 | 0.978 |
| rs664143 | 11 | 108225661 | *ATM*, *C11orf65* | A | G | 0.41 | 0.43 | 0.96 (0.81-1.13) | 0.589 | 0.978 |
| rs2242652 | 5 | 1280028 | *TERT* | A | G | 0.22 | 0.21 | 1.05 (0.87-1.27) | 0.589 | 0.978 |
| rs425538 | 11 | 108219339 | *ATM*, *C11orf65* | C | A | 0.41 | 0.43 | 0.96 (0.81-1.13) | 0.593 | 0.978 |
| rs476137 | 11 | 94213905 | *MRE11* | A | C | 0.41 | 0.43 | 0.96 (0.81-1.13) | 0.597 | 0.978 |
| rs1713425 | 14 | 20858174 | *TEP1* | A | G | 0.45 | 0.44 | 1.05 (0.89-1.23) | 0.597 | 0.978 |
| rs17774023 | 8 | 10626333 | *PINX1*, *LOC102723313* | G | A | 0.31 | 0.30 | 1.05 (0.88-1.25) | 0.609 | 0.978 |
| rs4246977 | 14 | 20882591 | - | G | A | 0.37 | 0.38 | 0.96 (0.82-1.13) | 0.618 | 0.978 |
| rs10849087 | 12 | 4650065 | *RAD51AP1* | A | G | 0.22 | 0.21 | 1.05 (0.87-1.26) | 0.619 | 0.978 |
| rs6668851 | 1 | 226545866 | - | C | A | 0.31 | 0.30 | 1.05 (0.87-1.25) | 0.629 | 0.978 |
| rs16943176 | 17 | 56769887 | *RAD51C* | A | G | 0.22 | 0.22 | 0.95 (0.78-1.16) | 0.630 | 0.978 |
| rs10954778 | 8 | 31008747 | *WRN* | G | A | 0.32 | 0.31 | 1.04 (0.88-1.24) | 0.631 | 0.978 |
| rs7150689 | 14 | 20842964 | *TEP1* | G | A | 0.24 | 0.25 | 0.96 (0.80-1.15) | 0.637 | 0.978 |
| rs6601338 | 8 | 9438928 | *TNKS* | A | G | 0.39 | 0.39 | 1.04 (0.88-1.22) | 0.638 | 0.978 |
| rs10843881 | 12 | 31241345 | *DDX11* | G | A | 0.44 | 0.46 | 0.96 (0.82-1.13) | 0.638 | 0.978 |
| rs2292370 | 8 | 10690319 | *PINX1* | A | G | 0.31 | 0.30 | 1.05 (0.87-1.26) | 0.639 | 0.978 |
| rs10492117 | 12 | 21659368 | *GOLT1B* | G | A | 0.49 | 0.50 | 1.04 (0.88-1.23) | 0.641 | 0.978 |
| rs12567614 | 1 | 226544420 | - | A | G | 0.47 | 0.46 | 1.04 (0.88-1.24) | 0.650 | 0.978 |
| rs1805414 | 1 | 226573364 | *PARP1* | G | A | 0.37 | 0.37 | 1.04 (0.87-1.24) | 0.657 | 0.978 |
| rs3219125 | 1 | 226554951 | *PARP1* | G | A | 0.06 | 0.06 | 1.08 (0.77-1.52) | 0.659 | 0.978 |
| rs7083959 | 10 | 93585997 | *TNKS2* | C | A | 0.18 | 0.17 | 1.05 (0.84-1.31) | 0.676 | 0.978 |
| rs11998382 | 8 | 10659517 | *PINX1* | G | A | 0.24 | 0.22 | 1.04 (0.86-1.27) | 0.680 | 0.978 |
| rs3087409 | 8 | 30946403 | *WRN* | A | C | 0.06 | 0.06 | 0.93 (0.66-1.31) | 0.687 | 0.978 |
| rs3219095 | 1 | 226563882 | *PARP1* | G | A | 0.16 | 0.15 | 1.05 (0.84-1.30) | 0.691 | 0.978 |
| rs2297437 | 20 | 62305274 | *RTEL1*, *RTEL1-TNFRSF6B* | A | G | 0.19 | 0.20 | 1.04 (0.85-1.27) | 0.702 | 0.978 |
| rs820196 | 17 | 73627539 | *RECQL5* | G | A | 0.23 | 0.23 | 1.04 (0.86-1.26) | 0.707 | 0.978 |
| rs7737470 | 5 | 131974063 | *TH2-LCR*, *RAD50*, *TH2LCRR* | T | A | 0.23 | 0.22 | 1.04 (0.85-1.27) | 0.709 | 0.978 |
| rs6994361 | 8 | 31018685 | *WRN* | G | A | 0.35 | 0.36 | 0.97 (0.81-1.15) | 0.712 | 0.978 |
| rs227060 | 11 | 108204881 | *ATM*, *C11orf65* | A | G | 0.33 | 0.32 | 1.03 (0.87-1.23) | 0.713 | 0.978 |
| rs10464529 | 7 | 124520308 | *POT1* | A | G | 0.25 | 0.26 | 0.97 (0.80-1.17) | 0.724 | 0.978 |
| rs1805812 | 8 | 90965053 | *NBN* | G | A | 0.09 | 0.09 | 0.95 (0.73-1.25) | 0.730 | 0.978 |
| rs2725385 | 8 | 30928146 | *WRN* | A | G | 0.28 | 0.28 | 0.97 (0.81-1.16) | 0.736 | 0.978 |
| rs12334407 | 8 | 73953688 | *TERF1* | A | C | 0.32 | 0.32 | 1.03 (0.86-1.23) | 0.737 | 0.978 |
| rs9956832 | 18 | 39564876 | *PIK3C3* | A | C | 0.14 | 0.13 | 1.04 (0.83-1.31) | 0.748 | 0.978 |
| rs1882928 | 8 | 31023822 | *WRN* | G | A | 0.41 | 0.42 | 0.97 (0.82-1.15) | 0.748 | 0.978 |
| rs3798134 | 5 | 131965179 | *TH2-LCR*, *RAD50* | A | G | 0.23 | 0.22 | 1.03 (0.85-1.26) | 0.753 | 0.978 |
| rs12630450 | 3 | 169480204 | - | G | A | 0.29 | 0.29 | 1.03 (0.86-1.24) | 0.756 | 0.978 |
| rs1760898 | 14 | 20872881 | *TEP1* | A | C | 0.22 | 0.23 | 0.97 (0.79-1.19) | 0.756 | 0.978 |
| rs13276086 | 8 | 9578982 | *TNKS* | A | C | 0.40 | 0.39 | 1.03 (0.87-1.21) | 0.761 | 0.978 |
| rs9939870 | 16 | 69396585 | *TERF2* | A | G | 0.25 | 0.25 | 1.03 (0.86-1.23) | 0.766 | 0.978 |
| rs6871536 | 5 | 131969874 | *TH2-LCR*, *RAD50*, *TH2LCRR* | G | A | 0.23 | 0.22 | 1.03 (0.85-1.26) | 0.769 | 0.978 |
| rs9822885 | 3 | 169486144 | *ACTRT3* | G | A | 0.29 | 0.29 | 1.03 (0.86-1.24) | 0.770 | 0.978 |
| rs2299014 | 5 | 131903399 | *RAD50* | C | A | 0.41 | 0.41 | 0.98 (0.82-1.16) | 0.774 | 0.978 |
| rs2736122 | 5 | 1257621 | *TERT* | A | G | 0.23 | 0.24 | 0.97 (0.81-1.17) | 0.778 | 0.978 |
| rs2192170 | 12 | 21633036 | *RECQL* | A | G | 0.09 | 0.09 | 0.96 (0.73-1.27) | 0.778 | 0.978 |
| rs12653750 | 5 | 131971902 | *TH2-LCR*, *RAD50*, *TH2LCRR* | A | G | 0.23 | 0.22 | 1.03 (0.84-1.25) | 0.780 | 0.978 |
| rs756627 | 8 | 145737286 | *RECQL4* | A | G | 0.45 | 0.46 | 0.98 (0.84-1.14) | 0.781 | 0.978 |
| rs6984094 | 8 | 10645738 | *PINX1* | G | A | 0.12 | 0.12 | 0.97 (0.75-1.24) | 0.781 | 0.978 |
| rs414634 | 15 | 91356253 | *BLM* | A | C | 0.29 | 0.28 | 1.03 (0.86-1.23) | 0.787 | 0.978 |
| rs2230009 | 8 | 30921935 | *WRN* | A | G | 0.06 | 0.06 | 0.96 (0.68-1.34) | 0.790 | 0.978 |
| rs2853676 | 5 | 1288547 | *TERT* | A | G | 0.24 | 0.25 | 0.98 (0.81-1.18) | 0.806 | 0.978 |
| rs2301713 | 5 | 131951996 | *RAD50* | G | A | 0.23 | 0.22 | 1.03 (0.84-1.25) | 0.807 | 0.978 |
| rs1801195 | 8 | 30999280 | *WRN* | A | C | 0.42 | 0.43 | 0.98 (0.83-1.16) | 0.809 | 0.978 |
| rs936656 | 17 | 33433820 | *RAD51D*, *RAD51L3-RFFL* | G | A | 0.45 | 0.47 | 0.98 (0.83-1.16) | 0.810 | 0.978 |
| rs2158177 | 5 | 131984058 | *TH2LCRR* | G | A | 0.22 | 0.21 | 1.02 (0.84-1.25) | 0.813 | 0.978 |
| rs1136410 | 1 | 226555302 | *PARP1* | G | A | 0.21 | 0.20 | 1.03 (0.83-1.26) | 0.814 | 0.978 |
| rs7072165 | 10 | 93565641 | *TNKS2* | G | A | 0.10 | 0.11 | 0.97 (0.74-1.27) | 0.820 | 0.978 |
| rs1078543 | 8 | 10623138 | *PINX1*, *LOC102723313* | T | A | 0.12 | 0.12 | 0.97 (0.76-1.24) | 0.821 | 0.978 |
| rs6668722 | 1 | 226545776 | - | G | A | 0.10 | 0.10 | 1.03 (0.78-1.36) | 0.822 | 0.978 |
| rs2853672 | 5 | 1292983 | *TERT* | A | C | 0.50 | 0.50 | 1.02 (0.87-1.20) | 0.823 | 0.978 |
| rs2240032 | 5 | 131977127 | *TH2-LCR*, *RAD50*, *TH2LCRR* | A | G | 0.23 | 0.22 | 1.02 (0.84-1.25) | 0.827 | 0.978 |
| rs2898250 | 8 | 10621061 | *LOC102723313* | A | G | 0.12 | 0.12 | 0.97 (0.76-1.24) | 0.831 | 0.978 |
| rs10099824 | 8 | 73940350 | *TERF1* | A | G | 0.46 | 0.45 | 1.02 (0.86-1.20) | 0.833 | 0.978 |
| rs3219090 | 1 | 226564691 | *PARP1* | A | G | 0.37 | 0.37 | 1.02 (0.86-1.21) | 0.836 | 0.978 |
| rs4244612 | 8 | 145741702 | *RECQL4* | C | G | 0.40 | 0.40 | 0.98 (0.83-1.16) | 0.843 | 0.978 |
| rs12187537 | 5 | 131939904 | *RAD50* | C | A | 0.21 | 0.21 | 1.02 (0.84-1.25) | 0.846 | 0.978 |
| rs1063053 | 8 | 90947537 | *NBN*, *OSGIN2* | A | G | 0.33 | 0.32 | 1.02 (0.86-1.20) | 0.852 | 0.978 |
| rs1800392 | 8 | 30973957 | *WRN* | A | C | 0.43 | 0.44 | 0.99 (0.84-1.16) | 0.857 | 0.978 |
| rs2725344 | 8 | 30918098 | *WRN* | G | A | 0.08 | 0.08 | 1.03 (0.76-1.39) | 0.861 | 0.978 |
| rs2244012 | 5 | 131901225 | *RAD50* | G | A | 0.23 | 0.23 | 1.02 (0.83-1.24) | 0.862 | 0.978 |
| rs12638862 | 3 | 169477506 | - | G | A | 0.28 | 0.28 | 1.02 (0.84-1.23) | 0.863 | 0.978 |
| rs304268 | 17 | 56799907 | *RAD51C* | G | A | 0.31 | 0.31 | 0.99 (0.83-1.17) | 0.867 | 0.978 |
| rs4733220 | 8 | 30900890 | *WRN* | A | G | 0.42 | 0.42 | 0.99 (0.84-1.16) | 0.869 | 0.978 |
| rs2853677 | 5 | 1287194 | *LOC110806264*, *TERT* | G | A | 0.40 | 0.41 | 0.99 (0.84-1.16) | 0.870 | 0.978 |
| rs740059 | 12 | 4671490 | *LOC113939935*, *DYRK4* | G | A | 0.49 | 0.49 | 1.01 (0.86-1.19) | 0.873 | 0.978 |
| rs1760903 | 14 | 20852817 | *TEP1* | A | G | 0.46 | 0.46 | 0.99 (0.84-1.16) | 0.873 | 0.978 |
| rs907190 | 1 | 226566726 | *PARP1* | A | C | 0.21 | 0.20 | 1.02 (0.83-1.25) | 0.876 | 0.978 |
| rs2706348 | 5 | 131905810 | *RAD50* | A | G | 0.22 | 0.22 | 0.98 (0.80-1.21) | 0.876 | 0.978 |
| rs4668936 | 2 | 15731605 | - | G | A | 0.40 | 0.40 | 0.99 (0.84-1.16) | 0.876 | 0.978 |
| rs3102095 | 8 | 73916745 | - | C | A | 0.32 | 0.32 | 1.01 (0.85-1.21) | 0.880 | 0.978 |
| rs2227935 | 15 | 91326099 | *BLM* | A | G | 0.07 | 0.07 | 1.03 (0.74-1.41) | 0.881 | 0.978 |
| rs3219142 | 1 | 226552068 | *PARP1* | A | G | 0.19 | 0.19 | 0.98 (0.80-1.22) | 0.883 | 0.978 |
| rs4987034 | 8 | 30977696 | *WRN* | G | A | 0.19 | 0.18 | 1.02 (0.83-1.25) | 0.884 | 0.978 |
| rs78087823 | 1 | 226583026 | *PARP1* | A | G | 0.05 | 0.05 | 1.03 (0.72-1.47) | 0.894 | 0.978 |
| rs11785739 | 8 | 9640400 | - | A | C | 0.08 | 0.08 | 0.98 (0.72-1.34) | 0.896 | 0.978 |
| rs2293464 | 1 | 226576296 | *PARP1* | A | G | 0.16 | 0.16 | 1.01 (0.81-1.28) | 0.907 | 0.978 |
| rs59672299 | 1 | 226597593 | - | G | A | 0.16 | 0.16 | 1.01 (0.81-1.28) | 0.907 | 0.978 |
| rs1000033 | 1 | 226580387 | *PARP1* | C | A | 0.16 | 0.16 | 1.01 (0.80-1.28) | 0.914 | 0.978 |
| rs1346044 | 8 | 31024654 | *WRN* | G | A | 0.27 | 0.26 | 0.99 (0.82-1.19) | 0.914 | 0.978 |
| rs6596086 | 5 | 131952222 | *RAD50* | G | A | 0.23 | 0.23 | 1.01 (0.83-1.24) | 0.915 | 0.978 |
| rs2706347 | 5 | 131905117 | *RAD50* | A | C | 0.22 | 0.22 | 0.99 (0.81-1.21) | 0.916 | 0.978 |
| rs405684 | 17 | 56777148 | *RAD51C* | A | G | 0.38 | 0.39 | 1.01 (0.86-1.19) | 0.922 | 0.980 |
| rs1760904 | 14 | 20852029 | *TEP1* | G | A | 0.48 | 0.48 | 0.99 (0.85-1.16) | 0.932 | 0.985 |
| rs2897443 | 5 | 131929594 | *RAD50* | A | C | 0.22 | 0.21 | 1.01 (0.83-1.23) | 0.935 | 0.985 |
| rs1713456 | 14 | 20850093 | *TEP1* | A | G | 0.19 | 0.19 | 1.01 (0.82-1.24) | 0.948 | 0.985 |
| rs917855 | 12 | 21630146 | *RECQL* | A | G | 0.50 | 0.49 | 1.01 (0.85-1.19) | 0.951 | 0.985 |
| rs1551655 | 8 | 48873261 | *LOC106903146* | C | A | 0.08 | 0.08 | 1.01 (0.74-1.37) | 0.952 | 0.985 |
| rs2074369 | 5 | 131973663 | *TH2-LCR*, *RAD50*, *TH2LCRR* | G | A | 0.23 | 0.23 | 0.99 (0.81-1.22) | 0.954 | 0.985 |
| rs1772188 | 10 | 93616628 | *TNKS2* | A | C | 0.15 | 0.14 | 0.99 (0.78-1.26) | 0.957 | 0.985 |
| rs3219123 | 1 | 226555348 | *PARP1* | A | G | 0.05 | 0.05 | 1.01 (0.70-1.45) | 0.960 | 0.985 |
| rs9649886 | 8 | 30962693 | *WRN* | C | A | 0.38 | 0.38 | 1.00 (0.85-1.18) | 0.984 | 0.997 |
| rs2508678 | 11 | 94149349 | *MRE11* | A | G | 0.37 | 0.37 | 1.00 (0.84-1.19) | 0.987 | 0.997 |
| rs12334811 | 8 | 48832977 | *PRKDC* | A | G | 0.07 | 0.07 | 1.00 (0.73-1.37) | 0.987 | 0.997 |
| rs1341263 | 10 | 93556855 | *TNKS2-AS1* | G | A | 0.39 | 0.38 | 1.00 (0.85-1.18) | 0.992 | 0.997 |
| rs2228035 | 14 | 20871973 | *TEP1* | G | A | 0.05 | 0.05 | 1.00 (0.68-1.48) | 0.993 | 0.997 |
| rs2040704 | 5 | 131973177 | *TH2-LCR*, *RAD50*, *TH2LCRR* | G | A | 0.23 | 0.23 | 1.00 (0.82-1.22) | 1.000 | 1.000 |

Chromosomal positions are based on the Genome Reference Consortium Human Build 37 (GRCh37) of the single nucleotide polymorphism database (dbSNP) build 151, MAF; Minor allele frequency, FDR; False discovery rate.

**Supplementary Table S6**: Assessment of 236 SNPs associated with the telomere length with risk for GADA-first. Risk was estimated as Hazard ratios and 95% confidence intervals.

| **SNP IDs** | **Chromosome** | **Position** | **Gene** | **Minor allele** | **Major allele** | **MAF in cases** | **MAF in controls** | **HR (95% CI)** | ***P*-value** | **FDR adjusted *P*-value** |
| --- | --- | --- | --- | --- | --- | --- | --- | --- | --- | --- |
| rs7167216 | 15 | 91354521 | *BLM* | A | G | 0.10 | 0.08 | 1.32 (1.04-1.67) | 0.022 | 0.837 |
| rs609261 | 11 | 108158134 | *ATM* | A | G | 0.41 | 0.44 | 0.84 (0.72-0.98) | 0.023 | 0.837 |
| rs599558 | 11 | 108177538 | *ATM* | G | A | 0.41 | 0.44 | 0.84 (0.73-0.98) | 0.024 | 0.837 |
| rs4585 | 11 | 108239628 | *ATM*, *C11orf65* | C | A | 0.41 | 0.44 | 0.84 (0.73-0.98) | 0.025 | 0.837 |
| rs645485 | 11 | 108168863 | *ATM* | A | G | 0.41 | 0.44 | 0.85 (0.73-0.98) | 0.027 | 0.837 |
| rs4244612 | 8 | 145741702 | *RECQL4* | C | G | 0.36 | 0.41 | 0.84 (0.73-0.98) | 0.027 | 0.837 |
| rs2227935 | 15 | 91326099 | *BLM* | A | G | 0.09 | 0.07 | 1.32 (1.03-1.68) | 0.029 | 0.837 |
| rs600931 | 11 | 108117335 | *ATM* | G | A | 0.41 | 0.44 | 0.85 (0.73-0.98) | 0.030 | 0.837 |
| rs619972 | 11 | 108169619 | *ATM* | G | A | 0.42 | 0.44 | 0.85 (0.73-0.99) | 0.034 | 0.837 |
| rs228591 | 11 | 108097333 | *ATM* | A | G | 0.41 | 0.44 | 0.85 (0.74-0.99) | 0.038 | 0.837 |
| rs664143 | 11 | 108225661 | *ATM*, *C11orf65* | A | G | 0.40 | 0.43 | 0.85 (0.73-0.99) | 0.039 | 0.837 |
| rs623860 | 11 | 108106782 | *ATM* | G | A | 0.40 | 0.43 | 0.86 (0.74-1.00) | 0.044 | 0.853 |
| rs425538 | 11 | 108219339 | *ATM*, *C11orf65* | C | A | 0.40 | 0.43 | 0.86 (0.74-1.00) | 0.047 | 0.853 |
| rs12567614 | 1 | 226544420 | - | A | G | 0.49 | 0.46 | 1.16 (1.00-1.34) | 0.052 | 0.877 |
| rs9939870 | 16 | 69396585 | *TERF2* | A | G | 0.28 | 0.25 | 1.17 (1.00-1.36) | 0.057 | 0.897 |
| rs16943176 | 17 | 56769887 | *RAD51C* | A | G | 0.25 | 0.22 | 1.16 (0.99-1.37) | 0.076 | 0.911 |
| rs7184015 | 15 | 91274575 | *BLM* | A | C | 0.26 | 0.28 | 0.86 (0.73-1.02) | 0.082 | 0.911 |
| rs2104978 | 14 | 20837033 | *TEP1* | G | A | 0.08 | 0.06 | 1.26 (0.97-1.65) | 0.084 | 0.911 |
| rs12638862 | 3 | 169477506 | - | G | A | 0.24 | 0.28 | 0.86 (0.72-1.02) | 0.084 | 0.911 |
| rs13447720 | 11 | 94165326 | *MRE11* | G | A | 0.25 | 0.22 | 1.16 (0.98-1.37) | 0.085 | 0.911 |
| rs1805414 | 1 | 226573364 | *PARP1* | G | A | 0.39 | 0.37 | 1.14 (0.98-1.33) | 0.085 | 0.911 |
| rs2228041 | 14 | 20852267 | *TEP1* | A | G | 0.07 | 0.06 | 1.28 (0.97-1.69) | 0.086 | 0.911 |
| rs11160462 | 14 | 20868751 | *TEP1* | A | G | 0.34 | 0.37 | 0.87 (0.75-1.02) | 0.090 | 0.911 |
| rs2192170 | 12 | 21633036 | *RECQL* | A | G | 0.11 | 0.09 | 1.22 (0.96-1.54) | 0.099 | 0.911 |
| rs3219090 | 1 | 226564691 | *PARP1* | A | G | 0.39 | 0.37 | 1.13 (0.97-1.32) | 0.110 | 0.911 |
| rs2853672 | 5 | 1292983 | *TERT* | A | C | 0.47 | 0.50 | 1.13 (0.97-1.30) | 0.111 | 0.911 |
| rs17711777 | 8 | 10623240 | *PINX1*, *LOC102723313* | G | A | 0.06 | 0.08 | 0.81 (0.61-1.08) | 0.151 | 0.911 |
| rs8022805 | 14 | 20846950 | *TEP1* | A | G | 0.06 | 0.05 | 1.23 (0.93-1.64) | 0.152 | 0.911 |
| rs12630450 | 3 | 169480204 | - | G | A | 0.26 | 0.30 | 0.89 (0.75-1.05) | 0.166 | 0.911 |
| rs9822885 | 3 | 169486144 | *ACTRT3* | G | A | 0.26 | 0.29 | 0.89 (0.75-1.05) | 0.166 | 0.911 |
| rs11998382 | 8 | 10659517 | *PINX1* | G | A | 0.24 | 0.22 | 1.13 (0.95-1.34) | 0.171 | 0.911 |
| rs2508678 | 11 | 94149349 | *MRE11* | A | G | 0.35 | 0.37 | 0.90 (0.77-1.05) | 0.178 | 0.911 |
| rs12334407 | 8 | 73953688 | *TERF1* | A | C | 0.29 | 0.32 | 0.90 (0.77-1.05) | 0.179 | 0.911 |
| rs3102095 | 8 | 73916745 | - | C | A | 0.29 | 0.32 | 0.90 (0.77-1.05) | 0.180 | 0.911 |
| rs405684 | 17 | 56777148 | *RAD51C* | A | G | 0.36 | 0.39 | 0.90 (0.78-1.05) | 0.180 | 0.911 |
| rs740059 | 12 | 4671490 | *LOC113939935*, *DYRK4* | G | A | 0.50 | 0.49 | 1.10 (0.96-1.27) | 0.181 | 0.911 |
| rs10464529 | 7 | 124520308 | *POT1* | A | G | 0.28 | 0.26 | 1.12 (0.95-1.31) | 0.184 | 0.911 |
| rs7072165 | 10 | 93565641 | *TNKS2* | G | A | 0.09 | 0.11 | 0.85 (0.67-1.08) | 0.188 | 0.911 |
| rs2975852 | 8 | 73932430 | *TERF1* | A | G | 0.30 | 0.34 | 0.90 (0.77-1.05) | 0.189 | 0.911 |
| rs2228035 | 14 | 20871973 | *TEP1* | G | A | 0.04 | 0.05 | 0.78 (0.54-1.13) | 0.193 | 0.911 |
| rs6601530 | 8 | 10671272 | *PINX1* | G | A | 0.45 | 0.47 | 0.91 (0.79-1.05) | 0.207 | 0.911 |
| rs3219095 | 1 | 226563882 | *PARP1* | G | A | 0.14 | 0.15 | 0.88 (0.72-1.08) | 0.211 | 0.911 |
| rs2242652 | 5 | 1280028 | *TERT* | A | G | 0.19 | 0.21 | 0.89 (0.74-1.07) | 0.217 | 0.911 |
| rs1871892 | 17 | 33448818 | *FNDC8* | A | G | 0.31 | 0.32 | 0.90 (0.77-1.06) | 0.222 | 0.911 |
| rs2293464 | 1 | 226576296 | *PARP1* | A | G | 0.18 | 0.16 | 1.12 (0.93-1.35) | 0.232 | 0.911 |
| rs59672299 | 1 | 226597593 | - | G | A | 0.18 | 0.16 | 1.12 (0.93-1.35) | 0.233 | 0.911 |
| rs1000033 | 1 | 226580387 | *PARP1* | C | A | 0.18 | 0.16 | 1.12 (0.93-1.35) | 0.236 | 0.911 |
| rs2945461 | 14 | 20863070 | *TEP1* | G | A | 0.08 | 0.10 | 0.85 (0.65-1.11) | 0.238 | 0.911 |
| rs2853676 | 5 | 1288547 | *TERT* | A | G | 0.23 | 0.25 | 0.90 (0.76-1.07) | 0.241 | 0.911 |
| rs10492117 | 12 | 21659368 | *GOLT1B* | G | A | 0.48 | 0.50 | 0.91 (0.78-1.06) | 0.245 | 0.911 |
| rs820210 | 17 | 73650495 | *LOC107985013*, *RECQL5* | G | A | 0.33 | 0.35 | 0.91 (0.78-1.07) | 0.250 | 0.911 |
| rs976016 | 2 | 15772865 | - | G | A | 0.19 | 0.17 | 1.12 (0.92-1.35) | 0.264 | 0.911 |
| rs820196 | 17 | 73627539 | *RECQL5* | G | A | 0.21 | 0.23 | 0.90 (0.75-1.08) | 0.265 | 0.911 |
| rs820206 | 17 | 73641714 | *RECQL5* | A | G | 0.33 | 0.35 | 0.91 (0.78-1.07) | 0.268 | 0.911 |
| rs414634 | 15 | 91356253 | *BLM* | A | C | 0.26 | 0.28 | 0.92 (0.78-1.07) | 0.273 | 0.911 |
| rs1346044 | 8 | 31024654 | *WRN* | G | A | 0.24 | 0.26 | 0.91 (0.77-1.08) | 0.275 | 0.911 |
| rs4796033 | 17 | 33433487 | *RAD51D*, *RAD51L3-RFFL* | A | G | 0.14 | 0.15 | 0.89 (0.72-1.10) | 0.275 | 0.911 |
| rs2158177 | 5 | 131984058 | *TH2LCRR* | G | A | 0.22 | 0.21 | 1.10 (0.93-1.30) | 0.279 | 0.911 |
| rs75245322 | 5 | 131903026 | *RAD50* | C | A | 0.09 | 0.08 | 1.15 (0.89-1.49) | 0.285 | 0.911 |
| rs1063053 | 8 | 90947537 | *NBN*, *OSGIN2* | A | G | 0.31 | 0.32 | 0.92 (0.78-1.08) | 0.285 | 0.911 |
| rs10954778 | 8 | 31008747 | *WRN* | G | A | 0.33 | 0.31 | 1.09 (0.93-1.26) | 0.292 | 0.911 |
| rs16944894 | 15 | 91363303 | - | G | A | 0.21 | 0.19 | 1.10 (0.92-1.32) | 0.293 | 0.911 |
| rs3219125 | 1 | 226554951 | *PARP1* | G | A | 0.07 | 0.06 | 1.16 (0.88-1.54) | 0.295 | 0.911 |
| rs4809324 | 20 | 62318220 | *RTEL1*, *RTEL1-TNFRSF6B* | G | A | 0.09 | 0.11 | 0.88 (0.69-1.12) | 0.296 | 0.911 |
| rs6668851 | 1 | 226545866 | - | C | A | 0.31 | 0.30 | 1.09 (0.93-1.28) | 0.297 | 0.911 |
| rs733396 | 8 | 10621981 | *LOC102723313* | A | G | 0.46 | 0.44 | 1.08 (0.93-1.25) | 0.298 | 0.911 |
| rs9649886 | 8 | 30962693 | *WRN* | C | A | 0.40 | 0.38 | 1.08 (0.93-1.25) | 0.302 | 0.911 |
| rs6871536 | 5 | 131969874 | *TH2-LCR*, *RAD50*, *TH2LCRR* | G | A | 0.24 | 0.22 | 1.09 (0.92-1.29) | 0.307 | 0.911 |
| rs6011011 | 20 | 62299578 | *RTEL1*, *RTEL1-TNFRSF6B* | A | G | 0.06 | 0.08 | 0.86 (0.64-1.15) | 0.308 | 0.911 |
| rs1713456 | 14 | 20850093 | *TEP1* | A | G | 0.21 | 0.19 | 1.09 (0.92-1.30) | 0.309 | 0.911 |
| rs2736122 | 5 | 1257621 | *TERT* | A | G | 0.23 | 0.24 | 0.92 (0.77-1.09) | 0.316 | 0.911 |
| rs2301713 | 5 | 131951996 | *RAD50* | G | A | 0.24 | 0.22 | 1.09 (0.92-1.29) | 0.321 | 0.911 |
| rs2107465 | 8 | 91001024 | - | A | G | 0.29 | 0.30 | 0.92 (0.78-1.09) | 0.330 | 0.911 |
| rs3755133 | 2 | 15731583 | - | A | G | 0.18 | 0.17 | 1.10 (0.91-1.34) | 0.330 | 0.911 |
| rs1801516 | 11 | 108175462 | *ATM* | A | G | 0.16 | 0.16 | 1.10 (0.91-1.34) | 0.334 | 0.911 |
| rs12653750 | 5 | 131971902 | *TH2-LCR*, *RAD50*, *TH2LCRR* | A | G | 0.23 | 0.22 | 1.09 (0.92-1.28) | 0.335 | 0.911 |
| rs3755132 | 2 | 15729820 | - | C | A | 0.18 | 0.16 | 1.10 (0.90-1.34) | 0.341 | 0.911 |
| rs4982038 | 14 | 20862542 | *TEP1* | A | G | 0.31 | 0.32 | 0.92 (0.78-1.09) | 0.342 | 0.911 |
| rs2975842 | 8 | 73925591 | *TERF1* | A | G | 0.42 | 0.45 | 0.93 (0.81-1.08) | 0.345 | 0.911 |
| rs756627 | 8 | 145737286 | *RECQL4* | A | G | 0.44 | 0.46 | 0.93 (0.81-1.08) | 0.347 | 0.911 |
| rs2897443 | 5 | 131929594 | *RAD50* | A | C | 0.22 | 0.21 | 1.08 (0.92-1.28) | 0.351 | 0.911 |
| rs2240032 | 5 | 131977127 | *TH2-LCR*, *RAD50*, *TH2LCRR* | A | G | 0.23 | 0.22 | 1.08 (0.91-1.28) | 0.359 | 0.911 |
| rs6596086 | 5 | 131952222 | *RAD50* | G | A | 0.24 | 0.23 | 1.08 (0.92-1.28) | 0.359 | 0.911 |
| rs3798134 | 5 | 131965179 | *TH2-LCR*, *RAD50* | A | G | 0.24 | 0.22 | 1.08 (0.91-1.28) | 0.365 | 0.911 |
| rs11776767 | 8 | 10683929 | *PINX1* | C | G | 0.33 | 0.34 | 0.93 (0.80-1.09) | 0.369 | 0.911 |
| rs7299185 | 12 | 4672394 | *DYRK4* | C | A | 0.25 | 0.25 | 0.93 (0.79-1.10) | 0.380 | 0.911 |
| rs9282580 | 1 | 226594372 | *PARP1* | A | G | 0.05 | 0.05 | 0.86 (0.62-1.20) | 0.381 | 0.911 |
| rs4987034 | 8 | 30977696 | *WRN* | G | A | 0.17 | 0.18 | 0.92 (0.76-1.11) | 0.383 | 0.911 |
| rs10843881 | 12 | 31241345 | *DDX11* | G | A | 0.47 | 0.45 | 1.07 (0.92-1.24) | 0.389 | 0.911 |
| rs2074369 | 5 | 131973663 | *TH2-LCR*, *RAD50*, *TH2LCRR* | G | A | 0.24 | 0.23 | 1.08 (0.91-1.27) | 0.397 | 0.911 |
| rs10090277 | 8 | 9450886 | *TNKS* | G | A | 0.07 | 0.08 | 0.89 (0.67-1.18) | 0.401 | 0.911 |
| rs2291207 | 7 | 124569382 | *POT1* | G | A | 0.30 | 0.32 | 0.93 (0.79-1.10) | 0.402 | 0.911 |
| rs1805812 | 8 | 90965053 | *NBN* | G | A | 0.09 | 0.09 | 0.90 (0.69-1.16) | 0.403 | 0.911 |
| rs628728 | 11 | 94250379 | *C11orf97* | A | G | 0.40 | 0.42 | 0.94 (0.81-1.09) | 0.406 | 0.911 |
| rs604845 | 11 | 94182689 | *MRE11* | A | G | 0.32 | 0.34 | 0.93 (0.79-1.10) | 0.408 | 0.911 |
| rs2736100 | 5 | 1286516 | *TERT* | C | A | 0.48 | 0.50 | 1.06 (0.92-1.23) | 0.409 | 0.911 |
| rs114939615 | 1 | 226591587 | *PARP1* | A | G | 0.07 | 0.07 | 1.13 (0.85-1.51) | 0.411 | 0.911 |
| rs3848668 | 20 | 62293272 | *RTEL1*, *RTEL1-TNFRSF6B* | G | A | 0.06 | 0.07 | 0.88 (0.65-1.19) | 0.412 | 0.911 |
| rs12679892 | 8 | 9445429 | *TNKS* | G | A | 0.32 | 0.31 | 1.07 (0.91-1.24) | 0.414 | 0.911 |
| rs2297434 | 20 | 62294015 | *RTEL1*, *RTEL1-TNFRSF6B* | G | A | 0.47 | 0.48 | 0.94 (0.81-1.09) | 0.415 | 0.911 |
| rs3213212 | 12 | 21635232 | *RECQL* | A | G | 0.46 | 0.45 | 1.07 (0.91-1.24) | 0.419 | 0.911 |
| rs12706627 | 7 | 124550133 | *POT1* | G | A | 0.29 | 0.31 | 0.94 (0.80-1.10) | 0.422 | 0.911 |
| rs11250077 | 8 | 10656437 | *PINX1* | A | G | 0.41 | 0.42 | 0.94 (0.82-1.09) | 0.423 | 0.911 |
| rs663530 | 11 | 94157114 | *MRE11* | A | G | 0.24 | 0.26 | 0.94 (0.79-1.10) | 0.429 | 0.911 |
| rs2040704 | 5 | 131973177 | *TH2-LCR*, *RAD50*, *TH2LCRR* | G | A | 0.24 | 0.23 | 1.07 (0.90-1.27) | 0.431 | 0.911 |
| rs17774023 | 8 | 10626333 | *PINX1*, *LOC102723313* | G | A | 0.31 | 0.30 | 1.07 (0.91-1.25) | 0.432 | 0.911 |
| rs7737470 | 5 | 131974063 | *TH2-LCR*, *RAD50*, *TH2LCRR* | T | A | 0.23 | 0.22 | 1.07 (0.90-1.26) | 0.436 | 0.911 |
| rs227060 | 11 | 108204881 | *ATM*, *C11orf65* | A | G | 0.33 | 0.32 | 1.06 (0.91-1.23) | 0.437 | 0.911 |
| rs61835377 | 1 | 226545854 | - | A | G | 0.19 | 0.19 | 0.93 (0.77-1.12) | 0.437 | 0.911 |
| rs936656 | 17 | 33433820 | *RAD51D*, *RAD51L3-RFFL* | G | A | 0.45 | 0.47 | 0.94 (0.80-1.10) | 0.441 | 0.911 |
| rs13265363 | 8 | 9422926 | *TNKS* | C | A | 0.13 | 0.14 | 0.92 (0.74-1.14) | 0.443 | 0.911 |
| rs2292370 | 8 | 10690319 | *PINX1* | A | G | 0.29 | 0.30 | 0.94 (0.81-1.10) | 0.445 | 0.911 |
| rs4733220 | 8 | 30900890 | *WRN* | A | G | 0.43 | 0.42 | 1.06 (0.91-1.23) | 0.446 | 0.911 |
| rs12680047 | 8 | 128758861 | - | G | A | 0.38 | 0.39 | 0.94 (0.81-1.10) | 0.465 | 0.911 |
| rs2244012 | 5 | 131901225 | *RAD50* | G | A | 0.24 | 0.23 | 1.06 (0.90-1.26) | 0.467 | 0.911 |
| rs7159947 | 14 | 20809491 | - | G | A | 0.37 | 0.36 | 1.06 (0.91-1.22) | 0.470 | 0.911 |
| rs11542623 | 1 | 46746164 | *LRRC41* | A | G | 0.12 | 0.11 | 1.09 (0.87-1.36) | 0.470 | 0.911 |
| rs12187537 | 5 | 131939904 | *RAD50* | C | A | 0.22 | 0.21 | 1.07 (0.90-1.26) | 0.472 | 0.911 |
| rs6982126 | 8 | 73939623 | *TERF1* | A | G | 0.25 | 0.23 | 1.07 (0.90-1.27) | 0.473 | 0.911 |
| rs1048771 | 1 | 46743900 | *LRRC41*, *RAD54L* | A | G | 0.12 | 0.11 | 1.09 (0.87-1.36) | 0.476 | 0.911 |
| rs3087409 | 8 | 30946403 | *WRN* | A | C | 0.07 | 0.06 | 1.11 (0.83-1.50) | 0.479 | 0.911 |
| rs78087823 | 1 | 226583026 | *PARP1* | A | G | 0.06 | 0.05 | 1.12 (0.82-1.52) | 0.482 | 0.911 |
| rs1341263 | 10 | 93556855 | *TNKS2-AS1* | G | A | 0.37 | 0.38 | 0.95 (0.82-1.10) | 0.486 | 0.911 |
| rs10099824 | 8 | 73940350 | *TERF1* | A | G | 0.47 | 0.45 | 1.05 (0.91-1.23) | 0.493 | 0.911 |
| rs7834823 | 8 | 9437352 | *TNKS* | A | C | 0.27 | 0.26 | 1.06 (0.90-1.24) | 0.497 | 0.911 |
| rs13276086 | 8 | 9578982 | *TNKS* | A | C | 0.38 | 0.39 | 0.95 (0.82-1.10) | 0.502 | 0.911 |
| rs11785739 | 8 | 9640400 | - | A | C | 0.07 | 0.08 | 0.91 (0.69-1.20) | 0.503 | 0.911 |
| rs6984094 | 8 | 10645738 | *PINX1* | G | A | 0.12 | 0.12 | 0.93 (0.75-1.16) | 0.511 | 0.911 |
| rs622961 | 11 | 94154902 | *MRE11* | A | G | 0.42 | 0.44 | 0.95 (0.82-1.10) | 0.512 | 0.911 |
| rs2230009 | 8 | 30921935 | *WRN* | A | G | 0.07 | 0.06 | 1.10 (0.82-1.48) | 0.513 | 0.911 |
| rs1800392 | 8 | 30973957 | *WRN* | A | C | 0.43 | 0.44 | 0.95 (0.83-1.10) | 0.519 | 0.911 |
| rs907190 | 1 | 226566726 | *PARP1* | A | C | 0.21 | 0.20 | 1.06 (0.88-1.29) | 0.521 | 0.911 |
| rs78733221 | 8 | 9476448 | *TNKS* | G | A | 0.07 | 0.08 | 0.91 (0.69-1.21) | 0.521 | 0.911 |
| rs10102170 | 8 | 9611470 | *TNKS* | A | G | 0.38 | 0.39 | 0.95 (0.82-1.10) | 0.528 | 0.911 |
| rs7170919 | 15 | 91314959 | *BLM* | A | G | 0.30 | 0.29 | 1.05 (0.90-1.23) | 0.530 | 0.911 |
| rs748883 | 1 | 226582325 | *PARP1* | G | A | 0.29 | 0.28 | 1.05 (0.90-1.24) | 0.531 | 0.911 |
| rs1136410 | 1 | 226555302 | *PARP1* | G | A | 0.21 | 0.20 | 1.06 (0.88-1.29) | 0.542 | 0.911 |
| rs12545912 | 8 | 9601699 | *TNKS* | C | A | 0.26 | 0.27 | 0.95 (0.81-1.12) | 0.545 | 0.911 |
| rs476137 | 11 | 94213905 | *MRE11* | A | C | 0.41 | 0.43 | 0.96 (0.83-1.11) | 0.547 | 0.911 |
| rs2706348 | 5 | 131905810 | *RAD50* | A | G | 0.23 | 0.22 | 1.05 (0.89-1.25) | 0.552 | 0.911 |
| rs2725385 | 8 | 30928146 | *WRN* | A | G | 0.29 | 0.28 | 1.05 (0.90-1.23) | 0.553 | 0.911 |
| rs7015700 | 8 | 9527707 | *TNKS* | A | G | 0.19 | 0.20 | 0.95 (0.79-1.14) | 0.561 | 0.911 |
| rs2853677 | 5 | 1287194 | *LOC110806264*, *TERT* | G | A | 0.42 | 0.41 | 1.04 (0.90-1.21) | 0.562 | 0.911 |
| rs4660918 | 1 | 46731694 | *RAD54L* | G | A | 0.25 | 0.26 | 0.95 (0.79-1.13) | 0.562 | 0.911 |
| rs11250076 | 8 | 10647823 | *PINX1* | A | G | 0.46 | 0.47 | 0.96 (0.83-1.11) | 0.566 | 0.911 |
| rs12334811 | 8 | 48832977 | *PRKDC* | A | G | 0.07 | 0.07 | 1.09 (0.81-1.45) | 0.581 | 0.911 |
| rs2898250 | 8 | 10621061 | *LOC102723313* | A | G | 0.12 | 0.12 | 0.94 (0.75-1.17) | 0.584 | 0.911 |
| rs2110159 | 12 | 21649160 | *RECQL* | A | G | 0.49 | 0.50 | 1.04 (0.90-1.21) | 0.585 | 0.911 |
| rs10101132 | 8 | 9616553 | *TNKS* | A | C | 0.32 | 0.32 | 0.96 (0.82-1.12) | 0.588 | 0.911 |
| rs12142240 | 1 | 46747301 | *LRRC41* | G | A | 0.32 | 0.30 | 1.04 (0.89-1.22) | 0.600 | 0.911 |
| rs33994795 | 8 | 9473429 | *TNKS* | G | A | 0.39 | 0.40 | 0.96 (0.84-1.11) | 0.601 | 0.911 |
| rs6994361 | 8 | 31018685 | *WRN* | G | A | 0.35 | 0.36 | 0.96 (0.83-1.12) | 0.603 | 0.911 |
| rs1944967 | 18 | 39531139 | - | G | A | 0.39 | 0.38 | 1.04 (0.90-1.21) | 0.605 | 0.911 |
| rs3219123 | 1 | 226555348 | *PARP1* | A | G | 0.06 | 0.05 | 1.09 (0.79-1.49) | 0.607 | 0.911 |
| rs2072352 | 15 | 91334179 | *BLM* | A | G | 0.28 | 0.29 | 0.96 (0.81-1.13) | 0.608 | 0.911 |
| rs17183344 | 15 | 91357488 | *BLM* | A | G | 0.19 | 0.18 | 1.05 (0.87-1.26) | 0.609 | 0.911 |
| rs1941526 | 18 | 39652939 | *PIK3C3* | A | G | 0.25 | 0.25 | 1.04 (0.89-1.23) | 0.610 | 0.911 |
| rs1801195 | 8 | 30999280 | *WRN* | A | C | 0.43 | 0.43 | 0.96 (0.84-1.11) | 0.616 | 0.911 |
| rs4353905 | 4 | 110745938 | - | G | A | 0.16 | 0.16 | 1.05 (0.86-1.29) | 0.617 | 0.911 |
| rs13273033 | 8 | 9540693 | *TNKS* | G | A | 0.26 | 0.27 | 0.96 (0.82-1.13) | 0.619 | 0.911 |
| rs11082257 | 18 | 39570683 | *PIK3C3* | G | A | 0.25 | 0.24 | 1.04 (0.88-1.23) | 0.622 | 0.911 |
| rs4246977 | 14 | 20882591 | - | G | A | 0.38 | 0.38 | 0.96 (0.83-1.12) | 0.630 | 0.911 |
| rs61909598 | 12 | 4671930 | *DYRK4* | A | C | 0.12 | 0.12 | 1.05 (0.85-1.31) | 0.634 | 0.911 |
| rs4463363 | 7 | 124513770 | *POT1* | A | G | 0.42 | 0.42 | 0.97 (0.84-1.12) | 0.640 | 0.911 |
| rs12125573 | 1 | 46734792 | *RAD54L* | G | A | 0.29 | 0.30 | 0.96 (0.82-1.13) | 0.642 | 0.911 |
| rs1713449 | 14 | 20841707 | *TEP1* | A | G | 0.22 | 0.24 | 0.96 (0.81-1.15) | 0.648 | 0.911 |
| rs2234744 | 8 | 90970935 | *NBN* | A | G | 0.32 | 0.33 | 0.96 (0.82-1.13) | 0.649 | 0.911 |
| rs1078543 | 8 | 10623138 | *PINX1*, *LOC102723313* | T | A | 0.12 | 0.12 | 0.95 (0.76-1.19) | 0.654 | 0.911 |
| rs1760898 | 14 | 20872881 | *TEP1* | A | C | 0.22 | 0.23 | 0.96 (0.79-1.16) | 0.658 | 0.911 |
| rs2706347 | 5 | 131905117 | *RAD50* | A | C | 0.23 | 0.22 | 1.04 (0.88-1.23) | 0.660 | 0.911 |
| rs2073635 | 8 | 90995366 | *NBN* | A | G | 0.33 | 0.33 | 0.97 (0.83-1.13) | 0.663 | 0.911 |
| rs1063045 | 8 | 90995019 | *NBN* | A | G | 0.33 | 0.33 | 0.97 (0.83-1.13) | 0.664 | 0.911 |
| rs962863 | 7 | 124558339 | *POT1* | A | G | 0.42 | 0.42 | 0.97 (0.84-1.12) | 0.674 | 0.911 |
| rs3219038 | 1 | 226578809 | *PARP1* | A | G | 0.11 | 0.11 | 0.95 (0.76-1.20) | 0.674 | 0.911 |
| rs7175811 | 15 | 91331546 | *BLM* | A | G | 0.37 | 0.36 | 1.03 (0.89-1.20) | 0.680 | 0.911 |
| rs1805818 | 8 | 90970862 | *NBN* | A | C | 0.33 | 0.33 | 0.97 (0.83-1.13) | 0.682 | 0.911 |
| rs3093933 | 14 | 20824415 | *PARP2* | A | C | 0.25 | 0.25 | 0.97 (0.82-1.14) | 0.683 | 0.911 |
| rs4668936 | 2 | 15731605 | - | G | A | 0.40 | 0.40 | 1.03 (0.89-1.19) | 0.695 | 0.915 |
| rs1805794 | 8 | 90990479 | *NBN* | C | G | 0.32 | 0.33 | 0.97 (0.83-1.13) | 0.696 | 0.915 |
| rs1760897 | 14 | 20876253 | *TEP1* | G | A | 0.30 | 0.31 | 0.97 (0.83-1.14) | 0.698 | 0.915 |
| rs7083959 | 10 | 93585997 | *TNKS2* | C | A | 0.18 | 0.17 | 1.04 (0.86-1.26) | 0.711 | 0.924 |
| rs9956832 | 18 | 39564876 | *PIK3C3* | A | C | 0.13 | 0.13 | 1.04 (0.84-1.29) | 0.718 | 0.924 |
| rs4549545 | 5 | 177584923 | - | G | A | 0.20 | 0.19 | 1.04 (0.86-1.25) | 0.721 | 0.924 |
| rs1713425 | 14 | 20858174 | *TEP1* | A | G | 0.45 | 0.44 | 1.03 (0.89-1.19) | 0.731 | 0.924 |
| rs3219142 | 1 | 226552068 | *PARP1* | A | G | 0.18 | 0.19 | 0.97 (0.80-1.17) | 0.731 | 0.924 |
| rs909341 | 20 | 62328742 | *TNFRSF6B*, *RTEL1-TNFRSF6B* | A | G | 0.22 | 0.22 | 0.97 (0.81-1.16) | 0.734 | 0.924 |
| rs6668722 | 1 | 226545776 | - | G | A | 0.10 | 0.10 | 1.04 (0.82-1.33) | 0.740 | 0.924 |
| rs17772565 | 5 | 131952405 | *RAD50* | A | G | 0.05 | 0.06 | 0.95 (0.68-1.32) | 0.742 | 0.924 |
| rs6470522 | 8 | 90954481 | *NBN* | A | G | 0.18 | 0.17 | 1.03 (0.85-1.25) | 0.749 | 0.924 |
| rs6601333 | 8 | 9427048 | *TNKS* | G | A | 0.26 | 0.26 | 1.03 (0.88-1.20) | 0.750 | 0.924 |
| rs8031341 | 15 | 91313224 | *BLM* | G | A | 0.20 | 0.21 | 0.97 (0.81-1.17) | 0.751 | 0.924 |
| rs444325 | 15 | 91364015 | - | A | C | 0.35 | 0.35 | 1.03 (0.88-1.20) | 0.752 | 0.924 |
| rs1551655 | 8 | 48873261 | *LOC106903146* | C | A | 0.08 | 0.08 | 1.05 (0.78-1.40) | 0.760 | 0.929 |
| rs1772188 | 10 | 93616628 | *TNKS2* | A | C | 0.15 | 0.14 | 1.03 (0.84-1.27) | 0.764 | 0.929 |
| rs538800 | 11 | 94239567 | *MRE11* | A | G | 0.33 | 0.33 | 0.98 (0.83-1.15) | 0.785 | 0.937 |
| rs1805796 | 8 | 90993395 | *NBN* | A | G | 0.38 | 0.39 | 0.98 (0.84-1.14) | 0.787 | 0.937 |
| rs11930711 | 4 | 110734498 | - | A | G | 0.09 | 0.09 | 1.03 (0.81-1.32) | 0.789 | 0.937 |
| rs2237060 | 5 | 131970885 | *TH2-LCR*, *RAD50*, *TH2LCRR* | C | A | 0.39 | 0.37 | 1.02 (0.88-1.19) | 0.789 | 0.937 |
| rs917855 | 12 | 21630146 | *RECQL* | A | G | 0.50 | 0.49 | 1.02 (0.88-1.19) | 0.790 | 0.937 |
| rs3784780 | 15 | 91309475 | *BLM* | A | G | 0.16 | 0.16 | 0.97 (0.80-1.19) | 0.797 | 0.938 |
| rs2297437 | 20 | 62305274 | *RTEL1*, *RTEL1-TNFRSF6B* | A | G | 0.19 | 0.20 | 0.98 (0.81-1.18) | 0.799 | 0.938 |
| rs11249930 | 8 | 9472445 | *TNKS* | G | A | 0.20 | 0.19 | 1.02 (0.85-1.22) | 0.809 | 0.945 |
| rs10849087 | 12 | 4650065 | *RAD51AP1* | A | G | 0.21 | 0.21 | 0.98 (0.83-1.16) | 0.819 | 0.952 |
| rs389480 | 15 | 91351930 | *BLM* | A | G | 0.46 | 0.46 | 0.98 (0.85-1.14) | 0.830 | 0.960 |
| rs2270132 | 15 | 91351868 | *BLM* | C | A | 0.42 | 0.41 | 1.02 (0.87-1.18) | 0.836 | 0.962 |
| rs2725344 | 8 | 30918098 | *WRN* | G | A | 0.07 | 0.08 | 0.97 (0.73-1.29) | 0.846 | 0.966 |
| rs17772583 | 5 | 131953510 | *RAD50* | G | A | 0.24 | 0.24 | 0.98 (0.82-1.17) | 0.851 | 0.966 |
| rs2238337 | 15 | 91355732 | *BLM* | A | G | 0.36 | 0.36 | 1.01 (0.87-1.18) | 0.857 | 0.966 |
| rs7838341 | 8 | 10661734 | *PINX1* | G | A | 0.23 | 0.23 | 1.02 (0.85-1.21) | 0.866 | 0.966 |
| rs2299014 | 5 | 131903399 | *RAD50* | C | A | 0.42 | 0.41 | 1.01 (0.87-1.17) | 0.869 | 0.966 |
| rs7162960 | 15 | 91287412 | *BLM* | G | A | 0.22 | 0.22 | 0.99 (0.83-1.18) | 0.871 | 0.966 |
| rs11249943 | 8 | 9607863 | *TNKS* | C | A | 0.21 | 0.21 | 0.99 (0.83-1.18) | 0.871 | 0.966 |
| rs304268 | 17 | 56799907 | *RAD51C* | G | A | 0.31 | 0.31 | 0.99 (0.85-1.15) | 0.872 | 0.966 |
| rs2227933 | 15 | 91337479 | *BLM* | A | G | 0.18 | 0.18 | 1.01 (0.84-1.23) | 0.883 | 0.974 |
| rs6601338 | 8 | 9438928 | *TNKS* | A | G | 0.38 | 0.39 | 0.99 (0.86-1.14) | 0.891 | 0.978 |
| rs1805793 | 8 | 90990091 | *NBN* | G | A | 0.39 | 0.39 | 0.99 (0.85-1.15) | 0.903 | 0.982 |
| rs1760904 | 14 | 20852029 | *TEP1* | G | A | 0.48 | 0.48 | 0.99 (0.86-1.15) | 0.904 | 0.982 |
| rs3017077 | 11 | 94175249 | *MRE11* | A | G | 0.34 | 0.34 | 1.01 (0.87-1.18) | 0.907 | 0.982 |
| rs709816 | 8 | 90967711 | *NBN* | G | A | 0.39 | 0.39 | 0.99 (0.85-1.16) | 0.914 | 0.983 |
| rs938886 | 14 | 20837701 | *TEP1* | G | C | 0.23 | 0.24 | 0.99 (0.83-1.18) | 0.916 | 0.983 |
| rs13250838 | 8 | 9526345 | *TNKS* | A | G | 0.12 | 0.12 | 0.99 (0.80-1.23) | 0.930 | 0.992 |
| rs3761124 | 20 | 62288752 | - | A | G | 0.22 | 0.21 | 1.01 (0.85-1.20) | 0.933 | 0.992 |
| rs6010620 | 20 | 62309839 | *RTEL1*, *RTEL1-TNFRSF6B* | A | G | 0.23 | 0.22 | 1.01 (0.85-1.19) | 0.952 | 0.994 |
| rs10069690 | 5 | 1279790 | *TERT* | A | G | 0.28 | 0.27 | 1.01 (0.85-1.19) | 0.954 | 0.994 |
| rs1882928 | 8 | 31023822 | *WRN* | G | A | 0.42 | 0.42 | 1.00 (0.86-1.15) | 0.957 | 0.994 |
| rs7150689 | 14 | 20842964 | *TEP1* | G | A | 0.24 | 0.25 | 1.00 (0.84-1.18) | 0.957 | 0.994 |
| rs3755130 | 2 | 15729583 | - | A | G | 0.42 | 0.42 | 1.00 (0.87-1.16) | 0.958 | 0.994 |
| rs7826180 | 8 | 10627844 | *PINX1*, *LOC102723313* | A | G | 0.22 | 0.22 | 1.01 (0.84-1.20) | 0.960 | 0.994 |
| rs7307064 | 12 | 21634394 | *RECQL* | C | A | 0.11 | 0.12 | 1.01 (0.80-1.26) | 0.965 | 0.994 |
| rs6601524 | 8 | 10635222 | *PINX1* | A | C | 0.22 | 0.22 | 1.00 (0.84-1.20) | 0.969 | 0.994 |
| rs1800391 | 8 | 30938704 | *WRN* | A | G | 0.08 | 0.08 | 1.00 (0.76-1.32) | 0.984 | 0.999 |
| rs4427176 | 8 | 9533399 | *TNKS* | A | C | 0.19 | 0.19 | 1.00 (0.84-1.20) | 0.986 | 0.999 |
| rs1760903 | 14 | 20852817 | *TEP1* | A | G | 0.46 | 0.46 | 1.00 (0.86-1.16) | 0.990 | 0.999 |
| rs11211262 | 1 | 46721155 | *RAD54L* | A | G | 0.41 | 0.40 | 1.00 (0.86-1.17) | 0.992 | 0.999 |
| rs7302664 | 12 | 4664821 | *RAD51AP1* | A | G | 0.10 | 0.10 | 1.00 (0.79-1.26) | 0.998 | 0.999 |
| rs654718 | 11 | 94190115 | *MRE11* | G | A | 0.34 | 0.34 | 1.00 (0.86-1.17) | 0.999 | 0.999 |

Chromosomal positions are based on the Genome Reference Consortium Human Build 37 (GRCh37) of the single nucleotide polymorphism database (dbSNP) build 151, MAF; Minor allele frequency, FDR; False discovery rate.

**Supplementary Table S7**: Assessment of 236 SNPs associated with the telomere length with risk for T1D. Risk was estimated as Hazard ratios and 95% confidence intervals.

| **SNP IDs** | **Chromosome** | **Position** | **Gene** | **Minor allele** | **Major allele** | **MAF in cases** | **MAF in controls** | **HR (95% CI)** | ***P*-value** | **FDR adjusted *P*-value** |
| --- | --- | --- | --- | --- | --- | --- | --- | --- | --- | --- |
| rs61909598 | 12 | 4671930 | *DYRK4* | A | C | 0.15 | 0.12 | 1.30 (1.06-1.60) | 0.013 | 0.997 |
| rs75245322 | 5 | 131903026 | *RAD50* | C | A | 0.10 | 0.08 | 1.36 (1.05-1.76) | 0.018 | 0.997 |
| rs11082257 | 18 | 39570683 | *PIK3C3* | G | A | 0.20 | 0.24 | 0.79 (0.65-0.97) | 0.022 | 0.997 |
| rs7167216 | 15 | 91354521 | *BLM* | A | G | 0.10 | 0.08 | 1.30 (1.02-1.66) | 0.038 | 0.997 |
| rs2725344 | 8 | 30918098 | *WRN* | G | A | 0.06 | 0.08 | 0.71 (0.51-1.00) | 0.050 | 0.997 |
| rs414634 | 15 | 91356253 | *BLM* | A | C | 0.25 | 0.28 | 0.84 (0.71-1.01) | 0.058 | 0.997 |
| rs733396 | 8 | 10621981 | *LOC102723313* | A | G | 0.49 | 0.44 | 1.16 (0.99-1.36) | 0.061 | 0.997 |
| rs1941526 | 18 | 39652939 | *PIK3C3* | A | G | 0.21 | 0.25 | 0.83 (0.69-1.01) | 0.063 | 0.997 |
| rs1800392 | 8 | 30973957 | *WRN* | A | C | 0.40 | 0.44 | 0.86 (0.74-1.01) | 0.063 | 0.997 |
| rs9282580 | 1 | 226594372 | *PARP1* | A | G | 0.03 | 0.05 | 0.67 (0.44-1.02) | 0.063 | 0.997 |
| rs16944894 | 15 | 91363303 | - | G | A | 0.22 | 0.19 | 1.19 (0.99-1.42) | 0.067 | 0.997 |
| rs2227935 | 15 | 91326099 | *BLM* | A | G | 0.09 | 0.07 | 1.28 (0.98-1.67) | 0.076 | 0.997 |
| rs4463363 | 7 | 124513770 | *POT1* | A | G | 0.39 | 0.43 | 0.87 (0.75-1.02) | 0.077 | 0.997 |
| rs962863 | 7 | 124558339 | *POT1* | A | G | 0.39 | 0.42 | 0.87 (0.75-1.02) | 0.082 | 0.997 |
| rs907190 | 1 | 226566726 | *PARP1* | A | C | 0.17 | 0.21 | 0.84 (0.68-1.03) | 0.088 | 0.997 |
| rs1801195 | 8 | 30999280 | *WRN* | A | C | 0.40 | 0.43 | 0.87 (0.75-1.02) | 0.088 | 0.997 |
| rs1944967 | 18 | 39531139 | - | G | A | 0.35 | 0.39 | 0.87 (0.74-1.02) | 0.090 | 0.997 |
| rs78087823 | 1 | 226583026 | *PARP1* | A | G | 0.07 | 0.05 | 1.30 (0.96-1.76) | 0.090 | 0.997 |
| rs1136410 | 1 | 226555302 | *PARP1* | G | A | 0.17 | 0.20 | 0.84 (0.69-1.04) | 0.104 | 0.997 |
| rs12334811 | 8 | 48832977 | *PRKDC* | A | G | 0.05 | 0.07 | 0.76 (0.54-1.06) | 0.107 | 0.997 |
| rs6601530 | 8 | 10671272 | *PINX1* | G | A | 0.44 | 0.47 | 0.88 (0.75-1.03) | 0.113 | 0.997 |
| rs7175811 | 15 | 91331546 | *BLM* | A | G | 0.40 | 0.36 | 1.13 (0.97-1.32) | 0.120 | 0.997 |
| rs7299185 | 12 | 4672394 | *DYRK4* | C | A | 0.23 | 0.25 | 0.87 (0.73-1.04) | 0.136 | 0.997 |
| rs2293464 | 1 | 226576296 | *PARP1* | A | G | 0.18 | 0.16 | 1.16 (0.95-1.42) | 0.139 | 0.997 |
| rs59672299 | 1 | 226597593 | - | G | A | 0.18 | 0.16 | 1.16 (0.95-1.42) | 0.139 | 0.997 |
| rs3219123 | 1 | 226555348 | *PARP1* | A | G | 0.06 | 0.05 | 1.26 (0.93-1.72) | 0.139 | 0.997 |
| rs1000033 | 1 | 226580387 | *PARP1* | C | A | 0.18 | 0.16 | 1.16 (0.95-1.42) | 0.141 | 0.997 |
| rs4982038 | 14 | 20862542 | *TEP1* | A | G | 0.29 | 0.32 | 0.89 (0.76-1.05) | 0.156 | 0.997 |
| rs7170919 | 15 | 91314959 | *BLM* | A | G | 0.32 | 0.29 | 1.12 (0.95-1.33) | 0.163 | 0.997 |
| rs7184015 | 15 | 91274575 | *BLM* | A | C | 0.26 | 0.28 | 0.88 (0.74-1.05) | 0.166 | 0.997 |
| rs7302664 | 12 | 4664821 | *RAD51AP1* | A | G | 0.12 | 0.10 | 1.17 (0.93-1.47) | 0.174 | 0.997 |
| rs17774023 | 8 | 10626333 | *PINX1*, *LOC102723313* | G | A | 0.33 | 0.29 | 1.12 (0.95-1.31) | 0.177 | 0.997 |
| rs11998382 | 8 | 10659517 | *PINX1* | G | A | 0.25 | 0.22 | 1.13 (0.95-1.35) | 0.177 | 0.997 |
| rs12706627 | 7 | 124550133 | *POT1* | G | A | 0.33 | 0.31 | 1.12 (0.95-1.31) | 0.180 | 0.997 |
| rs10101132 | 8 | 9616553 | *TNKS* | A | C | 0.30 | 0.32 | 0.90 (0.76-1.05) | 0.182 | 0.997 |
| rs6984094 | 8 | 10645738 | *PINX1* | G | A | 0.10 | 0.12 | 0.85 (0.67-1.08) | 0.182 | 0.997 |
| rs6668722 | 1 | 226545776 | - | G | A | 0.12 | 0.10 | 1.18 (0.93-1.50) | 0.184 | 0.997 |
| rs2228035 | 14 | 20871973 | *TEP1* | G | A | 0.03 | 0.05 | 0.76 (0.51-1.15) | 0.190 | 0.997 |
| rs2242652 | 5 | 1280028 | *TERT* | A | G | 0.19 | 0.21 | 0.88 (0.72-1.07) | 0.193 | 0.997 |
| rs4244612 | 8 | 145741702 | *RECQL4* | C | G | 0.38 | 0.41 | 0.90 (0.77-1.06) | 0.197 | 0.997 |
| rs4549545 | 5 | 177584923 | - | G | A | 0.17 | 0.19 | 0.87 (0.70-1.08) | 0.211 | 0.997 |
| rs4353905 | 4 | 110745938 | - | G | A | 0.16 | 0.16 | 1.14 (0.93-1.39) | 0.221 | 0.997 |
| rs3087409 | 8 | 30946403 | *WRN* | A | C | 0.07 | 0.06 | 1.19 (0.90-1.59) | 0.228 | 0.997 |
| rs2291207 | 7 | 124569382 | *POT1* | G | A | 0.33 | 0.31 | 1.10 (0.94-1.30) | 0.229 | 0.997 |
| rs6994361 | 8 | 31018685 | *WRN* | G | A | 0.33 | 0.36 | 0.91 (0.77-1.07) | 0.235 | 0.997 |
| rs2230009 | 8 | 30921935 | *WRN* | A | G | 0.07 | 0.06 | 1.19 (0.89-1.58) | 0.247 | 0.997 |
| rs3219095 | 1 | 226563882 | *PARP1* | G | A | 0.14 | 0.15 | 0.88 (0.72-1.09) | 0.251 | 0.997 |
| rs936656 | 17 | 33433820 | *RAD51D*, *RAD51L3-RFFL* | G | A | 0.44 | 0.47 | 0.92 (0.78-1.07) | 0.275 | 0.997 |
| rs1078543 | 8 | 10623138 | *PINX1*, *LOC102723313* | T | A | 0.11 | 0.12 | 0.88 (0.69-1.12) | 0.284 | 0.997 |
| rs10069690 | 5 | 1279790 | *TERT* | A | G | 0.26 | 0.27 | 0.91 (0.76-1.08) | 0.288 | 0.997 |
| rs2898250 | 8 | 10621061 | *LOC102723313* | A | G | 0.11 | 0.12 | 0.88 (0.69-1.12) | 0.288 | 0.997 |
| rs1871892 | 17 | 33448818 | *FNDC8* | A | G | 0.31 | 0.32 | 0.92 (0.78-1.08) | 0.293 | 0.997 |
| rs1551655 | 8 | 48873261 | *LOC106903146* | C | A | 0.06 | 0.08 | 0.85 (0.62-1.16) | 0.297 | 0.997 |
| rs13265363 | 8 | 9422926 | *TNKS* | C | A | 0.12 | 0.14 | 0.88 (0.70-1.12) | 0.301 | 0.997 |
| rs748883 | 1 | 226582325 | *PARP1* | G | A | 0.29 | 0.28 | 1.09 (0.92-1.29) | 0.306 | 0.997 |
| rs1760904 | 14 | 20852029 | *TEP1* | G | A | 0.46 | 0.49 | 0.92 (0.79-1.08) | 0.306 | 0.997 |
| rs11160462 | 14 | 20868751 | *TEP1* | A | G | 0.35 | 0.37 | 0.92 (0.79-1.08) | 0.309 | 0.997 |
| rs9649886 | 8 | 30962693 | *WRN* | C | A | 0.40 | 0.38 | 1.09 (0.93-1.27) | 0.309 | 0.997 |
| rs6601524 | 8 | 10635222 | *PINX1* | A | C | 0.23 | 0.22 | 1.10 (0.91-1.33) | 0.309 | 0.997 |
| rs1063053 | 8 | 90947537 | *NBN*, *OSGIN2* | A | G | 0.34 | 0.32 | 1.09 (0.93-1.27) | 0.313 | 0.997 |
| rs10843881 | 12 | 31241345 | *DDX11* | G | A | 0.43 | 0.46 | 0.92 (0.79-1.08) | 0.318 | 0.997 |
| rs7838341 | 8 | 10661734 | *PINX1* | G | A | 0.24 | 0.23 | 1.10 (0.91-1.32) | 0.326 | 0.997 |
| rs304268 | 17 | 56799907 | *RAD51C* | G | A | 0.33 | 0.31 | 1.08 (0.93-1.27) | 0.326 | 0.997 |
| rs4987034 | 8 | 30977696 | *WRN* | G | A | 0.20 | 0.18 | 1.10 (0.91-1.33) | 0.329 | 0.997 |
| rs11250077 | 8 | 10656437 | *PINX1* | A | G | 0.40 | 0.42 | 0.92 (0.79-1.09) | 0.338 | 0.997 |
| rs13250838 | 8 | 9526345 | *TNKS* | A | G | 0.11 | 0.13 | 0.89 (0.70-1.13) | 0.338 | 0.997 |
| rs628728 | 11 | 94250379 | *C11orf97* | A | G | 0.40 | 0.42 | 0.93 (0.80-1.08) | 0.339 | 0.997 |
| rs2853676 | 5 | 1288547 | *TERT* | A | G | 0.23 | 0.25 | 0.92 (0.76-1.10) | 0.339 | 0.997 |
| rs2945461 | 14 | 20863070 | *TEP1* | G | A | 0.10 | 0.10 | 1.13 (0.88-1.47) | 0.346 | 0.997 |
| rs11249943 | 8 | 9607863 | *TNKS* | C | A | 0.20 | 0.21 | 0.91 (0.74-1.11) | 0.348 | 0.997 |
| rs756627 | 8 | 145737286 | *RECQL4* | A | G | 0.44 | 0.46 | 0.93 (0.80-1.08) | 0.349 | 0.997 |
| rs7150689 | 14 | 20842964 | *TEP1* | G | A | 0.23 | 0.25 | 0.92 (0.76-1.10) | 0.362 | 0.997 |
| rs17772583 | 5 | 131953510 | *RAD50* | G | A | 0.25 | 0.24 | 1.08 (0.91-1.29) | 0.369 | 0.997 |
| rs13276086 | 8 | 9578982 | *TNKS* | A | C | 0.37 | 0.39 | 0.93 (0.80-1.09) | 0.371 | 0.997 |
| rs7307064 | 12 | 21634394 | *RECQL* | C | A | 0.13 | 0.11 | 1.11 (0.89-1.38) | 0.376 | 0.997 |
| rs2072352 | 15 | 91334179 | *BLM* | A | G | 0.31 | 0.29 | 1.07 (0.91-1.26) | 0.392 | 0.997 |
| rs2270132 | 15 | 91351868 | *BLM* | C | A | 0.43 | 0.41 | 1.07 (0.92-1.25) | 0.395 | 0.997 |
| rs4668936 | 2 | 15731605 | - | G | A | 0.42 | 0.40 | 1.07 (0.92-1.24) | 0.397 | 0.997 |
| rs4660918 | 1 | 46731694 | *RAD54L* | G | A | 0.24 | 0.26 | 0.93 (0.78-1.10) | 0.406 | 0.997 |
| rs12142240 | 1 | 46747301 | *LRRC41* | G | A | 0.32 | 0.30 | 1.07 (0.91-1.25) | 0.410 | 0.997 |
| rs820196 | 17 | 73627539 | *RECQL5* | G | A | 0.24 | 0.23 | 1.08 (0.90-1.29) | 0.410 | 0.997 |
| rs4796033 | 17 | 33433487 | *RAD51D*, *RAD51L3-RFFL* | A | G | 0.14 | 0.15 | 0.91 (0.74-1.13) | 0.412 | 0.997 |
| rs17183344 | 15 | 91357488 | *BLM* | A | G | 0.19 | 0.18 | 1.08 (0.90-1.31) | 0.419 | 0.997 |
| rs7072165 | 10 | 93565641 | *TNKS2* | G | A | 0.09 | 0.11 | 0.90 (0.70-1.16) | 0.420 | 0.997 |
| rs1713425 | 14 | 20858174 | *TEP1* | A | G | 0.45 | 0.44 | 1.06 (0.91-1.24) | 0.427 | 0.997 |
| rs1341263 | 10 | 93556855 | *TNKS2-AS1* | G | A | 0.39 | 0.38 | 1.07 (0.91-1.25) | 0.427 | 0.997 |
| rs12567614 | 1 | 226544420 | - | A | G | 0.47 | 0.46 | 1.06 (0.91-1.24) | 0.432 | 0.997 |
| rs538800 | 11 | 94239567 | *MRE11* | A | G | 0.34 | 0.33 | 1.07 (0.91-1.26) | 0.433 | 0.997 |
| rs2975842 | 8 | 73925591 | *TERF1* | A | G | 0.43 | 0.45 | 0.94 (0.81-1.10) | 0.445 | 0.997 |
| rs1760897 | 14 | 20876253 | *TEP1* | G | A | 0.30 | 0.31 | 0.94 (0.80-1.10) | 0.445 | 0.997 |
| rs33994795 | 8 | 9473429 | *TNKS* | G | A | 0.38 | 0.40 | 0.94 (0.81-1.10) | 0.460 | 0.997 |
| rs11250076 | 8 | 10647823 | *PINX1* | A | G | 0.45 | 0.47 | 0.95 (0.81-1.10) | 0.476 | 0.997 |
| rs227060 | 11 | 108204881 | *ATM*, *C11orf65* | A | G | 0.33 | 0.32 | 1.06 (0.91-1.23) | 0.485 | 0.997 |
| rs2975852 | 8 | 73932430 | *TERF1* | A | G | 0.31 | 0.34 | 0.94 (0.80-1.12) | 0.488 | 0.997 |
| rs405684 | 17 | 56777148 | *RAD51C* | A | G | 0.36 | 0.39 | 0.95 (0.81-1.10) | 0.489 | 0.997 |
| rs2227933 | 15 | 91337479 | *BLM* | A | G | 0.19 | 0.18 | 1.07 (0.88-1.30) | 0.490 | 0.997 |
| rs2110159 | 12 | 21649160 | *RECQL* | G | A | 0.49 | 0.50 | 0.95 (0.81-1.11) | 0.507 | 0.997 |
| rs10102170 | 8 | 9611470 | *TNKS* | A | G | 0.37 | 0.39 | 0.95 (0.81-1.11) | 0.510 | 0.997 |
| rs2853672 | 5 | 1292983 | *TERT* | A | C | 0.48 | 0.50 | 1.05 (0.91-1.21) | 0.511 | 0.997 |
| rs7826180 | 8 | 10627844 | *PINX1*, *LOC102723313* | A | G | 0.23 | 0.22 | 1.06 (0.88-1.28) | 0.511 | 0.997 |
| rs11930711 | 4 | 110734498 | - | A | G | 0.09 | 0.09 | 1.09 (0.84-1.41) | 0.512 | 0.997 |
| rs820206 | 17 | 73641714 | *RECQL5* | A | G | 0.36 | 0.35 | 1.06 (0.90-1.24) | 0.519 | 0.997 |
| rs12334407 | 8 | 73953688 | *TERF1* | A | C | 0.30 | 0.32 | 0.95 (0.80-1.12) | 0.520 | 0.997 |
| rs11249930 | 8 | 9472445 | *TNKS* | G | A | 0.19 | 0.19 | 0.94 (0.76-1.15) | 0.524 | 0.997 |
| rs11211262 | 1 | 46721155 | *RAD54L* | A | G | 0.39 | 0.40 | 0.95 (0.81-1.11) | 0.524 | 0.997 |
| rs2238337 | 15 | 91355732 | *BLM* | A | G | 0.35 | 0.36 | 0.95 (0.81-1.12) | 0.533 | 0.997 |
| rs2104978 | 14 | 20837033 | *TEP1* | G | A | 0.07 | 0.06 | 1.10 (0.82-1.48) | 0.535 | 0.997 |
| rs3102095 | 8 | 73916745 | - | C | A | 0.30 | 0.32 | 0.95 (0.80-1.12) | 0.535 | 0.997 |
| rs2736100 | 5 | 1286516 | *TERT* | C | A | 0.49 | 0.50 | 0.96 (0.83-1.11) | 0.535 | 0.997 |
| rs12680047 | 8 | 128758861 | - | G | A | 0.39 | 0.39 | 0.95 (0.81-1.12) | 0.539 | 0.997 |
| rs2228041 | 14 | 20852267 | *TEP1* | A | G | 0.06 | 0.06 | 1.10 (0.81-1.50) | 0.540 | 0.997 |
| rs740059 | 12 | 4671490 | *LOC113939935*, *DYRK4* | G | A | 0.49 | 0.49 | 1.05 (0.90-1.22) | 0.540 | 0.997 |
| rs10849087 | 12 | 4650065 | *RAD51AP1* | A | G | 0.22 | 0.21 | 1.05 (0.89-1.25) | 0.547 | 0.997 |
| rs1713449 | 14 | 20841707 | *TEP1* | A | G | 0.22 | 0.24 | 0.95 (0.78-1.14) | 0.550 | 0.997 |
| rs7083959 | 10 | 93585997 | *TNKS2* | C | A | 0.19 | 0.17 | 1.06 (0.87-1.29) | 0.552 | 0.997 |
| rs1760903 | 14 | 20852817 | *TEP1* | A | G | 0.45 | 0.46 | 0.95 (0.82-1.11) | 0.553 | 0.997 |
| rs820210 | 17 | 73650495 | *LOC107985013*, *RECQL5* | G | A | 0.36 | 0.35 | 1.05 (0.89-1.23) | 0.554 | 0.997 |
| rs2297437 | 20 | 62305274 | *RTEL1*, *RTEL1-TNFRSF6B* | A | G | 0.17 | 0.20 | 0.94 (0.77-1.15) | 0.556 | 0.997 |
| rs2237060 | 5 | 131970885 | *TH2-LCR*, *RAD50*, *TH2LCRR* | C | A | 0.37 | 0.37 | 0.95 (0.81-1.12) | 0.562 | 0.997 |
| rs2299014 | 5 | 131903399 | *RAD50* | C | A | 0.40 | 0.41 | 0.95 (0.81-1.12) | 0.564 | 0.997 |
| rs4427176 | 8 | 9533399 | *TNKS* | A | C | 0.18 | 0.19 | 0.94 (0.77-1.16) | 0.573 | 0.997 |
| rs3755130 | 2 | 15729583 | - | A | G | 0.41 | 0.42 | 0.96 (0.83-1.11) | 0.575 | 0.997 |
| rs228591 | 11 | 108097333 | *ATM* | A | G | 0.43 | 0.44 | 0.96 (0.82-1.12) | 0.581 | 0.997 |
| rs3219142 | 1 | 226552068 | *PARP1* | A | G | 0.20 | 0.19 | 1.05 (0.88-1.27) | 0.584 | 0.997 |
| rs476137 | 11 | 94213905 | *MRE11* | A | C | 0.43 | 0.43 | 1.04 (0.90-1.22) | 0.586 | 0.997 |
| rs3219038 | 1 | 226578809 | *PARP1* | A | G | 0.11 | 0.11 | 0.94 (0.74-1.19) | 0.591 | 0.997 |
| rs2292370 | 8 | 10690319 | *PINX1* | A | G | 0.29 | 0.30 | 0.96 (0.80-1.14) | 0.614 | 0.997 |
| rs909341 | 20 | 62328742 | *TNFRSF6B*, *RTEL1-TNFRSF6B* | A | G | 0.23 | 0.22 | 1.05 (0.87-1.26) | 0.617 | 0.997 |
| rs1805812 | 8 | 90965053 | *NBN* | G | A | 0.10 | 0.09 | 1.07 (0.82-1.39) | 0.622 | 0.997 |
| rs13447720 | 11 | 94165326 | *MRE11* | G | A | 0.22 | 0.23 | 0.96 (0.79-1.15) | 0.632 | 0.997 |
| rs10464529 | 7 | 124520308 | *POT1* | A | G | 0.27 | 0.26 | 1.04 (0.88-1.23) | 0.637 | 0.997 |
| rs114939615 | 1 | 226591587 | *PARP1* | A | G | 0.07 | 0.07 | 1.07 (0.80-1.43) | 0.646 | 0.997 |
| rs10099824 | 8 | 73940350 | *TERF1* | A | G | 0.47 | 0.45 | 1.04 (0.89-1.21) | 0.648 | 0.997 |
| rs709816 | 8 | 90967711 | *NBN* | G | A | 0.38 | 0.39 | 0.97 (0.83-1.13) | 0.652 | 0.997 |
| rs663530 | 11 | 94157114 | *MRE11* | A | G | 0.26 | 0.26 | 0.96 (0.80-1.15) | 0.652 | 0.997 |
| rs9956832 | 18 | 39564876 | *PIK3C3* | A | C | 0.14 | 0.13 | 1.05 (0.84-1.32) | 0.656 | 0.997 |
| rs1805793 | 8 | 90990091 | *NBN* | G | A | 0.38 | 0.39 | 0.97 (0.83-1.13) | 0.656 | 0.997 |
| rs4585 | 11 | 108239628 | *ATM*, *C11orf65* | C | A | 0.43 | 0.44 | 0.96 (0.82-1.13) | 0.657 | 0.997 |
| rs604845 | 11 | 94182689 | *MRE11* | A | G | 0.34 | 0.33 | 1.04 (0.88-1.22) | 0.657 | 0.997 |
| rs1048771 | 1 | 46743900 | *LRRC41*, *RAD54L* | A | G | 0.10 | 0.11 | 0.95 (0.75-1.20) | 0.662 | 0.997 |
| rs11542623 | 1 | 46746164 | *LRRC41* | A | G | 0.10 | 0.11 | 0.95 (0.75-1.20) | 0.663 | 0.997 |
| rs619972 | 11 | 108169619 | *ATM* | G | A | 0.44 | 0.44 | 0.97 (0.82-1.13) | 0.665 | 0.997 |
| rs6601338 | 8 | 9438928 | *TNKS* | A | G | 0.37 | 0.39 | 0.97 (0.83-1.13) | 0.667 | 0.997 |
| rs12125573 | 1 | 46734792 | *RAD54L* | G | A | 0.29 | 0.30 | 0.97 (0.82-1.14) | 0.670 | 0.997 |
| rs645485 | 11 | 108168863 | *ATM* | A | G | 0.43 | 0.44 | 0.97 (0.82-1.13) | 0.673 | 0.997 |
| rs1805796 | 8 | 90993395 | *NBN* | A | G | 0.38 | 0.39 | 0.97 (0.83-1.13) | 0.674 | 0.997 |
| rs3017077 | 11 | 94175249 | *MRE11* | A | G | 0.33 | 0.34 | 0.97 (0.82-1.14) | 0.676 | 0.997 |
| rs3213212 | 12 | 21635232 | *RECQL* | A | G | 0.43 | 0.45 | 0.97 (0.83-1.13) | 0.676 | 0.997 |
| rs3761124 | 20 | 62288752 | - | A | G | 0.23 | 0.21 | 1.04 (0.86-1.26) | 0.677 | 0.997 |
| rs938886 | 14 | 20837701 | *TEP1* | G | C | 0.23 | 0.24 | 0.96 (0.80-1.16) | 0.679 | 0.997 |
| rs654718 | 11 | 94190115 | *MRE11* | G | A | 0.34 | 0.34 | 0.97 (0.82-1.14) | 0.684 | 0.997 |
| rs61835377 | 1 | 226545854 | - | A | G | 0.19 | 0.20 | 0.96 (0.79-1.17) | 0.686 | 0.997 |
| rs600931 | 11 | 108117335 | *ATM* | G | A | 0.43 | 0.44 | 0.97 (0.83-1.14) | 0.695 | 0.997 |
| rs6668851 | 1 | 226545866 | - | C | A | 0.29 | 0.30 | 0.97 (0.81-1.15) | 0.695 | 0.997 |
| rs599558 | 11 | 108177538 | *ATM* | G | A | 0.43 | 0.44 | 0.97 (0.83-1.14) | 0.705 | 0.997 |
| rs4733220 | 8 | 30900890 | *WRN* | A | G | 0.42 | 0.42 | 0.97 (0.84-1.13) | 0.705 | 0.997 |
| rs8022805 | 14 | 20846950 | *TEP1* | A | G | 0.06 | 0.05 | 1.06 (0.78-1.45) | 0.707 | 0.997 |
| rs17772565 | 5 | 131952405 | *RAD50* | A | G | 0.05 | 0.06 | 0.94 (0.66-1.34) | 0.710 | 0.997 |
| rs9822885 | 3 | 169486144 | *ACTRT3* | G | A | 0.28 | 0.29 | 0.97 (0.81-1.16) | 0.711 | 0.997 |
| rs12638862 | 3 | 169477506 | - | G | A | 0.26 | 0.28 | 0.97 (0.81-1.16) | 0.714 | 0.997 |
| rs2736122 | 5 | 1257621 | *TERT* | A | G | 0.24 | 0.24 | 0.97 (0.81-1.15) | 0.720 | 0.997 |
| rs12630450 | 3 | 169480204 | - | G | A | 0.28 | 0.30 | 0.97 (0.81-1.16) | 0.723 | 0.997 |
| rs13273033 | 8 | 9540693 | *TNKS* | G | A | 0.26 | 0.27 | 0.97 (0.81-1.15) | 0.723 | 0.997 |
| rs7015700 | 8 | 9527707 | *TNKS* | A | G | 0.19 | 0.20 | 0.97 (0.79-1.18) | 0.730 | 0.997 |
| rs609261 | 11 | 108158134 | *ATM* | A | G | 0.44 | 0.44 | 0.97 (0.83-1.14) | 0.731 | 0.997 |
| rs1713456 | 14 | 20850093 | *TEP1* | A | G | 0.20 | 0.19 | 1.03 (0.86-1.25) | 0.734 | 0.997 |
| rs389480 | 15 | 91351930 | *BLM* | A | G | 0.46 | 0.46 | 1.03 (0.88-1.20) | 0.747 | 0.997 |
| rs6010620 | 20 | 62309839 | *RTEL1*, *RTEL1-TNFRSF6B* | A | G | 0.23 | 0.22 | 1.03 (0.85-1.25) | 0.751 | 0.997 |
| rs10090277 | 8 | 9450886 | *TNKS* | G | A | 0.08 | 0.08 | 1.05 (0.78-1.40) | 0.763 | 0.997 |
| rs3784780 | 15 | 91309475 | *BLM* | A | G | 0.17 | 0.16 | 1.03 (0.84-1.27) | 0.769 | 0.997 |
| rs78733221 | 8 | 9476448 | *TNKS* | G | A | 0.08 | 0.08 | 1.04 (0.78-1.39) | 0.769 | 0.997 |
| rs6601333 | 8 | 9427048 | *TNKS* | G | A | 0.26 | 0.26 | 1.03 (0.86-1.22) | 0.772 | 0.997 |
| rs623860 | 11 | 108106782 | *ATM* | G | A | 0.42 | 0.42 | 0.98 (0.83-1.15) | 0.782 | 0.997 |
| rs2897443 | 5 | 131929594 | *RAD50* | A | C | 0.22 | 0.21 | 1.03 (0.86-1.23) | 0.788 | 0.997 |
| rs4246977 | 14 | 20882591 | - | G | A | 0.38 | 0.38 | 0.98 (0.84-1.14) | 0.790 | 0.997 |
| rs11785739 | 8 | 9640400 | - | A | C | 0.08 | 0.08 | 1.04 (0.78-1.38) | 0.795 | 0.997 |
| rs1882928 | 8 | 31023822 | *WRN* | G | A | 0.41 | 0.42 | 0.98 (0.84-1.15) | 0.803 | 0.997 |
| rs6470522 | 8 | 90954481 | *NBN* | A | G | 0.18 | 0.17 | 1.03 (0.84-1.25) | 0.807 | 0.997 |
| rs17711777 | 8 | 10623240 | *PINX1*, *LOC102723313* | G | A | 0.07 | 0.08 | 0.97 (0.73-1.28) | 0.810 | 0.997 |
| rs2192170 | 12 | 21633036 | *RECQL* | A | G | 0.09 | 0.09 | 0.97 (0.75-1.26) | 0.813 | 0.997 |
| rs3219125 | 1 | 226554951 | *PARP1* | G | A | 0.06 | 0.06 | 1.04 (0.76-1.41) | 0.819 | 0.997 |
| rs7162960 | 15 | 91287412 | *BLM* | G | A | 0.23 | 0.22 | 1.02 (0.85-1.22) | 0.820 | 0.997 |
| rs7159947 | 14 | 20809491 | - | G | A | 0.36 | 0.36 | 0.98 (0.85-1.14) | 0.821 | 0.997 |
| rs2508678 | 11 | 94149349 | *MRE11* | A | G | 0.37 | 0.37 | 0.98 (0.84-1.15) | 0.825 | 0.997 |
| rs1801516 | 11 | 108175462 | *ATM* | A | G | 0.16 | 0.16 | 1.02 (0.83-1.26) | 0.829 | 0.997 |
| rs1760898 | 14 | 20872881 | *TEP1* | A | C | 0.23 | 0.23 | 0.98 (0.81-1.18) | 0.830 | 0.997 |
| rs2706348 | 5 | 131905810 | *RAD50* | A | G | 0.23 | 0.22 | 1.02 (0.85-1.22) | 0.837 | 0.997 |
| rs3755133 | 2 | 15731583 | - | A | G | 0.17 | 0.17 | 1.02 (0.82-1.27) | 0.847 | 0.997 |
| rs10492117 | 12 | 21659368 | *GOLT1B* | G | A | 0.50 | 0.50 | 0.99 (0.84-1.15) | 0.849 | 0.997 |
| rs2706347 | 5 | 131905117 | *RAD50* | A | C | 0.23 | 0.22 | 1.02 (0.85-1.22) | 0.852 | 0.997 |
| rs12679892 | 8 | 9445429 | *TNKS* | G | A | 0.31 | 0.31 | 1.02 (0.86-1.20) | 0.854 | 0.997 |
| rs10954778 | 8 | 31008747 | *WRN* | G | A | 0.32 | 0.31 | 1.02 (0.86-1.20) | 0.855 | 0.997 |
| rs9939870 | 16 | 69396585 | *TERF2* | A | G | 0.24 | 0.25 | 0.99 (0.84-1.16) | 0.860 | 0.997 |
| rs2158177 | 5 | 131984058 | *TH2LCRR* | G | A | 0.21 | 0.21 | 1.02 (0.85-1.22) | 0.861 | 0.997 |
| rs6011011 | 20 | 62299578 | *RTEL1*, *RTEL1-TNFRSF6B* | A | G | 0.08 | 0.07 | 1.03 (0.76-1.38) | 0.866 | 0.997 |
| rs7737470 | 5 | 131974063 | *TH2-LCR*, *RAD50*, *TH2LCRR* | T | A | 0.23 | 0.22 | 1.02 (0.85-1.22) | 0.870 | 0.997 |
| rs917855 | 12 | 21630146 | *RECQL* | A | G | 0.50 | 0.49 | 0.99 (0.85-1.15) | 0.870 | 0.997 |
| rs3848668 | 20 | 62293272 | *RTEL1*, *RTEL1-TNFRSF6B* | G | A | 0.07 | 0.07 | 1.03 (0.76-1.39) | 0.870 | 0.997 |
| rs12653750 | 5 | 131971902 | *TH2-LCR*, *RAD50*, *TH2LCRR* | A | G | 0.22 | 0.22 | 1.02 (0.85-1.22) | 0.872 | 0.997 |
| rs2107465 | 8 | 91001024 | - | A | G | 0.30 | 0.30 | 1.01 (0.86-1.19) | 0.873 | 0.997 |
| rs11776767 | 8 | 10683929 | *PINX1* | C | G | 0.34 | 0.34 | 0.99 (0.83-1.18) | 0.875 | 0.997 |
| rs664143 | 11 | 108225661 | *ATM*, *C11orf65* | A | G | 0.43 | 0.43 | 0.99 (0.84-1.16) | 0.877 | 0.997 |
| rs1772188 | 10 | 93616628 | *TNKS2* | A | C | 0.15 | 0.14 | 0.98 (0.79-1.22) | 0.879 | 0.997 |
| rs8031341 | 15 | 91313224 | *BLM* | G | A | 0.20 | 0.21 | 0.99 (0.82-1.19) | 0.885 | 0.997 |
| rs7834823 | 8 | 9437352 | *TNKS* | A | C | 0.26 | 0.26 | 1.01 (0.85-1.21) | 0.891 | 0.997 |
| rs4809324 | 20 | 62318220 | *RTEL1*, *RTEL1-TNFRSF6B* | G | A | 0.10 | 0.11 | 0.98 (0.76-1.27) | 0.893 | 0.997 |
| rs12545912 | 8 | 9601699 | *TNKS* | C | A | 0.27 | 0.27 | 0.99 (0.83-1.17) | 0.894 | 0.997 |
| rs1805414 | 1 | 226573364 | *PARP1* | G | A | 0.36 | 0.37 | 1.01 (0.86-1.19) | 0.899 | 0.997 |
| rs1800391 | 8 | 30938704 | *WRN* | A | G | 0.08 | 0.08 | 1.02 (0.76-1.37) | 0.900 | 0.997 |
| rs2244012 | 5 | 131901225 | *RAD50* | G | A | 0.23 | 0.23 | 1.01 (0.85-1.21) | 0.908 | 0.997 |
| rs1805818 | 8 | 90970862 | *NBN* | A | C | 0.33 | 0.33 | 1.01 (0.86-1.18) | 0.918 | 0.997 |
| rs2240032 | 5 | 131977127 | *TH2-LCR*, *RAD50*, *TH2LCRR* | A | G | 0.22 | 0.22 | 1.01 (0.84-1.21) | 0.918 | 0.997 |
| rs1805794 | 8 | 90990479 | *NBN* | C | G | 0.33 | 0.33 | 1.01 (0.86-1.18) | 0.923 | 0.997 |
| rs16943176 | 17 | 56769887 | *RAD51C* | A | G | 0.23 | 0.22 | 1.01 (0.85-1.20) | 0.923 | 0.997 |
| rs6871536 | 5 | 131969874 | *TH2-LCR*, *RAD50*, *TH2LCRR* | G | A | 0.23 | 0.22 | 1.01 (0.84-1.21) | 0.931 | 0.997 |
| rs2073635 | 8 | 90995366 | *NBN* | A | G | 0.33 | 0.33 | 1.01 (0.86-1.18) | 0.937 | 0.997 |
| rs622961 | 11 | 94154902 | *MRE11* | A | G | 0.44 | 0.44 | 1.01 (0.86-1.17) | 0.937 | 0.997 |
| rs1063045 | 8 | 90995019 | *NBN* | A | G | 0.33 | 0.33 | 1.01 (0.86-1.18) | 0.937 | 0.997 |
| rs3219090 | 1 | 226564691 | *PARP1* | A | G | 0.36 | 0.37 | 0.99 (0.85-1.17) | 0.940 | 0.997 |
| rs2725385 | 8 | 30928146 | *WRN* | A | G | 0.29 | 0.28 | 0.99 (0.84-1.18) | 0.944 | 0.997 |
| rs3093933 | 14 | 20824415 | *PARP2* | A | C | 0.26 | 0.25 | 1.01 (0.85-1.19) | 0.944 | 0.997 |
| rs2297434 | 20 | 62294015 | *RTEL1*, *RTEL1-TNFRSF6B* | G | A | 0.47 | 0.48 | 1.01 (0.86-1.17) | 0.945 | 0.997 |
| rs1346044 | 8 | 31024654 | *WRN* | G | A | 0.27 | 0.26 | 1.00 (0.84-1.18) | 0.954 | 0.997 |
| rs2040704 | 5 | 131973177 | *TH2-LCR*, *RAD50*, *TH2LCRR* | G | A | 0.23 | 0.23 | 1.00 (0.83-1.19) | 0.957 | 0.997 |
| rs2301713 | 5 | 131951996 | *RAD50* | G | A | 0.23 | 0.22 | 1.01 (0.84-1.20) | 0.958 | 0.997 |
| rs6596086 | 5 | 131952222 | *RAD50* | G | A | 0.23 | 0.23 | 1.01 (0.84-1.20) | 0.961 | 0.997 |
| rs976016 | 2 | 15772865 | - | G | A | 0.18 | 0.17 | 1.01 (0.81-1.24) | 0.962 | 0.997 |
| rs6982126 | 8 | 73939623 | *TERF1* | A | G | 0.23 | 0.23 | 1.00 (0.83-1.19) | 0.963 | 0.997 |
| rs444325 | 15 | 91364015 | - | A | C | 0.34 | 0.35 | 1.00 (0.85-1.18) | 0.969 | 0.997 |
| rs3755132 | 2 | 15729820 | - | C | A | 0.16 | 0.16 | 1.00 (0.80-1.24) | 0.978 | 0.997 |
| rs2234744 | 8 | 90970935 | *NBN* | A | G | 0.33 | 0.33 | 1.00 (0.86-1.17) | 0.981 | 0.997 |
| rs12187537 | 5 | 131939904 | *RAD50* | C | A | 0.21 | 0.21 | 1.00 (0.84-1.20) | 0.982 | 0.997 |
| rs425538 | 11 | 108219339 | *ATM*, *C11orf65* | C | A | 0.43 | 0.43 | 1.00 (0.85-1.17) | 0.988 | 0.997 |
| rs2074369 | 5 | 131973663 | *TH2-LCR*, *RAD50*, *TH2LCRR* | G | A | 0.23 | 0.23 | 1.00 (0.84-1.20) | 0.992 | 0.997 |
| rs3798134 | 5 | 131965179 | *TH2-LCR*, *RAD50* | A | G | 0.23 | 0.23 | 1.00 (0.83-1.20) | 0.994 | 0.997 |
| rs2853677 | 5 | 1287194 | *LOC110806264*, *TERT* | G | A | 0.41 | 0.41 | 1.00 (0.87-1.16) | 0.997 | 0.997 |

Chromosomal positions are based on the Genome Reference Consortium Human Build 37 (GRCh37) of the single nucleotide polymorphism database (dbSNP) build 151, MAF; Minor allele frequency, FDR; False discovery rate.

**References**

1 Nersisyan, L. & Arakelyan, A. Computel: computation of mean telomere length from whole-genome next-generation sequencing data. *PLoS One* **10**, e0125201, doi:10.1371/journal.pone.0125201 (2015).

2 Ding, Z. *et al.* Estimating telomere length from whole genome sequence data. *Nucleic Acids Res* **42**, e75, doi:10.1093/nar/gku181 (2014).

3 Farmery, J. H. R., Smith, M. L., Diseases, N. B.-R. & Lynch, A. G. Telomerecat: A ploidy-agnostic method for estimating telomere length from whole genome sequencing data. *Sci Rep* **8**, 1300, doi:10.1038/s41598-017-14403-y (2018).

4 Lee, M. *et al.* Comparative analysis of whole genome sequencing-based telomere length measurement techniques. *Methods* **114**, 4-15, doi:10.1016/j.ymeth.2016.08.008 (2017).
